# Multimodal single-cell datasets characterize antigen-specific CD8^+^ T cells across SARS-CoV-2 vaccination and infection

**DOI:** 10.1038/s41590-023-01608-9

**Published:** 2023-09-21

**Authors:** Bingjie Zhang, Rabi Upadhyay, Yuhan Hao, Marie I. Samanovic, Ramin S. Herati, John D. Blair, Jordan Axelrad, Mark J. Mulligan, Dan R. Littman, Rahul Satija

**Affiliations:** 1 New York Genome Center, New York, NY USA; 2 Center for Genomics and Systems Biology, New York University, New York, NY USA; 3 Department of Cell Biology, New York University Grossman School of Medicine, New York, NY USA; 4 Perlmutter Cancer Center, New York University Langone Health, New York, NY USA; 5 Department of Medicine, New York University Grossman School of Medicine, New York, NY USA; 6 New York University Langone Vaccine Center, New York, NY USA; 7 Howard Hughes Medical Institute, New York, NY USA

**Keywords:** Immunology, Next-generation sequencing

## Abstract

The immune response to SARS-CoV-2 antigen after infection or vaccination is defined by the durable production of antibodies and T cells. Population-based monitoring typically focuses on antibody titer, but there is a need for improved characterization and quantification of T cell responses. Here, we used multimodal sequencing technologies to perform a longitudinal analysis of circulating human leukocytes collected before and after immunization with the mRNA vaccine BNT162b2. Our data indicated distinct subpopulations of CD8^+^ T cells, which reliably appeared 28 days after prime vaccination. Using a suite of cross-modality integration tools, we defined their transcriptome, accessible chromatin landscape and immunophenotype, and we identified unique biomarkers within each modality. We further showed that this vaccine-induced population was SARS-CoV-2 antigen-specific and capable of rapid clonal expansion. Moreover, we identified these CD8^+^ T cell populations in scRNA-seq datasets from COVID-19 patients and found that their relative frequency and differentiation outcomes were predictive of subsequent clinical outcomes.

## Abstract (web summary)

Satija and colleagues use multimodal sequencing technologies and cross-modality integration tools to define distinct subpopulations of CD8^+^ T cells that are predictive of COVID-19 severity.

## Main

The coronavirus disease 2019 (COVID-19) pandemic has been a global public health challenge, yet mRNA vaccines effectively protect against severe disease^1,2^. Immune responses elicited by severe acute respiratory syndrome coronavirus 2 (SARS-CoV-2) mRNA vaccines are typically assessed through titers of neutralizing antibodies, which rise rapidly after vaccination boosts but decline after 3–6 months^3,4^. However, cellular immunity, mediated in part by CD4^+^ and CD8^+^ T cells, has a critical role in viral clearance and protection^5^. Vaccine-induced T cells were reported to protect against COVID-19 even without antibody responses^6^. A deeper understanding of the distinct subpopulations that drive cellular immunity will be essential for interpreting individual immune responses and for informing public health strategies^7^.

Antigen-specific T cells are conventionally identified by cytokine profiling or by labeling with peptide–major histocompatibility complex (pMHC) multimers. Both assays can be multiplexed with additional surface proteins for flow cytometry^8^. Multiple studies have applied these approaches to investigate SARS-CoV-2 mRNA vaccine responses, focusing on the kinetics of antigen-specific T cell proliferation and surface marker characterization^4,5,9,10^. Longitudinal profiling of human peripheral blood mononuclear cells (PBMCs) followed by pMHC-I tetramer enrichment showed an initial surge of antigen-specific CD8^+^T cells after vaccination, then contraction as cells differentiated over 3–4 months^5^. Ex vivo activation experiments demonstrated similar kinetics and highlighted the potentially limited sensitivity of these assays to quantify rare CD8^+^ cells^4,11,12^.

Single-cell RNA-sequencing (scRNA-seq) assays are, in principle, well suited for characterization of cellular responses. Moreover, single-cell sequencing assays enable unsupervised identification of cell states directly from PBMC samples, without need for ex vivo restimulation to reveal pre-established immunophenotypic markers of differentiation or specificity for particular HLA haplotypes. Despite advancements in scRNA-seq assays, detecting rare or subtle cell states from sparse and noisy datasets remains challenging. A study on COVID mRNA vaccine responses revealed activation and proliferation in myeloid clusters but failed to identify antigen-specific T cell subsets^13^.

Here, we performed a longitudinal analysis of human PBMCs from a SARS-CoV-2 mRNA vaccination series using a suite of multimodal single-cell sequencing technologies. Moving beyond the transcriptome, we additionally measured chromatin accessibility, surface protein abundance, immune receptor repertoires and pMHC-multimer-binding modalities. By leveraging computational tools for within- and across-modality integration, we identified specific groups of vaccine-induced effector memory CD8^+^ T cells in each dataset. This enabled us to delineate high-resolution subpopulations and biomarkers within each modality, validate their clonal identity and antigen-specificity and identify their developmental regulators. By integrating our datasets with single-cell datasets of natural SARS-CoV-2 infection, we tracked the temporal differentiation patterns of these cells and showed that their quantitative abundance was strongly associated with recovery from severe disease.

## Results

### CITE-seq identifies vaccine-induced CD8^+^ T cell subsets

**Fig. 1:**
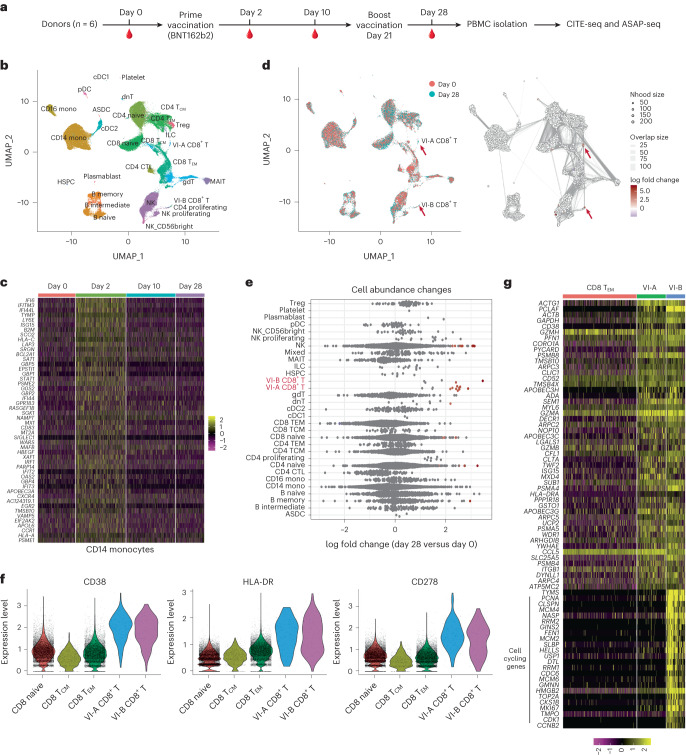
Multimodal identification of SARS-CoV-2 mRNA vaccine-induced CD8^+^ T cells. **a**, Overview of study design, in which PBMCs were collected from six healthy donors at day 0, day 2, day 10 and day 28 during the BNT162b2 mRNA vaccination process and comprehensive analyses using both CITE-seq and ASAP-seq were performed on these samples. **b**, Uniform manifold approximation and projection (UMAP) visualizations of 113,897 single cells obtained as in **a** profiled with CITE-seq and clustered by a weighted combination of RNA and protein modalities. Cells are colored based on level-2 annotation (level-1 and level-3 annotations; Extended Data Fig. 2a). **c**, Single-cell heatmap showing activation of interferon response module within CD14^+^ monocytes. **d**, Milo analysis of differentially abundant cell states between day 0 and day 28 samples as in panel a. UMAP on the left is color coded by timepoint. Right plot indicates embedding of the Milo differential abundance. Each node represents a neighborhood, node size is proportional to the number of cells, and neighborhoods are colored by the level of differential abundance. Nhood, neighborhood. **e**, Beeswarm plot showing the log-fold distribution of cell abundance changes between day 0 and day 28 samples as in **a**. Neighborhoods overlapping the same cell population are grouped together, neighborhoods exhibiting differential abundance are colored in red. **f**, Violin plots with protein upregulation of CD38, HLA-DR and CD278 (ICOS) in VI-A and VI-B CD8^+^ T cells compared with CD8^+^ naive, CD8^+^ T_CM_ and CD8^+^ T_EM_ cells. **g**, Heatmap showing mRNA expression of 50 marker genes for VI-A CD8^+^ T cells, as well as cell-cycling genes highly expressed in VI-B CD8^+^ T cells. For visualization purposes, a randomly selected subset of CD8^+^ T_EM_ cells are presented. HSPC, hematopoietic stem and progenitor cell; NK, natural killer; pDC, plasma dendritic cell; T_CM_, central memory T cell; T_EM_, effector memory T cell. ASDC, AXL^+^ dendritic cell; CTL, cytotoxic T cell; dnT, double-negative T cell; gdT, gamma-delta T cell; MAIT, mucosal associated invariant T.

To investigate immune responses to SARS-CoV-2 mRNA vaccination at single-cell resolution, between January and April 2021, we recruited six healthy donors with no self-reported previous experience with SARS-CoV-2 infection and analyzed circulating PBMCs at four timepoints over a time course of BNT162b2 mRNA vaccination: immediately before vaccination (day 0), after primary vaccination (day 2, day 10) and 7 days after boost vaccination (day 28) (Methods and Supplementary Table 1). For each of the 24 samples, we performed two multimodal single-cell sequencing assays: cellular indexing of transcriptomes and epitopes by sequencing (CITE-seq) for simultaneous measurement of cellular transcriptomes and surface proteins^14^ and ATAC with select antigen profiling by sequencing (ASAP-seq) for simultaneous profiling of open chromatin regions alongside cell surface proteins^15^ (Fig. 1a). For each assay, we used an optimized panel of oligo-conjugated antibodies (‘TotalSeq-A’ panels; Methods and Supplementary Table 2) along with the inclusion of additional markers. Our initial dataset represented 113,897 single cells in the scRNA-seq dataset and 78,677 single cells in the scASAP-seq dataset.

**Extended Data Fig. 1:**
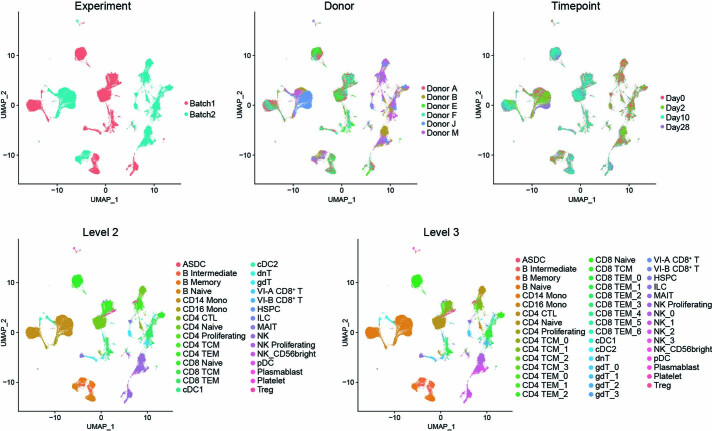
UMAP visualizations of CITE-seq dataset without integration. UMAP visualizations of 113,897 single cells profiled with CITE-seq and clustered on the weighted combination of both RNA and protein modalities without performing data integration. Cells are colored with experiment, donor, timepoint, level 2 or level 3 annotations.

**Extended Data Fig. 2:**
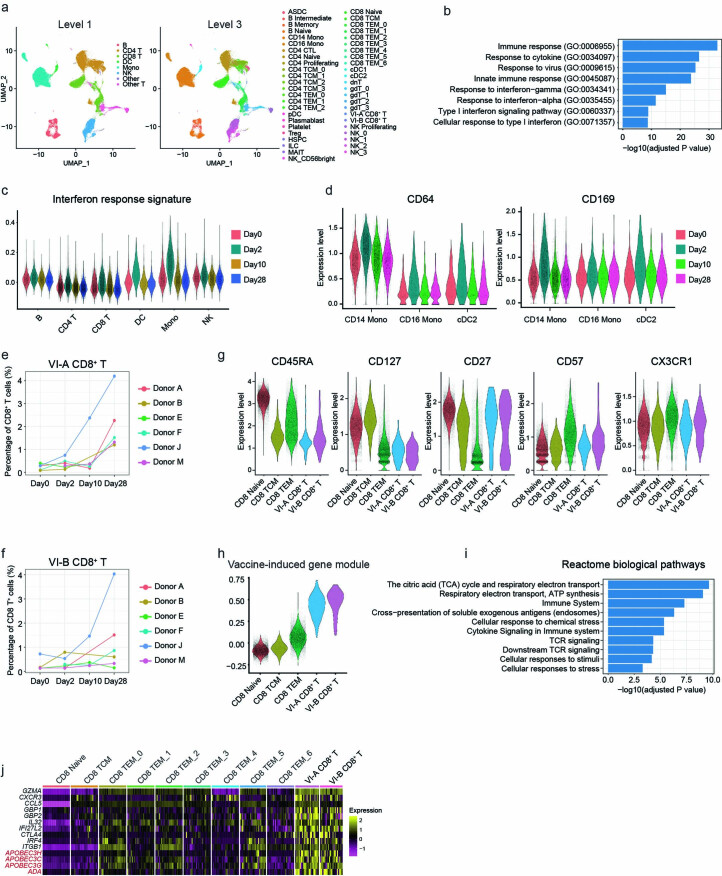
Unveiling unique molecular signatures of vaccine-induced CD8^+^ T cells via CITE-seq analysis. **a**, UMAP visualizations of 113,897 single cells profiled with CITE-seq and clustered on the weighted combination of both RNA and protein modalities. Cells are colored with either level 1 or level 3 annotations. **b**, Enriched GO terms for activated (Day 2 vs Day 0) genes in CD14^+^ Monocytes. **c**, Violin plots of interferon response signatures in selected cell types across four timepoints. P values were adjusted for multiple testing correction. **d**, Violin plots of protein expression of CD64 and CD169 in single cells in selected cell types, across four timepoints. **e-f**, Percentage of CD8^+^ T cells in vaccine-induced groups for each donor across four timepoints. **g**, Violin plots showing the protein expression of CD45RA, CD127, CD27, CD57 and CXC3R1 in selected cell types. **h**, Violin plots comparing gene module scores in VI-A and VI-B CD8^+^ T cells, as well as selected other subsets. **i**, Enriched reactome biological pathways for the 197 signature vaccine-induced gene set. P values were adjusted for multiple testing correction. **j**, Heatmap showing the expression of select genes in CD8^+^ T cell subtypes. The visualization presents pseudobulk averages, with cells grouped by cell type, individual human donor, and timepoints, and demonstrates that marker genes are reproducible across donors.

We first explored the CITE-seq datasets by applying our ‘anchor-based’ integration workflow to match together cells in shared biological states across individuals and timepoints^16,17^. Although this caused shared cell types in pre-vaccination and post-vaccination datasets to cluster together initially (Extended Data Fig. 1), integration enabled us to consistently annotate these cell states across samples and ensure the results were not driven by effects from one individual donor. To cluster cells, we applied weighted-nearest neighbor (WNN) analysis (Methods), which defines cell states jointly based on a weighted combination of RNA and protein modalities^17^. WNN analysis improved the identification of cell states for multimodal technologies such as CITE-seq by simultaneously leveraging the unsupervised nature of transcriptomic data with the robust protein measurements from oligo-tagged CITE-seq antibodies^17^. We annotated clusters at three different levels of resolution (Fig. 1b, Extended Data Fig. 2a and Methods).

Comparison of sample expression profiles across timepoints indicated a strong activation of interferon-signaling pathways originating at day 2 and dampened at day 10 and day 28 (Fig. 1c and Extended Data Fig. 2b), consistent with previous studies^11,13^. This response was most strongly activated in innate immune response components but was weakly detectable in lymphoid cell types as well (Extended Data Fig. 2c). The mRNA vaccine-responsive gene set was accompanied by the clear upregulation of cell surface protein biomarkers, including CD64 and CD169, in myeloid cell types^17^ (Extended Data Fig. 2d). We next explored the changes in cellular density and abundance across the four vaccination timepoints and identified two subsets of CD8^+^ T cells. ‘Vaccine-induced group A CD8^+^ T cells’ (hereafter VI-A CD8^+^ T cells) and ‘vaccine-induced group B CD8^+^ T cells’ (VI-B CD8^+^ T cells; Fig. 1d,e) were minimally present in day 0 samples but increased in abundance moderately after primary vaccination (day 2), and sharply at day 28 (after boost vaccination) across multiple donors (Extended Data Fig. 2e,f). We observed consistent results using either cluster-based differential abundance testing or alternately, using Milo, a framework for identifying differences in cellular density without reliance on cellular labels^18^ (Fig. 1d,e). We observed only mild changes in cellular density among CD4^+^ T cell subgroups when comparing samples between day 0 and day 28 (Fig. 1d,e), likely due to the differential kinetics of CD4^+^ and CD8^+^ T cell responses^5,19^.

Both VI-A and VI-B CD8^+^ T cell subsets exhibited upregulation of protein biomarkers previously associated with activation during antigen-specific responses^5,20^, including CD38, HLA-DR, and CD278 (ICOS) (Fig. 1f and Extended Data Fig. 2g). Inclusion of protein data using WNN analysis was essential for identifying and defining these subgroups, as they were not readily identifiable using unsupervised analysis of the transcriptomic data alone. Once identified, differential analysis revealed that VI-A and VI-B CD8^+^ T cells differed primarily in the expression of cell cycle genes (Fig. 1g), whereas a module of 197 genes (referred to hereafter as VI-GEM) was consistently upregulated across both groups (Fig. 1g, Extended Data Fig. 2h and Supplementary Table 3). This gene set was enriched for cytotoxic effector, TCR signaling, antigen processing and metabolic and respiratory gene categories (Extended Data Fig. 2i). We also observed an upregulation of multiple deaminase proteins (such as APOBEC3H, APOBEC3G, APOBEC3C and ADA), that can introduce mutations as part of the antiviral response^21,22^ (Fig. 1g and Extended Data Fig. 2j). These observations indicated the presence of a proliferative (VI-B) and non-proliferative (VI-A) CD8^+^ T cell populations, and we also discriminated between proliferative responses (unique to VI-B) and activation responses (shared between VI-A and VI-B), which might otherwise blend together.

**Extended Data Fig. 3:**
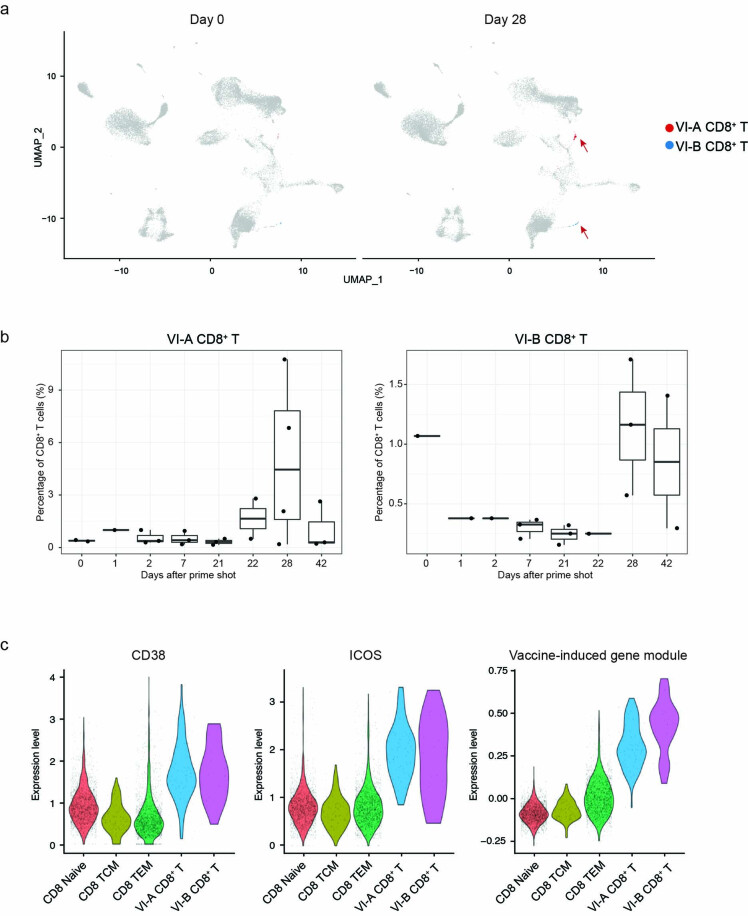
Detection of underrepresented vaccine-induced CD8^+^ T cells in a published dataset via supervised reference mapping. **a**, UMAP visualization of CITE-seq data derived from human PBMC from ref. ^13^ on day 0 and day 28, after reference mapping to the CITE-seq data in Fig. 1b. Cells matching gene signature for VI-A and VI-B CD8^+^ T cells are highlighted in red and blue. **b**, Boxplots showing the percentage of CD8^+^ T cells that fall in VI-A CD8^+^ T cells (left) or VI-B CD8^+^ T cells (right) for each donor across eight timepoints. Each dot represents one donor. The dataset is comprised of samples from n = 5 individual donors. Box center lines, bounds of boxes and whiskers indicate median, first and third quartiles and minima and maxima within a 1.5× interquartile range (IQR), respectively. **c**, Violin plots showing protein expression of CD38 and ICOS, along with a gene module score for vaccine-induced cells in selected CD8^+^ T cell subsets.

For additional validation, we reanalyzed a previously published CITE-seq dataset profiling a similar SARS-CoV-2 mRNA vaccination time course across six individuals^13^. Although the original study^13^ did not identify populations of vaccine-induced CD8^+^ T cells in unsupervised transcriptomic analysis, we tested if supervised reference mapping workflows had higher power to detect subtle cell states. Mapping the query onto our newly generated reference identified both VI-A and VI-B CD8^+^ T cell populations (Extended Data Fig. 3a) and showed they sharply increased in frequency at day 28 (Extended Data Fig. 3b), upregulated the expression of CD38 and ICOS and highly expressed the VI-GEM (Extended Data Fig. 3c). These cells were broadly annotated as CD8^+^ T cells in the original study^13^ but were mixed together with other cell states based on scRNA-seq alone, indicating the importance of utilizing multimodal data for identifying rare cell populations that may otherwise be overlooked. Together, the multimodal analysis identified CD8^+^ T cell subpopulations and molecular signatures that were induced after vaccination and were reproducible across donors and studies.

### ASAP-seq identifies enhancers in vaccine-induced cells

**Fig. 2:**
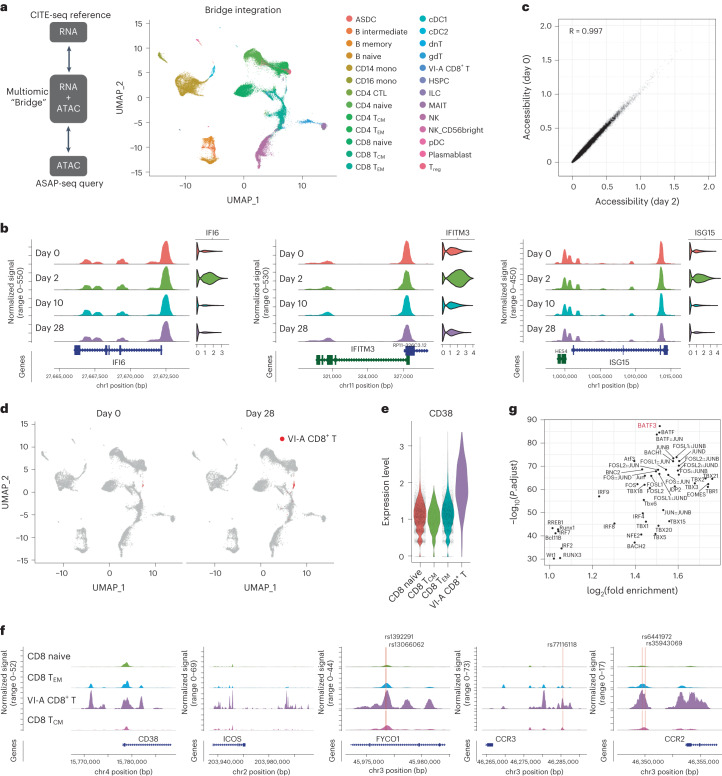
Cell-type-specific chromatin accessibility dynamics in response to vaccination. **a**, Bridge integration-based mapping of scATAC-seq data from Fig. 1a onto the CITE-seq dataset from Fig. 1b. PBMCs were collected as in Fig. 1a. Cells are colored by reference-derived annotation. **b**, Coverage plots indicating chromatin accessibility around *IFI6*, *IFITM3* and *ISG15* in CD14^+^ monocytes across day 0, day 2, day 10 and day 28. Corresponding gene expression for each cell population, from the CITE-seq dataset, is shown on the right. **c**, Scatter plot measuring the correlation between day 0 and day 2 pseudobulk chromatin accessibility of CD14^+^ monocytes. Each point corresponds to a called scATAC-seq peak. **d**, UMAP visualization of scATAC-seq data on day 0 and day 28 after bridge integration. VI-A CD8^+^ T cells are highlighted in red. **e**, Expression of CD38 protein in VI-A CD8^+^ T cells, CD8^+^ naive, CD8^+^ T_CM_ and CD8^+^ T_EM_ cells. **f**, Chromatin accessibility patterns around *CD38*, *ICOS*, *FYCO1*, *CCR3* and *CCR2* gene loci on day 28 in VI-A CD8^+^ T cells, CD8^+^ naive, CD8^+^ T_CM_ and CD8^+^ T_EM_ cells. Single-nucleotide polymorphism (SNP) sites are annotated as yellow lines. **g**, Motif-based overrepresentation analysis of transcription factor binding sites in the top 1,000 peaks with differentially enriched accessibility in the VI-A CD8^+^ T cells.

**Extended Data Fig. 4:**
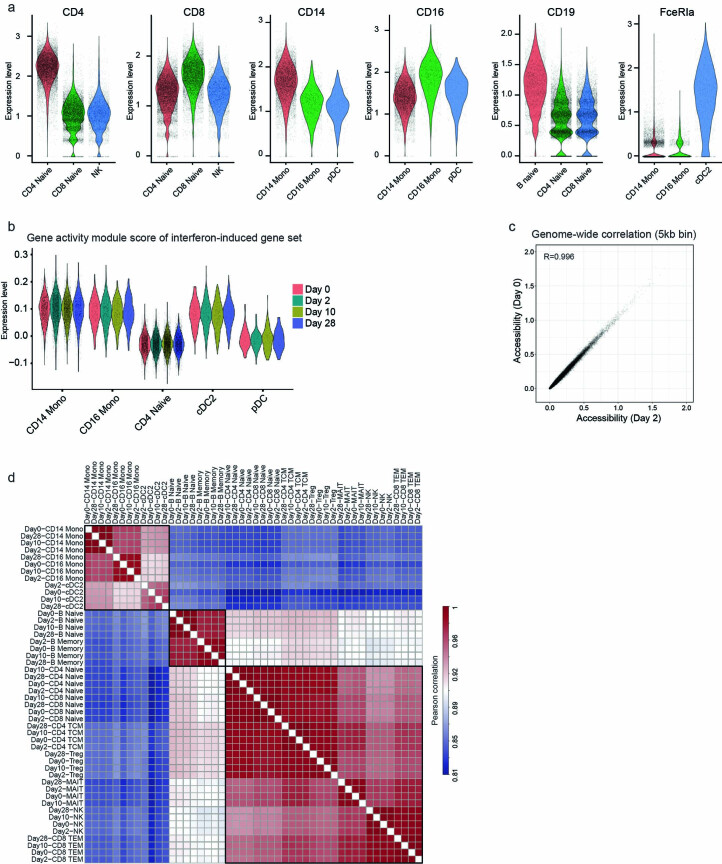
Investigating chromatin accessibility via ASAP-seq dataset. **a**, Violin plots showing the expression of canonical surface proteins in the ASAP-seq dataset. Cells are grouped by bridge integration-derived labels. Proteins visualized include markers of CD4^+^ and CD8^+^ T cells, CD14^+^ and CD16^+^ monocytes, B cells and cDC2 cells. **b**, Violin plots showing the gene ‘activity scores’, which are derived from scATAC-seq data, of the interferon-induced gene set shown in Fig. 1c. **c**, Scatter plot showing the correlation between pseudobulk chromatin accessibility of CD14^+^ monocytes from day 0 and day 2 samples. Each point corresponds to a 5KB genomic bin. **d**, Correlation matrix showing Pearson correlation coefficients (all peaks) between two specified samples of chromatin accessibility.

Although transcriptomic measurements are rich descriptors of a cell’s current state and molecular output, ATAC-seq profiles are uniquely suited for identifying enhancers that exhibit heterogeneous activity and regulators that establish and maintain cellular state. We collected ATAC-seq profiles from the same biological samples as the CITE-seq data but from different cell aliquots. Given the challenges in identifying and annotating high-resolution cellular states from scATAC-seq profiles^23,24^, we aimed to integrate chromatin accessibility profiles with the CITE-seq measurements. To integrate datasets across modalities, we applied a ‘bridge integration’ approach, which can map scATAC-seq query datasets onto scRNA-seq references using a publicly available ‘10x Multiome’ dataset as a bridge^25^. Applying this workflow (Methods), we annotated chromatin accessibility profiles in the ASAP-seq datasets by transferring labels from the CITE-seq reference (Fig. 2a). We validated the inferred annotations using cell surface protein data that was simultaneously generated during ASAP-seq (Extended Data Fig. 4a). For example, predicted monocytes were uniquely enriched for expression of CD14, predicted B cells expressed CD19, predicted dendritic cells upregulated FCER1A, and predicted CD8^+^ T and CD4^+^ T cells expressed CD8 or CD4 surface markers (Extended Data Fig. 4a).

**Extended Data Fig. 5:**
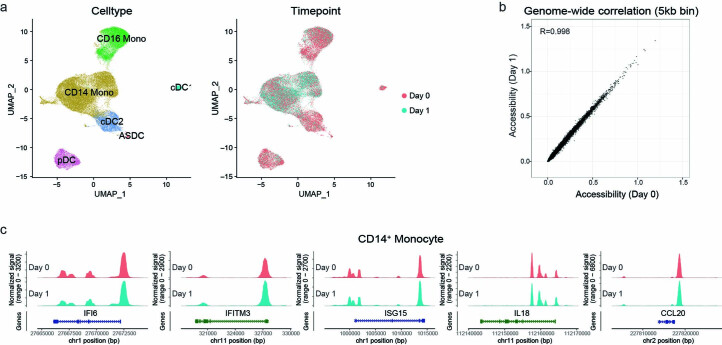
Exploring chromatin accessibility shifts in myeloid cells following TIV vaccination. **a**, UMAP visualization of the scATAC-seq profiles of myeloid cells from a trivalent inactivated seasonal influenza vaccine (TIV) study. Cells are colored by annotations (left) or timepoints (right). **b**, Scatter plot showing the correlation comparing pseudobulk chromatin accessibility of CD14^+^ monocytes in the TIV dataset between day 0 and day 1. Each point corresponds to a 5KB genomic bin. **c**, Visualization of open chromatin accessibility at representative loci on day 0 and day 1 in CD14^+^ monocytes from the TIV study.

Examination of the accessibility changes in the innate immune response did not indicate marked remodeling of chromatin accessibility at interferon-responsive genes in myeloid cells, despite their transcriptomic upregulation (Fig. 2b,c and Extended Data Fig. 4b,c). In a genome-wide analysis, which included both proximal and distal regions (Methods), the chromatin accessibility profiles of CD14^+^ monocytes were highly concordant before and after vaccination (*R* = 0.997; Fig. 2c). Although we did detect a small number of peaks (*n* = 106) that were differentially accessible across timepoints, these changes reflected minor quantitative fluctuations, as opposed to the opening or closing of regulatory regions (Fig. 2c and Extended Data Fig. 4c). We observed similar findings for major subsets of B, T and NK cells (Extended Data Fig. 4d and Supplementary Table 4). These results suggested that the epigenetic landscape required to drive the transcriptional innate immune response was already established before vaccination, enabling the cells to quickly respond to external stimuli^13,26,27^. We also identified nearly identical patterns when we reanalyzed a published dataset^28^ of chromatin accessibility profiles before and after influenza vaccination (Extended Data Fig. 5; *R* = 0.998). These results suggested that chromatin accessibility patterns in myeloid cells exhibited only minor fluctuations during the initial innate immune response, and highlighted how pre-established cell-type-specific differences in accessibility correlated with future functional potential.

**Extended Data Fig. 6:**
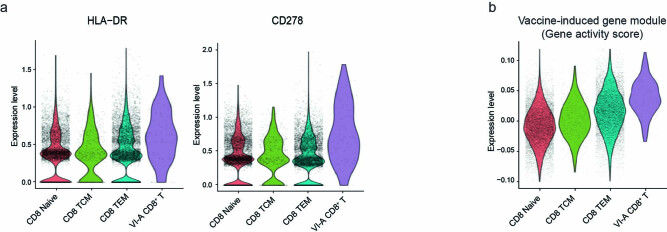
Validation of VI-A CD8^+^ T Cells predicted by bridge integration workflow. **a**, Violin plots showing the protein expression of HLA-DR and CD278 (ICOS) in the VI-A CD8^+^ T cells identified in the ASAP-seq dataset. Cells are grouped by their bridge integration-derived labels. **b**, Violin plots showing the module score of VI-GEM in the ASAP-seq dataset. The module score is calculated based on gene activity scores, which are derived from scATAC-seq data.

The bridge integration workflow also annotated VI-A CD8^+^ T cells in the ASAP-seq datasets (Fig. 2a). These cells increased sharply in frequency after boost vaccination at day 28 (Fig. 2d), and upregulated the expression of CD38, HLA-DR and ICOS (Fig. 2e and Extended Data Fig. 6a). Because the cell surface protein measurements were not considered during the bridge integration procedure^25^, their consistency with the CITE-seq dataset represented an independent validation of our annotations. Moreover, VI-A CD8^+^ T cells exhibited elevated gene ‘activities’ for the VI-GEM identified by CITE-seq (Methods and Extended Data Fig. 6b). We did not observe a second population of proliferating cells in the ATAC-seq data (Fig. 2a), likely due to only subtle differences in chromatin accessibility that can accompany cell cycle changes^29^.

Next, we explored unique features of the chromatin landscape in VI-A CD8^+^ T cells. We identified 2,678 peaks exhibiting differential accessibility in VI-A CD8^+^ T cells (Supplementary Table 5) compared to all other CD8^+^ T cell subsets (Methods). These peaks included putative enhancer elements upstream of the CD38 and ICOS loci (Fig. 2f). Globally, 1,350 peaks were either proximally located (within 20 kb), or linked through publicly available promoter-capture Hi-C data^30^ from CD8^+^ T cells, to genes that were upregulated in vaccine-responsive CD8^+^ T cells. However, among the 864 peaks that could confidently be assigned a target gene based on Hi-C data, 444 peaks did not exhibit similar transcriptional differences, suggesting the pre-establishment of a chromatin landscape that would enable the downstream function of these cells. Enhancers specific to vaccine-specific cells harbored 13 SNPs previously reported to be highly associated (*P* value > 5 × 10^−8^) with COVID susceptibility^31^, including within elements adjacent to *FYCO1*, *CCR3*, *CCR2* and *IFNAR2* (Fig. 2f).

Next we asked if the ASAP-seq data could identify specific regulators required for the development and maintenance of VI-A CD8^+^ T cells. To accomplish this, we searched for transcription factor binding motifs that were overrepresented in specific peak subsets. We found that the motif for the transcriptional regulator BATF3, which is required for the specific development of CD8^+^ memory T cells^32^, exhibited the strongest association with increased accessibility in VI-A CD8^+^ T cells (Fig. 2g). Because BATF3 has been characterized as a critical regulator of DC development^33,34^, these observations suggested that VI-A CD8^+^ T cells contributed to CD8^+^ T memory responses.

### VI-GEM expression correlates with clonal expansion

**Extended Data Fig. 7:**
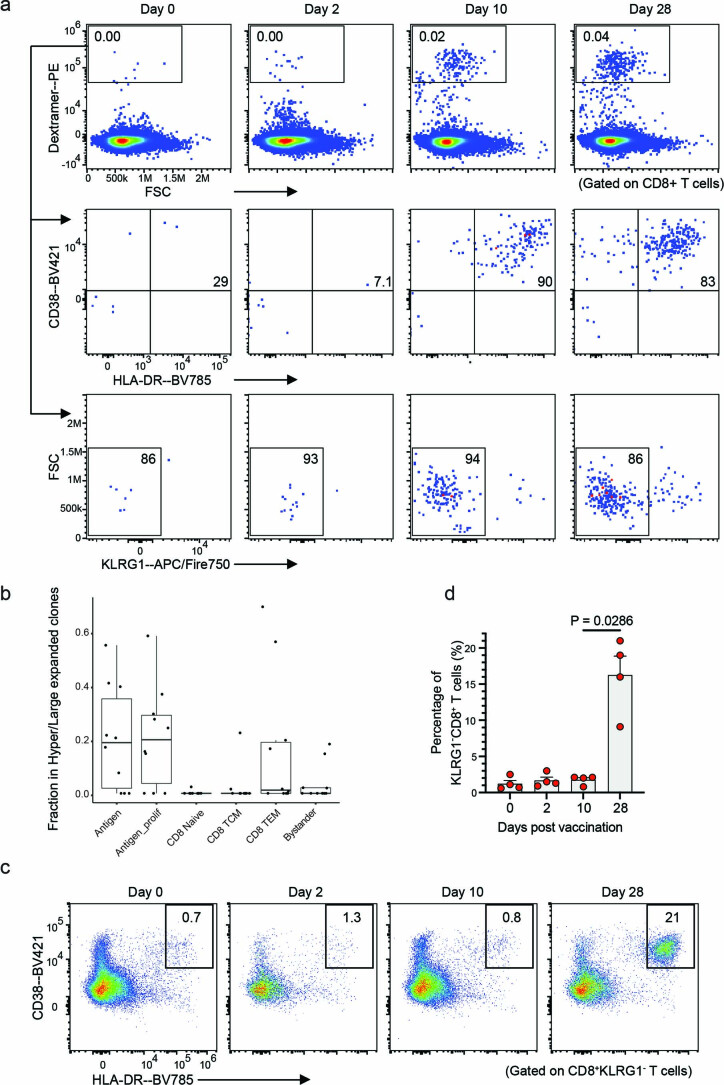
Flow cytometry evaluation of Dex^+^CD8^+^ T Cells. **a**, Flow cytometry data generated during validation of individual dextramer reagents, with the progressive emergence of cells in the Dex^+^ gate across timepoints for a single donor. CD8^+^ cells were used as input. Middle and bottom row show CD38, HLA-DR, and KLRG1 abundance from the parent gate of Dex^+^CD8^+^ T cells. **b**, Boxplots indicate the fraction of cells harboring a hyper- or large-expanded TCR clone in each cluster across n = 10 samples. Each dot represents one biological sample. Box center lines, bounds of boxes and whiskers indicate median, first and third quartiles and minima and maxima within a 1.5× interquartile range (IQR), respectively. **c**, Exemplary flow cytometry plots indicate the percentage of cells in CD38^+^HLA-DR^+^ gate of a single donor, from a parent gate of KLRG1^−^CD8^+^ cells. **d**, Bar graph shows the percentage of CD38^+^HLA-DR^+^ cells in each donor, as a fraction of the KLRG1^−^CD8^+^ gate exemplified in c. Data represents n = 4 donors with variable HLA haplotypes, presented as mean ± s.e.m.; p-value is calculated using unpaired Mann-Whitney U test. PE, phycoerythrin.

Although our previous analyses identified and characterized CD8^+^ T cell populations that were induced in response to vaccination, our initial dataset could not establish if these subgroups were mounting antigen-specific responses. To address this, we used dual DNA-oligo-tagged and fluorochrome-tagged peptide-class I MHC multimers^35^, constructed off a dextran backbone (‘dextramers’). We selected reagents designed to bind TCRs specific for immunodominant SARS-CoV-2 spike peptides, which enable direct ex vivo detection of antigen-specific T cells by either sequencing or cytometry. We selected eight total donors carrying HLA-A*02:01 or HLA-B*07:02 alleles and assayed for dextramer-positive (Dex^+^) cells initially by flow cytometry. We validated five such dextramer reagents to include in our panel (each targeting a separate peptide epitope), by demonstrating a robust and specific appearance of Dex^+^CD8^+^ T cells after vaccination (Extended Data Fig. 7a). To explore the heterogeneity within responding cells, we performed additional single-cell profiling using Expanded CRISPR-compatible CITE-seq (ECCITE-seq), which enables joint profiling of immunophenotypes, 5’-end transcriptomes and immune repertoires^36^. We included the dextramer panel to detect the T cells specific for SARS-CoV-2 spike protein. To enhance recovery of rare cell states, we restricted the analysis to day 28 PBMCs and performed pre-enrichment steps through flow cytometric labeling and sorting, with 25% representing all CD8^+^ T cells, and 75% additionally enriched for CD38 expression and/or dextramer binding (Methods). Our final dataset consisted of 31,396 single cells.

**Fig. 3:**
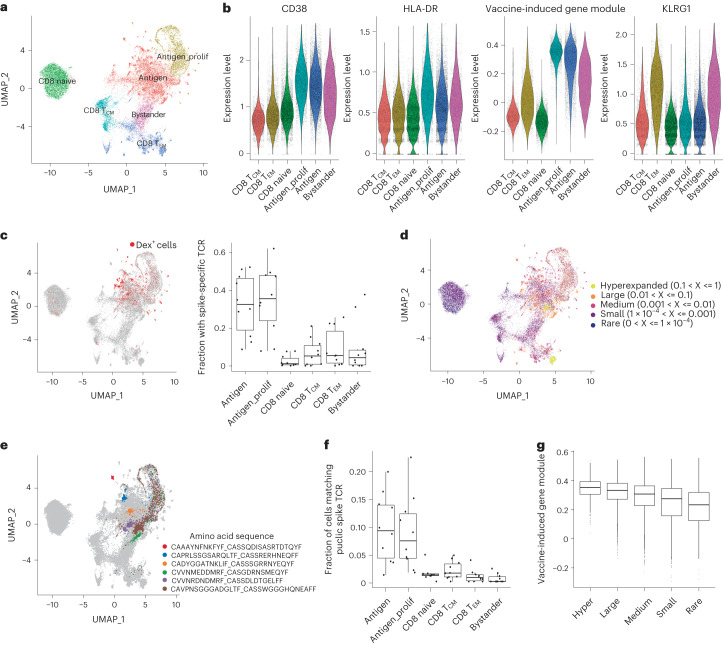
Antigen-specific clonal expansion of vaccine-induced CD8^+^ T cells. **a**, UMAP visualizations of 31,396 single cells profiled with ECCITE-seq and clustered based on weighted combination of RNA, protein and T cell receptor information. **b**, Violin plots for CD38, HLA-DR and KLRG1 protein expression, and the expression of VI-GEM in antigen, antigen_prolif, bystander CD8^+^ T cells, CD8^+^ naive, CD8^+^ T_CM_ and CD8^+^ T_EM_ cells. **c**, UMAP visualization from **a**, with Dex^+^ cells highlighted in red (left) and the fraction of cells harboring spike-specific TCR in each cluster (right). A TCR clone is considered spike-specific when at least one cell of the clone is Dex^+^. Boxplot shows variation across *n* = 10 samples. **d**, UMAP visualization from **a**. Cells are colored by the expansion index of their associated clonotype based on TCR sequence information. **e**, UMAP visualization from **a**. Cells representing the six most abundant spike-specific clones are highlighted. **f**, Boxplots showing the fraction of cells harboring TCR matching SARS-CoV-2 spike antigens in public databases (*n* = 10). **g**, Boxplots showing the single-cell expression of the VI-GEM in antigen and antigen_prolif CD8^+^ T cells. Cells are grouped by labels in panel d. Hyper: *n* = 815; large: *n* = 4058; medium: *n* = 5531; small: *n* = 4709; rare, *n* = 506. For the boxplots in panels c, f and g, the central line represents the median, whereas the upper and lower limits of the boxes correspond to the upper and lower quartiles, respectively. The whiskers extend to 1.5 times the interquartile range (IQR).

Clustering and visualization of cells using WNN analysis based on three modalities (protein, transcriptome and T cell receptor sequence) allowed us to define cellular state based on all data types (Methods). We identified six cell clusters, including naïve CD8^+^ T and CD8^+^ central memory T cell subsets (Fig. 3a). In addition, matching the CITE-seq dataset, we observed both cycling (‘antigen_prolif’) and non-cycling (‘antigen’) subsets of CD8^+^ T cells that exhibited elevated expression of VI-GEM, as well as CD38 and HLA-DR surface proteins (Fig. 3a,b). These clusters were strongly enriched for Dex^+^ cells (Fig. 3c) as well as large and expanded cell clones (Fig. 3d and Extended Data Fig. 7b). We also found extensive TCR sharing between the antigen_prolif and antigen groups (Fig. 3e).

Our enrichment strategy enabled us to explore further sources of cellular heterogeneity amongst CD38^+^CD8^+^ T cells (Fig. 3a,b). We found that a subset of CD38^+^CD8^+^ T cells uniquely expressed the inhibitory receptor KLRG1 (Fig. 3a,b). In contrast to the antigen and antigen_prolif clusters, CD38^+^KLRG1^+^CD8^+^ T cells were not enriched in Dex^+^ cells (Fig. 3c), did not show evidence of expanded clonality and did not show enriched overlap with TCRs on antigen-specific cells (Fig. 3d,e). To address the possibility that the CD38^+^KLRG1^+^CD8^+^ T cells harbored TCRs not recognized by the dextramer panel, we examined a large external database of TCRβ sequences^37,38^ specific for SARS-CoV-2 spike protein (Methods). Unlike CD38^+^KLRG1^−^CD8^+^ T cells, which showed marked overlap with SARS-CoV-2 TCRs, CD38^+^KLRG1^+^CD8^+^ T cells had minimal overlap with these documented clonotypes (Fig. 3f and Methods). They also exhibited weaker expression of VI-GEM (Fig. 3b), suggesting that CD38^+^KLRG1^+^CD8^+^ T cells may represent cells expressing TCR with weak affinity for spike protein antigens, or alternatively, represent TCR-independent ‘bystander’ responses, such as those previously described within the microenvironments of tumors and other pathogens^39,40^.

Multiparameter flow cytometry on the Dex^+^ gate indicated that these cells were KLRG1^−^ in addition to being CD38^+^HLA-DR^+^ (Extended Data Fig. 7a), consistent with our initial CITE-seq. As these three markers represented prominent features from the CITE-seq and ECCITE-seq experiments, we gated for this population by flow cytometry within all CD8^+^ T cells and compared across timepoints (Extended Data Fig. 7c,d). We observed a marked induction of this population on day 28 (Extended Data Fig. 7d), an observation agnostic to the donor’s HLA haplotype or immunopeptidome. We conclude that CD38^+^HLA-DR^+^KLRG1^−^CD8^+^ T cells were the most highly enriched for antigen-specific CD8^+^ T cells.

The rate of clonal expansion of antigen-specific T cells is an indicator of the strength of the immune response^41^. When we searched for gene expression patterns that were correlated with clonal size, even among antigen-specific cells, we found that the expression of VI-GEM was upregulated in antigen and antigen_prolif subsets (Fig. 3b) and that the module score exhibited a dose-dependent relationship with clonal size (Fig. 3g). Of note, the VI-GEM was shared in both antigen_prolif and antigen groups (Fig. 3b), and therefore did not include proliferation-dependent genes that would be expected to correlate with clonal size. Instead, expression of VI-GEM likely reflected the signal strength of the original TCR-peptide interaction, an essential parameter which regulates the magnitude of clonal expansion and immune response^42,43^. Taken together, the multimodal ECCITE-seq dataset verified the spike-specific nature of vaccine-induced CD8^+^ T cells, nominated specific biomarkers that subdivided heterogeneous activated populations, and identified specific gene modules and surface markers that could be used to predict clonal dynamics, even in the absence of HLA haplotype and immune repertoire information.

### CD8^+^ T cell responses predict COVID-19 progression

**Fig. 4:**
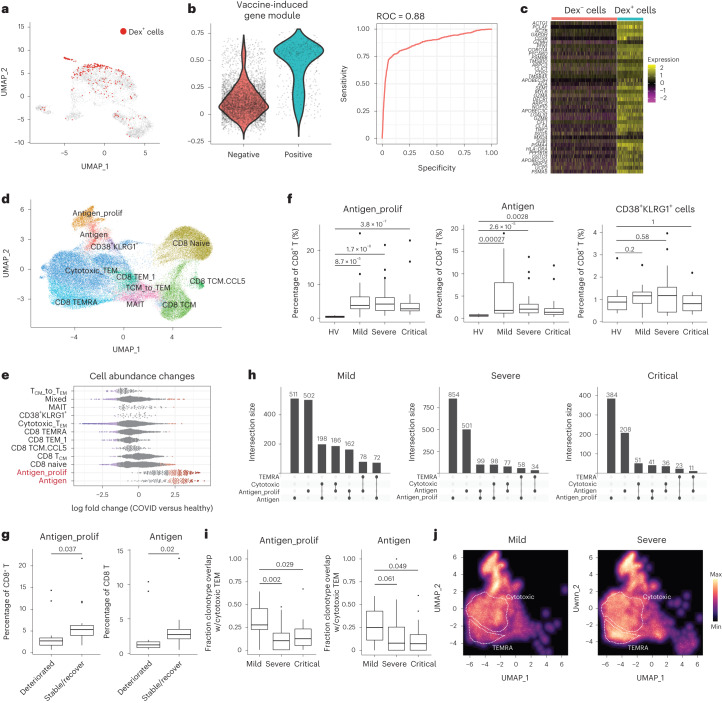
Inferred spike-specific T cells in SARS-CoV-2-infected samples. **a**, UMAP visualization from Adamo et al.^44^, representing 6,070 CD8^+^ T cells collected during acute COVID-19 disease. Dex^+^ cells are highlighted in red. **b**, Violin plots showing distribution of expression of VI-GEM in Dex^−^ and Dex^+^ cells (left) and receiver operating characteristic (ROC) curve assessing the ability of the expression of VI-GEM to correctly predict Dex^+^ cells (right). **c**, Expression of top 40 genes of VI-GEM in Dex^−^ and Dex^+^ cells. For visualization purposes, a randomly selected subset of Dex^−^ cells are presented. **d**, WNN UMAP visualization of 65,889 single cells from the COMBAT dataset. WNN performed based on RNA and protein modalities. **e**, Milo analysis of differential abundance changes in healthy versus SARS-CoV-2-infected groups, as in Fig. 1e**. f**, Boxplots showing donor fraction of antigen, antigen_proflif, and CD38^+^KLRG1^+^ cells amongst all CD8^+^ T cells, grouped by disease state. *n* = 71 donors (HV: *n* = 10; mild: *n* = 17; severe: *n* = 28; critical: *n* = 16), and *P* values from two-tailed Wilcoxon rank-sum test. **g**, Boxplots showing donor fraction of antigen, antigen_prolif, and CD38^+^KLRG1^+^ cells amongst all CD8^+^ T cells in patients exhibiting severe symptoms grouped by their clinical outcome. Deteriorated, *n* = 12; stable/recover, *n* = 16. **h**, UpSet plot visualizing the overlap of TCR clonotype across antigen, antigen_prolif, cytotoxic and T_EMRA_ CD8^+^ T cells. **i**, Fraction of TCR clonotypes identified in either antigen cells (right) or antigen_prolif cells (left) that were also observed in cytotoxic T_EM_ cells. Boxplots show variation across 61 diseased donors (mild: *n* = 17; severe: *n* = 28; critical: *n* = 16). *P* values were determined by two-tailed Wilcoxon rank-sum test. **j**, Density plots showing the abundance distribution of all CD8^+^ T cells harboring the same TCR clonotypes identified in antigen and antigen_prolif CD8^+^ T cells. For the boxplots in panels f, g and i, the central line represents the median, whereas the upper and lower limits of the boxes correspond to the upper and lower quartiles, respectively. The whiskers extend to 1.5 times the IQR. HV, healthy volunteers; T_EMRA_, effector memory T cells re-expressing CD45RA.

To ask if VI-GEM was conserved in samples from patients infected with SARS-CoV-2, we first examined a published dataset that used a SARS-CoV-2 dextramer panel to identify long-lived memory CD8^+^ T cells during acute SARS-CoV-2 infection^44^. Although unsupervised clustering of the published scRNA-seq data^44^ did not clearly identify Dex^+^-enriched CD8^+^ T cell clusters (Fig. 4a), we found that the expression of the VI-GEM had high predictive power (receiver operating characteristic = 0.88) to accurately predict Dex^+^ cells (Fig. 4b). We observed that the VI-GEM originally identified in vaccinated datasets was highly conserved in the Dex^+^ cells in the dataset^44^ of SARS-CoV-2-infected samples (Fig. 4c).

**Extended Data Fig. 8:**
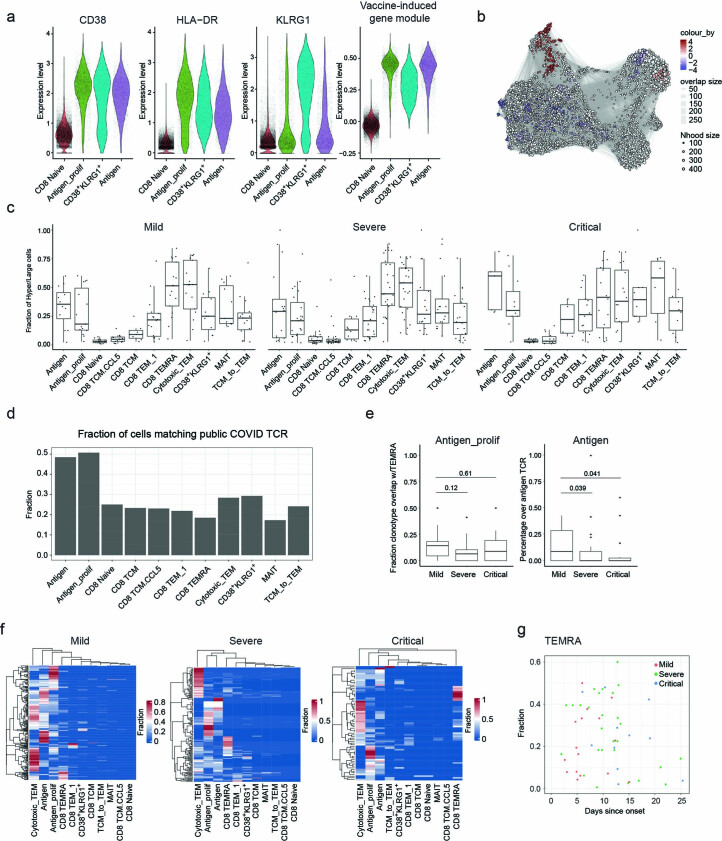
Assessing the differential abundance of CD38^+^KLRG1^-^CD8^+^ T cells in SARS-CoV-2 infected samples. **a**, Violin plots showing the protein expression of CD38 and HLA-DR, along with the signature gene module score for the vaccine-induced cells, in the COMBAT dataset. **b**, Milo analysis of differential abundance changes between healthy and SARS-CoV-2 infected CD8^+^ T cells groups from the COMBAT dataset. UMAP visualization of the Milo differential abundance testing results. Each node represents a neighborhood. The size of nodes is proportional to the number of cells in the neighborhood. Neighborhoods are colored by their log fold changes for SARS-CoV-2 infected versus healthy groups. Only neighborhoods showing significant enrichment (SpatialFDR < 0.1 and logFC > 2) are colored. **c**, Boxplots showing the fraction of cells harboring a hyper or large expanded TCR clone within each cluster. Each dot represents one biological sample. Mild: n = 17; Severe: n = 28; Critical: n = 16. **d**, Barplot showing the fraction of cells within each cluster harboring TCR matching SARS-CoV-2 antigens in public databases. **e**, Fraction of TCR clonotypes identified in either antigen cells (right) or antigen_prolif cells (left), that are also identified in T_EMRA_ cells. Boxplots show variation across 61 diseased donors. Mild: n = 17; Severe: n = 28; Critical: n = 16. P values were determined by two-tailed Wilcoxon rank-sum test. **f**, Heatmaps show the distribution of cells harboring expanded antigen-specific TCR sequences among all cell states. Each row corresponds to one expanded clone, clones that are shared between molecular states will exhibit a positive fraction in multiple columns. **g**, Scatter plot showing the lack of a potentially confounding correlation between the fraction of CD8 T cells in the T_EMRA_ state, and the sample collection time since onset. Each dot represents one donor and is colored by disease state. For the boxplots in c and e, the center line indicates the median, box limits represent the upper and lower quartiles and whiskers indicate 1.5 times the IQR.

SARS-CoV-2-specific adaptive immune responses are associated with milder disease^45^. To test whether the abundance of antigen-specific CD8^+^ T cells correlated with disease phenotype and progression, we reanalyzed a large ECCITE-seq dataset (transcriptome, surface protein and TCR) from the COVID-19 multi-omics blood atlas (COMBAT)^46^, which contains 65,889 CD8^+^ T cells prospectively collected from 10 healthy controls and 61 COVID-19 patients at the time of admission to inpatient hospital care, and who subsequently manifested mild, severe or critical disease^46^. Applying the WNN integrative analysis pipeline, we identified analogous clusters enriched in the expression of VI-GEM as well as expression of CD38 and HLA-DR (Fig. 4d and Extended Data Fig. 8a), suggesting these populations were specific to SARS-CoV-2 antigens. Abundances of both antigen_prolif and antigen clusters were sharply elevated in all SARS-CoV-2-infected samples compared to healthy controls (Fig. 4e and Extended Data Fig. 8b). We also identified CD38^+^KLRG1^−^ and CD38^+^KLRG1^+^ CD8^+^ T cells (Fig. 4d and Extended Data Fig. 8a). In terms of abundance, CD38^+^KLRG1^−^CD8^+^ T cells (both ‘antigen’ and ‘antigen_prolif’ clusters), but not the CD38^+^KLRG1^+^CD8^+^ T cell clusters, were associated with the severity and trajectory of COVID-19 (Fig. 4f). The relative abundance of antigen_prolif and antigen cells was sharply increased in SARS-CoV-2-infected samples compared to healthy controls (Fig. 4f) but were progressively lower across the spectrum of mild to critical patients (Fig. 4f). Moreover, we found that patients who exhibited severe disease at the time of sample collection but later recovered (*n* = 16) exhibited an increased relative abundance of CD38^+^KLRG1^-^CD8^+^ T cells, compared to patients with severe disease who further deteriorated (Fig. 4g). This suggests that patients who did not mount effective cellular immune responses were more likely to succumb to critical COVID-19.

We next explored the relationship between immune repertoire sequences and molecular state, which were simultaneously measured in the COMBAT dataset. As expected, antigen and antigen-prolif clusters were enriched for cells participating in either large- or hyper-expanded clones (Extended Data Fig. 8c). Only CD38^+^KLRG1^-^CD8^+^ T cells exhibited enriched overlap with a public database of SARS-CoV-2-specific TCR sequences (Extended Data Fig. 8d), indicating that in both vaccination and infection, KLRG1 expression demarcated heterogeneous immune responses amongst activated and responding CD8^+^ T cells. Lastly, we observed extensive TCR sharing between different CD8^+^ T cell subsets (Fig. 4h), indicating evidence for lineage-specific differentiation trajectories. Exploring the TCR clonotype overlap between antigen-specific CD38^+^KLRG1^−^CD8^+^ T cells with CD8^+^ T cell subsets, we found the most substantial overlap was with highly cytotoxic CD127^−^CD45RA^−^CD27^−^CD8^+^ T_EM_ cell subsets, and lower overlap with CD127^−^CD45RA^+^CD27^−^CD8^+^ T_EMRA_ cells (Fig. 4h). The molecular state of differentiated T cells sharing CD38^+^KLRG1^−^ TCRs also varied as a function of disease severity (Fig. 4i). Nearly 25% of TCR sequences observed in predicted antigen-specific subsets exhibited clonal overlap with cytotoxic subsets of CD127^−^CD45RA^−^CD27^−^CD8^+^ T_EM_ cells in COVID-19 patients with mild SARS-CoV-2 infection, but this percentage was sharply reduced in severe (median of 7.74% for antigen cells, 10% for antigen_prolif cells) or critical COVID-19 patients (median of 7.14% for antigen cells, 12.1% for antigen_prolif) (Fig. 4i). This level of clonal overlap was not observed in CD127^−^CD45RA^+^CD27^−^ CD8^+^ T_EMRA_ cells (Extended Data Fig. 8e), and as a result, the distribution of cells harboring expanded antigen-specific TCR sequences was skewed toward a T_EMRA_ phenotype in these samples (Fig. 4j and Extended Data Fig. 8f). These findings were not driven by potential correlations between disease severity and time since onset (Extended Data Fig. 8g). These results showed that the abundance of CD38^+^HLA-DR^+^KLRG1^−^ CD8^+^ T cells and their molecular differentiation outcomes during SARS-CoV-2 infection were predictive of disease severity and clinical progression.

## Discussion

Although the protein biomarkers CD38 and HLA-DR identified here have been previously used to characterize antigen-specific CD8^+^ T cells in flow cytometry assays^5,44^, our unsupervised single-cell profiling strategy identified additional heterogeneity within this important subset. In addition to identifying both cycling and noncycling antigen-specific CD8^+^ T cells, we observed heterogeneity in the expression of KLRG1 within this group and found that KLRG1^−^ subpopulations were most likely to contain highly clonal CD8^+^ T cells that exhibited binding to spike-specific dextramer reagents. Although KLRG1 is a highly cytotoxic molecule, within antigen-specific T cells, its expression has been linked to a short-lived phenotype^47–49^. Our results suggested that KLRG1 distinguished cells with distinct antigen specificities, which likely contributed to downstream differences in their phenotype and persistence.

Using molecular signatures from vaccinated samples, we annotated antigen-specific CD8^+^ T cells in additional published datasets^13^, including samples from patients with COVID-19 (refs. ^44,46^). In these samples, we also leveraged immune repertoire information to link antigen-specific CD8^+^ T memory precursor cells with their differentiated progeny. We found that disease severity and outcome correlated not only with the abundance of the CD8^+^ T memory precursor cells but also with the molecular state of their descendants, and in particular, we found that donors who manifested extensive TCR sharing between memory CD8^+^ T precursor cells and cytotoxic CD8^+^ T cell progeny were associated with a milder clinical course. These results exemplify a potential mechanism by which cellular immunity may play an important role in resolving viral infection.

Although our study is rooted in analyzing mRNA vaccination and coronavirus disease, the antigen-specific CD8^+^ T cell subpopulations we uncover are likely to represent features of human immune responses more broadly. For example, a study identified a subpopulation of circulating CD8^+^ T cells, similarly enriched for expression of CD38 and HLA-DR, whose abundance within the primary tumor and within PBMCs changed after a 3-week course of checkpoint blockade therapy^50^. In a separate context, the study also identified heterogeneity in the expression of KLRG1 and found that the specific abundance of PD1^+^KLRG1^−^ cells within that subset positively correlated with optimal induction of tumor antigen-specific T cells and overall treatment outcome^50^. Taken together, these results demonstrated the potential for monitoring antigen-specific T cells to inform our understanding of disease and treatment trajectories.

## Methods

### Ethics statement

All research complied with relevant ethical regulations, as outlined by New York University’s Institutional Review Board (across protocols 18-02035, 18-02037 and 12-01137).

### Human participants and PBMC collection

PBMCs were collected from observational studies of adults (Supplementary Table 1) who were receiving BNT162b2 vaccination and willing to participate, excluding individuals with severe anemia or inability to comply with procedures. The specific subset of donors included 12 females and 4 males of variable racial and ethnic background, aged 17 to 58 years (Supplementary Table 1). All groups were provided with written consent for enrollment with approval from the New York University Institutional Review Board (across protocols 18-02035, 18-02037 and 12-01137). Participants had blood drawn at a baseline beforehand (day 0), on day 2 and day 10 after prime vaccination, as well as day 28 (7 days after boost vaccination at day 21), with 1-2 days flexibility in scheduling. Sample size calculations were not performed before the start of these nonrandomized, non-interventional studies, and outlier analyses were not performed.

Venous blood was collected by standard phlebotomy (total volumes ranging 40–80 ml). Within 5 h of room-temperature transport from an outpatient clinic, PBMCs were isolated from heparin vacutainers (BD Biosciences), followed by processing using SepMate (STEM-CELL Technologies), Ficoll-Paque Premium with density 1.077 (Cytiva) and Hank’s balanced salt solution (ATCC), in accordance with manufacturers’ recommendations. Aliquots of 1 ml were slowly frozen overnight within Corning CoolCell containers placed in −80 °C freezers, with cells suspended in complete media (RPMI 1640 supplemented with 40% fetal bovine serum) along with 10% DMSO, and after 2 days, all vials were transferred to liquid nitrogen.

### Flow cytometry and sorting

For initial CITE-seq and ASAP-seq experiments, PBMCs from all timepoints (days 0, 2, 10 and 28) across 3 donors (12 specimens in total) were simultaneously thawed and promptly transferred to a 96-well V-bottom plate. This enabled further processing in parallel with multichannel pipettes. The same workflow was repeated with 3 additional donors, to generate the aggregate data in Figs. 1 and 2. Each aliquot of 1–3 million frozen PBMCs was thawed into 10 ml complete media, centrifuged at 300 RCF for 10 min at 4 °C and resuspended in 200 µl conventional cytometry buffer (PBS with 4% fetal bovine serum), DAPI and 2 mM EDTA. Samples were passed through a 70-micron filter, and single cells were sorted on a FACSAriaII (BD Biosciences) using a 100-micron nozzle. The instrument operated via FACSDiva software, with post-sort analysis performed on FlowJo 10.8.1 (Tree Star). Gating excluded cellular debris and doublets based on FSC and SSC profiles and excluded dead cells based on DAPI. Cells were collected into 5 ml complete media separately maintained on ice until all sorting concluded, at which point all tubes were simultaneously centrifuged. Individual pellets were resuspended with 100 µl staining buffer (PBS with 2% BSA and 0.01% Tween) along with unique hashing antibodies, followed by incubation on ice for 15 min. Hashed samples were washed three times with 500 µl staining buffer and then pooled together. Viability (greater than 92%) and final cell counts were assessed with trypan blue and Countess II FL automated counter (ThermoFisher).

### CITE-seq library preparation

Workflows for CITE-seq and cell hashing were performed as previously described^14,51^. An aliquot of 300,000 sorted and hashed cells was stained with 173 TotalSeq-A antibody panel (BioLegend, Catalog: 399907. Supplementary Table 2). After incubating on ice for 30 min, cells were washed three times with 1 ml staining buffer to remove excess antibody. Cells were passed through a 40-micron Flowmi filter, resuspended in PBS and ultimately loaded onto four lanes of 10x Genomics Chip G, following manufacturer protocols.

RNA library construction was performed according to the 10x scRNA-seq protocol, whereas the ADT and HTO library constructions were conducted following the CITE-seq protocol (https://citeseq.files.wordpress.com/2019/02/cite-seq_and_hashing_protocol_190213.pdf). During cDNA amplification (Step 2.2a), 0.2 μM of ADT additive primer (5′-CCTTGGCACCCGAGAATTCC-3′) and 0.1 μM HTO additive primer (5′GTGACTGGAGTTCAGACGTGTGCTC-3′) were added to the reaction mixture to enrich antibody tags. During cDNA cleanup (Step 2.3), supernatant containing the antibody tags was saved and further purified with 2x SPRI. The eluate was split into two tubes for ADT and HTO libraries. After cDNA cleanup, additional PCR reactions generated ADT and cell hashing libraries. These reactions were set up with KAPA Hifi Master Mix with the following primers: 10 μM 10x Genomics SI-PCR primer (5′-AATGATACGGCGACCACCGAGATCTACACTCTTTCCCTACACGACGCTC-3′), and 10 μM Illumina TruSeq DNA D7xx primer (5′-CAAGCAGAAGACGGCATACGAGATxxxxxxxxGTGACTGGAGTTCAGACGTGTGC-3′) for HTO library. 10 μM 10x Genomics SI-PCR primer, and 10 μM TruSeq Small RNA RPIx primer (5′-CAAGCAGAAGACGGCATACGAGxxxxxxxxGTGACTGGAGTTCCTTGGCACCCGAGAATTCCA-3′) for ADT library. The PCR products were purified with 1.6x SPRI.

### scATAC-seq library preparation

ASAP-seq was conducted as previously described^15^, with minor modifications. After staining with cell surface antibodies, cells were fixed in 0.1% formaldehyde for 5 min at room temperature. After washing, the cell pellet was resuspended in 100 µl lysis buffer (10 mM Tris-HCl pH 7.4, 10 mM NaCl, 3 mM MgCl_2_, 0.1% Tween-20, 0.1% Nonidet-P40 substitute (IGEPAL) and 1% BSA) and kept on ice for 5 min. The permeabilized cells were then resuspended with 1× Diluted Nuclei Buffer (10x Genomics) to a concentration of around 5000 cells/µL. 10 µL transposition mix (3 µl 10x ATAC Buffer B and 7 µl 10x ATAC Enzyme) was mixed with 5 µl sample and incubated for 1 h at 37 °C. 0.5 μM bridge oligo A (TCGTCGGCAGCGTCAGATGTGTATAAGAGACAGNNNNNNNNNVTTTTTTTTTTTTTTTTTTTTTTTTTTTTTT/3InvdT/) was added to the barcoding mix for proper amplification of antibody tags. The GEM incubation was performed with the following PCR program: 40 °C for 5 min, 72 °C for 5 min, 98 °C for 30 s; 12 cycles of 98 °C for 10 s, 59 °C for 30 s and 72 °C for 1 min; ending with hold at 15 °C. Post-GEM incubation cleanup and library construction were conducted following the ASAP-seq protocol (https://citeseq.files.wordpress.com/2020/09/asap_protocol_20200908.pdf).

### Dextramer validation with spectral flow cytometry

We initially tested a panel of 16 commercially available dextramer reagents (Immudex, catalog: RX19) designed to bind SARS-CoV-2 spike protein MHC class I epitopes^35^ across 7 HLA haplotypes. All reagents were tagged with a unique DNA oligo barcode as well as PE fluorochrome. PBMC aliquots from all four timepoints for each donor were thawed as above and were subsequently resuspended in a cytometry buffer containing 0.1 gram/liter of herring sperm DNA (ThermoFisher) and Human TruStain FcX block (BioLegend). Cells were maintained in this blocking solution for 10 min at room temperature, 1 µl of each test dextramer reagent was subsequently added to each timepoint sample, wells were thoroughly mixed, and the plate was incubated at 4 °C in the dark for 10 min. A separate antibody staining panel was also prepared in cytometry buffer, containing CD8a at 1:250 dilution, as well as 1:100 dilutions of CD2, CD4, CD14, CD16, and CD20. This was directly added (100 µl/well) to each well after initial dextramer incubation, wells were mixed, and the plate was returned to darkened 4 °C for 30 min. The plate underwent four rounds of centrifuge at 300 RCF 4 °C followed by wash with cytometry buffer, with final resuspension including DAPI and EDTA, followed by 70-micron filter passage. Samples were analyzed on a Cytek Aurora cytometer (Cytek) via SpectroFlow software (v3.03), with careful precalibration of fluorochrome spectral profiles to maximize accuracy and sensitivity. The gating strategy included FSC, SSC; DAPI-negative; singlets; Dump^−^ (CD14, CD16, CD20); CD2^+^; CD4^−^; CD8^+^; and a final dextramer/PE-positive gate to identify antigen-specific cells. Consistent with previous reports^44^, only a subset of the 16 dextramer reagents exhibited an acceptable minimal non-specific binding at day 0 and day 2 timepoints, along with distinctly increased binding at day 28 timepoint for the same test donor (Extended Data Fig. 7a).

We chose to use five dextramer reagents that met this validation criteria, spanning the HLA-A*0201 and HLA-B*0702 alleles. These were loaded with the following spike (S) glycoprotein-derived immunodominant peptides and tagged with respective DNA barcodes: VLNDILSRL with TTGTACTGAGTAAGC; YLQPRTFLL with CGGTTACAGTCGGTG; RLNEVAKNL with TCCAGGAACCATATG; NLNESLIDL with CGGTGTTAACGCGTT; SPRRARSVA with AGCTACTCGCACCAC. Our experiments also included a negative control reagent harboring the HLA-A*0201 allele loaded with a nonsense peptide (with barcode CAACTAATATGGTTA), as well as a nonsense HLA loaded with a nonsense peptide (with barcode GCAGACTTAGAAGAA). We identified eight donors who stood out in exhibiting sizable antigen-specific T cell populations exclusively from day 28 specimens (binding one or more of the five validated experimental dextramers) and used these samples to enrich for spike-specific CD8^+^ T cells.

### Enrichment of spike-specific CD8^+^ T cells before ECCITE-seq

To facilitate the study of rare populations, we enriched for spike-specific CD8^+^ T cells before performing ECCITE-seq analyses. We aimed to facilitate enrichment while also mitigating the effect of potential biases, including the fact that no dextramer panel can successfully identify all spike-specific cells across all possible clonotypes. We proceeded to sort three populations: all dextramer-bound CD8^+^ T cells (Bin 1), all CD38^+^CD8^+^ T cells (Bin 2) and an unenriched sampling of all CD8^+^ T cells (Bin 3). Given the relatively scarcity of dextramer-positive cells, we enriched for this population first, and then obtained cells from the subsequent bins.

We stained day 28 specimens with an aggregate panel of all 5 dextramers and 2 negative control reagents. A PCR tube was first loaded with 1.4 µl of 100uM d-Biotin (ThermoFisher) diluted in PBS (to minimize non-specific binding). Then, 10 µl each dextramer specificity was sequentially added, the panel was well mixed, and ultimately 8.93 µl of this dextramer panel was added to each well of PBMC (consistent with manufacturer’s recommended concentrations). A similar antibody panel as above (CD14, CD16, CD20, CD2, CD4 and CD8) was added after dextramer, now also including CD38 at 1:100 dilution, as well as individual CITE-seq antibodies targeting CD8 and CD38. Final incubation with dextramers, fluorochrome antibodies, CITE-seq antibodies and hashing antibodies ensued for 30 min at 4 °C in the dark. Subsequent cell preparation followed our prior cytometry protocol, except samples were loaded onto FACSAriaII for sorting. Gating was the same as above, with an additional CD38-high population created off the CD8 parent gate.

Because dextramer-positive CD8^+^ events were the rarest, we collected all possible cells from this gate. Subsequently, we collected cells from the second and third bins. We then mixed all three bins together, at approximately 10% (Bin 1), 65% (Bin 2) and 25% (Bin 3) ratio. This mixed pool was used as input for ECCITE-seq.

### ECCITE-seq library preparation

Sorted cells were centrifuged at 400 RCF for 8 min at 4 °C and then resuspended in staining buffer. TotalSeq-C human cocktail (BioLegend) (BioLegend, Catalog: 399905. Supplementary Table 2) was added for the surface protein staining, on ice for 30 min. After washing three times with 1 ml staining buffer, cells were resuspended in PBS and the cell concentration was adjusted to about 2000 cells/µL. Cells were loaded onto the 10x Chromium Next GEM Chip N, following manufacturer recommendations (Chromium Next GEM Single Cell 5’ HT Reagent Kits v2). During cDNA amplification, 0.2 μM each of ADT (5′-CCTTGGCACCCGAGAATT∗C∗C-3′) and HTO (5′-GTGACTGGAGTTCAGACGTGTGC∗T∗C-3′) were added to the reaction. RNA, HTO, ADT and TCR libraries were constructed as previously described^36^.

### Sequencing

Sequencing libraries were pooled and sequenced on an Illumina Novaseq using sequencing read lengths of 107 bp (read 1), 8 bp (i7 IndexRead), 16 bp (i5 IndexRead) and 107 bp (read 2). bcl2fastq was used to demultiplex raw sequencing data.

### Pre-processing, quantification and quality control of sequencing data

Sequencing data from ADT and HTO libraries were both aligned and quantified with salmon alevin (v1.8.0)^52^. Custom ADT and HTO indices, based on the DNA oligo barcode sequences, were constructed by running ‘salmon index’ command. Single-cell barcode quantification matrices were generated by running ‘salmon alevin’ command with the following parameters:–naiveEqclass,–keepCBFraction 1.0. RNA-sequencing data were aligned to the GRCh38 human reference genome using Cell Ranger (v6.0.0, ‘cellranger count’) with default settings. ATAC sequencing data was aligned to the GRCh38 human reference genome using Cell Ranger ATAC (v2.0.0 ‘cellranger-atac count’) with default settings. TCR sequencing data was aligned to the GRCh38/Ensembl human reference using Cell Ranger (v6.0.0, ‘cellranger vdj’) with default settings.

For QC, we retained cells that passed the following thresholds: For the RNA modality, we retained cells that surpassed 500 UMI, and exhibited <15% of reads mapping to mitochondrial regions. For the ATAC modality, we retained cells exhibiting at least 900 unique fragments per cell. For the ADT and HTO modalities in CITE-seq, we retained cells that surpassed 500 and 40 unique counts per cell, respectively. For the ADT and HTO modalities in ASAP-seq, we retained cells that surpassed 100 and 40 unique counts per cell, respectively. For each experiment, we retained cells that passed the required thresholds for each measured modality (that is, for CITE-seq data, we retained cells that surpassed thresholds for RNA, ADT and HTO modalities). After performing quality control, we identified and removed doublets based on the cell hashing libraries, using the HTODemux function in Seurat^17^ with default parameters.

### Visualization and clustering of CITE-seq data

To perform clustering and annotation of the original CITE-seq dataset (Fig. 1b), we first processed the RNA and ADT modalities separately, performing normalization, dimensional reduction and data integration steps. Subsequently, we performed WNN analysis^17^ to jointly define cellular state based on RNA and protein data.

#### Normalization and dimensional reduction

We first split the CITE-seq data into 24 separate groups based on the combination of donor identity (*n* = 6) and experimental timepoint (*n* = 4). We performed normalization, feature selection and dimensional reduction on each group independently.

For the RNA modality, we performed normalization using sctransform v1 (ref. ^53^), using the SCTransform function in Seurat. This procedure also performs variance stabilization. We performed dimensional reduction using principal-component analysis (PCA), retaining 40 dimensions. For the ADT modality, we performed normalization using the centered log-ratio (CLR) transformation, implemented in Seurat using the NormalizeData function with the arguments: normalization.method = ‘CLR’, margin=2. We centered the values for each feature to have a mean of 0 across all cells but did not scale features to have unit variance, using the ScaleData function in Seurat (arguments: center=TRUE, scale=FALSE). We included all 173 ADT features for downstream analysis. We performed dimensional reduction using PCA, retaining 40 dimensions.

#### Data integration across donors and timepoints

We next applied our ‘anchor-based’ data integration workflow^16^ to integrate datasets produced across donors and timepoints. We performed separate integration analyses on both the RNA and ADT modalities. For the RNA modality, we selected a consensus set of 3,000 variable features across the 24 experimental groups using the SelectIntegrationFeatures command in Seurat, and augmented this list with the set of up-regulated gene expression markers in VI-A and VI-B CD8^+^ T cells. We performed integration as previously described using the ‘reciprocal PCA’ workflow, as implemented using the FindIntegrationAnchors (arguments: dims=1:40, reduction=’rpca’) and IntegrateData (default parameters) functions. This procedure returns a single 40-dimensional space (integrated.rna) that groups together shared cell states across donors and timepoints based on their transcriptomes. For the ADT modality, as also performed integration using the reciprocal PCA workflow, using all features and utilizing 40 dimensions. This procedure returns a single 40-dimensional space (integrated.rna.pca) that groups together shared cell states across donors and timepoints based on their protein data.

#### Data integration across modalities and cluster annotation

To define cell state based on a weighted combination of RNA and ADT modalities, we constructed a WNN graph^17^. We constructed the graph using the FindMultiModalNeighbors (arguments: reduction.list=c(‘integrated.rna.pca’, ‘integrated.adt.pca’), dims.list=c(1:40,1:40)) function in Seurat. The output of this procedure represents a cell graph (‘wsnn’) that was used as input for UMAP visualization, and graph-based clustering. We performed UMAP visualization using the RunUMAP command in Seurat with default parameters, and clustering using the FindClusters function in Seurat (arguments to FindClusters: graph.name = ‘wsnn’, resolution = 1). We performed differential expression on all pairs of clusters for both RNA and protein markers, and merged clusters that did not exhibit clear evidence of separation, or where the only differentially expressed features represented ribosomal genes or mitochondrial genes. In some cases (particularly for extremely rare cell types that required a higher resolution to be correctly annotated in our clustering), we increased the granularity of our clustering by subsetting cells in an individual cluster, and rerunning FindClusters on this subgraph. We initially categorized these clusters into eight broad Level 1 groups, which were then further subdivided into 30 level 2 annotation categories that represented well-defined subtypes of human immune cells. These subtypes were annotated manually, but with the assistance of a previously defined set of markers from a CITE-seq reference of circulating human immune cells^17^. Our 47 level-3 clusters represent the highest level of granularity using the markers listed in Supplementary Table 6.

### Differential cell-type abundance analysis using Milo

To identify differentially abundant cell states between day 0 and day 28, we used Milo^18^ to analyze a WNN graph generated from CITE-seq data. The precomputed shared nearest neighbor graph (‘wsnn’) was first used as input required for Milo using the ‘buildFromAdjacency’ function (k = 20, d = 30). Next, cells were assigned into representative neighborhoods by running the ‘makeNhoods’ function (refined=TRUE, prop=0.1, refinement_scheme = ‘graph’). Cells were counted in neighborhoods using ‘countCells’ function. To test for differential abundance, the ‘testNhoods’ function was run (fdr.weighting = ‘graph-overlap’) with design = ~batch + timepoint. Neighborhoods with SpatialFDR < 0.1 were determined as statistically significant for differential abundance, and were colored in Fig. 1d,e.

### Gene module score

To examine the strength of interferon response, we downloaded the list of genes that upregulated in response to alpha and gamma interferon proteins from GESA website (https://www.gsea-msigdb.org/). We used the ‘AddModuleScore’ function in Seurat to quantify the expression of this gene module in single cells. In Fig. 1c and Extended Data Fig. 2c,d, one donor was excluded due to aberrant interferon expression at day 28.

To identify a module of genes that were biomarkers of vaccine-induced cells, we performed differential expression analysis. We used the ‘FindMarkers’ command in Seurat to compare expression of levels of VI-A CD8^+^ T cells with CD8_TEM_3 cells (the most similar CD8^+^ T cell cluster at level-3 resolution). We selected the top 200 genes (ranked by adjusted *P* value) with adjusted *P* value < 0.001 and minimal logFC threshold > 0.2. To ensure that our module was not contaminated by cell-cycle genes, we conservatively removed three genes that exhibited minimal upregulation in VI-A CD8^+^ T cells, but were strongly upregulated in VI-B CD8^+^ T cells. The resulting 197-gene list is included in Supplementary Table 3.

### Mapping of ASAP-seq data with bridge integration

To analyze the ASAP-seq dataset (Fig. 2), we used our recently developed ‘bridge integration’ workflow^25^, which integrates datasets that measure different modalities (that is, scATAC-seq and scRNA-seq data) based on a ‘bridge’ dataset, where both modalities are measured simultaneously (that is a 10x multiome dataset). We downloaded a publicly available multiome dataset from 10x Genomics (https://www.10xgenomics.com/resources/datasets/pbmc-from-a-healthy-donor-granulocytes-removed-through-cell-sorting-10-k-1-standard-2-0-0), consisting of 11,351 paired scRNA-seq and scATAC-seq profiles of human PBMC, and used this as a bridge dataset to annotate each of our 78,677 ASAP-seq profiles.

To perform annotation, we followed the steps detailed in the cross-modality reference mapping Seurat vignette (https://satijalab.org/seurat/articles/bridge_integration_vignette.html), utilizing our CITE-seq dataset (Fig. 1b) as a reference, and our ASAP-seq dataset as a query. The output of the bridge integration procedure includes multi-level cell annotations for each ASAP-seq profile, and additionally, visualizes the ASAP-seq dataset alongside our previously CITE-seq derived UMAP embedding.

We also performed further downstream analysis of the ASAP-seq dataset, based on the cell annotations derived from bridge integration. For these analyses, we performed TF-IDF normalization using the RunTFIDF function in Signac^54^ with default parameters. We used normalized values to calculate ‘gene activity’ scores, which serve as a proxy for expression levels based on the average chromatin accessibility within and upstream of a gene body, using the GeneActivity function in Signac. To identify differentially accessible peaks in vaccine-induced cells, we used the ‘FindMarkers’ function in Seurat, utilizing a logistic-regression based test^55^ (arguments, test.use = ‘LR’, latent.vars = ‘peak_region_fragments’), including cell-specific fragment count information to alleviate differences in cellular sequencing depth. The full list of differential peaks is included in Supplementary Table 5. We also used the top 1,000 differential peaks from this group as input to the FindMotifs function in Signac, which identifies enriched motifs from the JASPAR2022 database in this peak set compared to a background control set with matched GC content.

### Analysis of influenza vaccine ATAC-seq data

We downloaded and reanalyzed publicly available scATAC-seq data^28^ of samples before and after vaccination with the trivalent inactivated seasonal influenza vaccine (TIV) from GEO (GSE165906). We performed the same pre-processing steps as performed on our ASAP-seq dataset, using the 10x Genomics cellranger-atac software to align to the GRCh38 genome. One sample (donor ID: 79) was excluded as an outlier from downstream analysis due to a low unique fragment number per cell (1,158 reads/cell) compared with others (median: 7,576 reads/cell). We integrated the ATAC modality across biological samples from different donors and timepoints. We applied reciprocal LSI projection to find integration anchors by running the ‘FindIntegrationAnchors’ function in Seurat (reduction = ‘rlsi’, dims = 2:30). The final integration was conducted using the ‘IntegrateEmbeddings’ function to integrate the LSI coordinates across the datasets, returning a single 30-dimensional space (integrated_lsi). The integrated_lsi dimenstion of 2 to 30 were used as input for graph-based clustering, cell annotation, and UMAP visualization. To compare pseudobulk profiles of cells before and after vaccination, we quantified genomic bins using the ‘GenomeBinMatrix’ function in Signac (arguments: binsize = 5,000), retaining bins with at least one count.

### Visualization, clustering and annotation of ECCITE-seq data

Each ECCITE-seq profile simultaneously measures RNA and ADT modalities, but also measures immune repertoire sequences (TCR), as well as quantitative levels of the five MHC I Dextramers loaded with SARS-CoV-2 spike peptides. To analyze this dataset, we used WNN analysis to jointly define cell state based on three modalities: integrated RNA, integrated ADT and TCR. We also independently classified each cell as Dex^+^ or Dex^−^. Cells were classified as Dex^+^ if the UMI counts for any of the five spike protein dextramers were at least two times as high as the UMI counts for the negative control. We annotated each TCR clone as ‘spike-specific’ if any individual cell in the clone was annotated as Dex^+^.

Performing WNN analysis on multiple modalities requires a reduced-dimensional space to be independently generated for each modality. For RNA and ADT modalities, we generated this graph using the same normalization, data integration across samples and dimensional reduction steps as we performed in our CITE-seq WNN analysis. To learn a separate low-dimensional space based solely on TCR sequences, we used clonotype neighbor graph analysis (CoNGA^56^), which uses the TCRdist distance metric^57^ to quantify the similarity between two cells based on shared TCR sequence features. The script ‘setup_10x_for_conga.py’ was first run in CoNGA with ‘–no_kpca’ flag to prepare input files. The script ‘merge_samples.py’ was run next to merge the datasets from multiple 10x lanes. By running the ‘run_conga.py’ script with default settings, we performed kernel principal components analysis (kPCA) based on the TCRdist distance matrix and retained 40 components for downstream analysis. We used the three dimensional reductions (integrated RNA, integrated ADT, TCR) to perform a trimodal WNN analysis, which returned a single neighbor graph that integrated data from all three modalities. This graph was used as input for UMAP visualization, clustering and annotation (Fig. 3a).

We also annotated individual T cells as belonging to rare, small, medium, large or hyperexpanded clones using the scRepertoire^58^ package. The clonotype was called using the combination of the amino acid sequence of the CDR3 region for both the TCRα and TCRβ chains. The available chain was used for cells where only one of the two chains could be identified. For cells with multiple expressed chains, only the top two expressed chains were included for downstream analysis. We assigned clonal size for each cell by running the ‘clonalHomeostasis’ function in scRepertoire with the proportional cutpoints: (rare = 1 × 10^−4^; small = 0.001; medium = 0.01; large = 0.1; hyperexpanded = 1).

We compared each TCR with publicly available databases of T cells specific for SARS-CoV-2 peptide. We pooled TCRβ sequences from the ImmuneCODE COVID-19 TCR database^37^ and the VDJdb COVID-19 TCR database^38^. When comparing TCR from our vaccination dataset, we restricted our overlap analysis to spike protein epitopes.

### Analysis of publicly available SARS-CoV-2 vaccination and infection datasets

We downloaded a public vaccine CITE-seq dataset^13^ from GEO (GSE171964) and mapped these data using our previously described ‘reference-based mapping’ workflow^16^. One sample (donor id: 2055) was excluded from downstream analysis due to the low data quality of scRNA-seq on both day 7 and day 21. Our CITE-seq dataset was used as the reference, and RNA data from the public CITE-seq was used as the query. After identifying the anchors by running the ‘FindTransferAnchors’ function in Seurat, the query data was projected onto the reference UMAP with the transferred cell-type labels using ‘MapQuery’ function.

We obtained publicly available scRNA-seq dataset of acute SARS-CoV-2 infection samples^44^ from (https://zenodo.org/record/5770747). The UMAP in Fig. 4a is a reproduction of the visualization in the original manuscript. For further analyses, we used data from two individual sample sets: (1) patients CoV2_T001- CoV2_T010, acute; (2) patients CoV2_T011- CoV2_T020, acute. We retained cells with at least 500 detected UMI, mitochondrial read percentages lower than 15%, and where SNP-based demultiplexing was consistent with a single donor. As in the original manuscript^44^, we removed a particular dextramer (peptide QYIKWPWYI) in the downstream analysis due to high nonspecific binding. As in the original manuscript^44^, cells were labeled as CoV2-Dex^+^ when the UMI count of a CoV2-Dextramer was higher than 10 and the fold change versus the negative control was more than five.

We obtained publicly available datasets from the COvid-19 Multi-omics Blood ATlas (COMBAT) Consortium^46^, profiling human PBMC samples across multiple human donors at different stages of infection using ECCITE-seq (https://zenodo.org/record/6120249). We considered CD8^+^ T cells from healthy donors and patients with mild, severe or critical symptoms. Cells with fewer than 300 detected genes or mitochondrial read percentage higher than 10% were removed. Donors including less than 200 CD8^+^ T cells after QC were excluded. To perform integration across samples and modalities, we ran the same anchor-based integration procedure separately on the RNA and ADT modalities as we ran for our CITE-seq dataset. The WNN graph was generated using 30 RNA and 20 protein dimensions. The WNN graph was used as input for UMAP visualization and clustering.

### Statistics and reproducibility

No statistical method was used to predetermine sample size. The experiments were not randomized. The investigators were not blinded to allocation during experiments and outcome assessment. In Fig. 1c and Extended Data Fig. 2c,d, one donor was excluded due to aberrant interferon expression at day 28. The specific statistical tests conducted on the data, along with the respective sample sizes, are indicated in the figure legends. Individual *P* values are presented directly in the figures.

### Reporting summary

Further information on research design is available in the Nature Portfolio Reporting Summary linked to this article.

## Online content

Any methods, additional references, Nature Portfolio reporting summaries, source data, extended data, supplementary information, acknowledgements, peer review information; details of author contributions and competing interests; and statements of data and code availability are available at 10.1038/s41590-023-01608-9.

### Supplementary information

Supplementary Information Supplementary Tables 1–6.

Reporting Summary

## Data Availability

All raw sequencing data are deposited under dbGaP accession: phs003322.v1.p1. The processed datasets are available as open-access downloads at: https://zenodo.org/record/7555405. The vaccine CITE-seq dataset^13^ used in Extended Data Fig. 3 was available at: https://www.ncbi.nlm.nih.gov/geo/query/acc.cgi?acc=GSE171964. The scATAC-seq data^28^ of trivalent inactivated seasonal influenza vaccine was obtained at: https://www.ncbi.nlm.nih.gov/geo/query/acc.cgi?acc=GSE165906. The scRNA-seq dataset^44^ of acute SARS-CoV-2 infection used in Fig. 4 was obtained at: https://zenodo.org/record/5770747. The datasets from COvid-19 Multi-omics Blood ATlas^46^ (COMBAT) was available at: https://zenodo.org/record/6120249.

## Code availability

Seurat and Signac are freely available as open-source software packages at https://github.com/satijalab/seurat and https://github.com/timoast/signac, respectively. The scripts used for dataset processing can also be found at: https://zenodo.org/record/7555405
