# Supplementary material for: Multimodal single-cell datasets characterize antigen-specific CD8+ T cells across SARS-CoV-2 vaccination and infection
Source: Nat Immunol. 2023 Sep 21;24(10):1725–34. doi: 10.1038/s41590-023-01608-9 (PMC10522491; doi:10.1038/s41590-023-01608-9)
Supplement: Supplementary file 1 — Supplementary Tables 1–6. [file 41590_2023_1608_MOESM1_ESM.pdf]

nature immunology

Article https://doi.org/10.1038/s41590-023-01608-9

Multimodal single-cell datasets characterize antigen-specific CD8^{+} T cells across SARS-CoV-2 vaccination and infection

In the format provided by the authors and unedited

Supplemental Table 1

Donors for CITE-seq / ASAP-seq (Figures 1-2)

	Donor ID	Age	Gender	Race	Ethnicity
Batch 1	**A**	**23**	Female	Native American	Hispanic/Latino
Batch 1	**E**	**17**	Female	Native American	Hispanic/Latino
Batch 1	**F**	**26**	Female	Not reported	Not reported
Batch 2	**B**	**20**	Female	Not reported	Not of Hispanic origin
Batch 2	**J**	**21**	Male	Not reported	Not of Hispanic origin
Batch 2	**M**	**24**	Female	Not reported	Not of Hispanic origin

Donors for ECCITE-Seg (Figure 3)

	Donor ID	Age	Gender	Race	Ethnicity
Batch 1	**A**	**23**	Female	Native American	Hispanic/Latino
Batch 1	**E**	**17**	Female	Native American	Hispanic/Latino
Batch 1	**P**	**31**	Female	Not reported	Not reported
Batch 1	**D16**	**53**	Male	White	Not of Hispanic origin
Batch 2	**D40**	**29**	Female	White	Not of Hispanic origin
Batch 2	**D87**	**57**	Male	White	Not of Hispanic origin
Batch 2	**D93**	**33**	Female	White	Not of Hispanic origin
Batch 2	**D95**	**58**	Female	Not reported	Not reported
Batch 2	**P**	**31**	Female	Not reported	Not reported
Batch 2	**D16**	**53**	Male	White	Not of Hispanic origin

Supplementary table 2

Total-seq A	Description	Clone	Barcode	Gene Name
A0006	anti-human CD86	IT2.2	GTCTTTGTCAGTGCA	CD86
A0007	anti-human CD274 (B7-H1, PD-L1)	29E.2A3	GTTGTCCGACAATAC	CD274
A0020	anti-human CD270 (HVEM, TR2)	122	TGATAGAAACAGACC	TNFRSF14
A0023	anti-human CD155 (PVR)	SKII.4	ATCACATCGTTGCCA	PVR
A0024	anti-human CD112 (Nectin-2)	TX31	AACCTTCCGTCTAAG	NECTIN2
A0026	anti-human CD47	CC2C6	GCATTCTGTCACCTA	CD47
A0029	anti-human CD48	BJ40	CTACGACGTAGAAGA	CD48
A0031	anti-human CD40	5C3	CTCAGATGGAGTATG	CD40
A0032	anti-human CD154	24-31	GCTAGATAGATGCAA	CD40LG
A0033	anti-human CD52	HI186	CTTTGTACGAGCAAA	CD52
A0034	anti-human CD3	UCHT1	CTCATTGTAACTCCT	CD3D
A0046	anti-human CD8	SK1	GCGCAACTTGATGAT	CD8A
A0047	anti-human CD56 (NCAM)	5.1H11	TCCTTTCCTGATAGG	NCAM1
A0050	anti-human CD19	HIB19	CTGGGCAATTACTCG	CD19
A0052	anti-human CD33	P67.6	TAACTCAGGGCCTAT	CD33
A0053	anti-human CD11c	S-HCL-3	TACGCCTATAACTTG	ITGAX
A0058	anti-human HLA-A,B,C	W6/32	TATGCGAGGCTTATC	HLA-A
A0063	anti-human CD45RA	HI100	TCAATCCTTCCGCTT	PTPRC
A0064	anti-human CD123	6H6	CTTCACTCTGTCAGG	IL3RA
A0066	anti-human CD7	CD7-6B7	TGGATTCCCGGACTT	CD7
A0070	anti-human/mouse CD49f	GoH3	TTCCGAGGATGATCT	ITGA6
A0071	anti-human CD194 (CCR4)	L291H4	AGCTTACCTGCACGA	CCR4
A0072	anti-human CD4	RPA-T4	TGTTCCCGCTCAACT	CD4
A0073	anti-mouse/human CD44	IM7	TGGCTTCAGGTCCTA	CD44
A0081	anti-human CD14	M5E2	TCTCAGACCTCCGTA	CD14
A0083	anti-human CD16	3G8	AAGTTCACTCTTTGC	FCGR3A
A0085	anti-human CD25	BC96	TTTGTCCTGTACGCC	IL2RA
A0087	anti-human CD45RO	UCHL1	CTCCGAATCATGTTG	PTPRC
A0088	anti-human CD279 (PD-1)	EH12.2H7	ACAGCGCCGTATTTA	PDCD1
A0089	anti-human TIGIT (VSTM3)	A15153G	TTGCTTACCGCCAGA	TIGIT
A0090	Mouse IgG1, κ isotype Ctrl	MOPC-21	GCCGGACGACATTAA	
A0091	Mouse IgG2a, κ isotype Ctrl	MOPC-173	CTCCTACCTAAACTG	

A0092	Mouse IgG2b, κ isotype Ctrl	MPC-11	ATATGTATCACGCGA	
A0095	Rat IgG2b, κ Isotype Ctrl	RTK4530	GATTCTTGACGACCT	
A0100	anti-human CD20	2H7	TTCTGGGTCCCTAGA	MS4A1
A0101	anti-human CD335 (NKp46)	9E2	ACAATTTGAACAGCG	NCR1
A0124	anti-human CD31	WM59	ACCTTTATGCCACGG	PECAM1
A0127	anti-Human Podoplanin	NC-08	GGTTACTCGTTGTGT	PDPN
A0134	anti-human CD146	P1H12	CCTTGGATAACATCA	MCAM
A0136	anti-human IgM	MHM-88	TAGCGAGCCCGTATA	IGHM
A0138	anti-human CD5	UCHT2	CATTAACGGGATGCC	CD5
A0140	anti-human CD183 (CXCR3)	G025H7	GCGATGGTAGATTAT	CXCR3
A0141	anti-human CD195 (CCR5)	J418F1	CCAAAGTAAGAGCCA	CCR5
A0142	anti-human CD32	FUN-2	GCTTCCGAATTACCG	FCGR2A
A0143	anti-human CD196 (CCR6)	G034E3	GATCCCTTTGTCACT	CCR6
A0144	anti-human CD185 (CXCR5)	J252D4	AATTCAACCGTCGCC	CXCR5
A0145	Hu CD103 (Integrin αE)	Ber-ACT8	GACCTCATTGTGAAT	ITGAE
A0146	anti-human CD69	FN50	GTCTCTTGGCTTAAA	CD69
A0147	anti-human CD62L	DREG-56	GTCCCTGCAACTTGA	SELL
A0149	anti-human CD161	HP-3G10	GTACGCAGTCCTTCT	KLRB1
A0151	anti-human CD152 (CTLA-4)	BNI3	ATGGTTCACGTAATC	CTLA4
A0152	anti-human CD223 (LAG-3)	11C3C65	CATTTGTCTGCCGGT	LAG3
A0153	anti-human KLRG1 (MAFA)	SA231A2	CTTATTTCCTGCCCT	KLRG1
A0154	anti-human CD27	O323	GCACTCCTGCATGTA	CD27
A0155	anti-human CD107a (LAMP-1)	H4A3	CAGCCCACTGCAATA	LAMP1
A0156	anti-human CD95 (Fas)	DX2	CCAGCTCATTAGAGC	FAS
A0158	anti-human CD134 (OX40)	er-ACT35 (ACT3	AACCCACCGTTGTTA	TNFRSF4
A0159	anti-human HLA-DR	L243	AATAGCGAGCAAGTA	HLA-DRA
A0160	anti-human CD1c	L161	GAGCTACTTCACTCG	CD1C
A0161	anti-human CD11b	ICRF44	GACAAGTGATCTGCA	ITGAM
A0162	anti-human CD64	10.1	AAGTATGCCCTACGA	FCGR1A
A0163	anti-human CD141 (Thrombomodulin)	M80	GGATAACCGCGCTTT	THBD
A0165	Hu CD314 (NKG2D)	1D11	CGTGTTTGTTCCTCA	KLRK1
A0167	anti-human CD35	E11	ACTTCCGTCGATCTT	CR1
A0168	anti-human CD57 Recombinant	QA17A04	AACTCCCTATGGAGG	B3GAT1

A0170	anti-human CD272 (BTLA)	MIH26	GTTATTGGACTAAGG	BTLA
A0171	anti-human/mouse/rat CD278 (ICOS)	C398.4A	CGCGCACCCATTAAA	ICOS
A0172	anti-human CD275 (B7-H2, B7-RP1, ICOSL)	9F.8A4	GTTAGTGTTAGCTTG	ICOSLG
A0174	anti-human CD58 (LFA-3)	TS2/9	GTTCCTATGGACGAC	CD58
A0176	anti-human CD39	A1	TTACCTGGTATCCGT	ENTPD1
A0179	anti-human CX3CR1	K0124E1	AGTATCGTCTCTGGG	CX3CR1
A0180	anti-human CD24	ML5	AGATTCCTTCGTGTT	CD24
A0181	anti-human CD21	Bu32	AACCTAGTAGTTCGG	CR2
A0185	anti-human CD11a	TS2/4	TATATCCTTGTGAGC	ITGAL
A0187	anti-human CD79b (Igβ)	CB3-1	ATTCTTCAACCGAAG	CD79B
A0189	anti-human CD244 (2B4)	C1.7	TCGCTTGGATGGTAG	CD244
A0206	anti-human CD169 (Sialoadhesin, Siglec-1)	7-239	TACTCAGCGTGTTTG	SIGLEC1
A0214	anti-human/mouse integrin β7	FIB504	TCCTTGGATGTACCG	ITGB7
A0215	anti-human CD268 (BAFF-R)	11C1	CGAAGTCGATCCGTA	TNFRSF13C
A0216	anti-human CD42b	HIP1	TCCTAGTACCGAAGT	GP1BB
A0217	anti-human CD54	HA58	CTGATAGACTTGAGT	ICAM1
A0218	anti-human CD62P (P-Selectin)	AK4	CCTTCCGTATCCCTT	SELP
A0219	anti-human CD119 (IFN-γ R α chain)	GIR-208	TGTGTATTCCCTTGT	IFNGR1
A0224	anti-human TCR α/β	IP26	CGTAACGTAGAGCGA	
A0236	Rat IgG1, κ isotype Ctrl	RTK2071	ATCAGATGCCCTCAT	
A0237	Rat IgG1, λ Isotype Ctrl	G0114F7	GGGAGCGATTCAACT	
A0238	Rat IgG2a, κ Isotype Ctrl	RTK2758	AAGTCAGGTTCGTTT	
A0240	Rat IgG2c, κ Isotype Ctrl	RTK4174	TCCAGGCTAGTCATT	
A0241	Armenian Hamster IgG Isotype Ctrl	HTK888	CCTGTCATTAAGACT	
A0242	anti-human CD192 (CCR2)	K036C2	GAGTTCCCTTACCTG	CCR2
A0246	anti-human CD122 (IL-2Rβ)	TU27	TCATTTCCTCCGATT	IL2RB
A0247	anti-human CD267 (TACI)	1A1	AGTGATGGAGCGAAC	TNFRSF13B
A0352	anti-human FcεRIα	AER-37 (CRA-1)	CTCGTTTCCGTATCG	FCER1A
A0353	anti-human CD41	HIP8	ACGTTGTGGCCTTGT	ITGA2B
A0355	anti-human CD137 (4-1BB)	4B4-1	CAGTAAGTTCGGGAC	TNFRSF9
A0357	anti-human CD43	CD43-10G7	GATTAACCAGCTCAT	SPN
A0358	anti-human CD163	GHI/61	GCTTCTCCTTCCTTA	CD163
A0359	anti-human CD83	HB15e	CCACTCATTTCCGGT	CD83

A0364	anti-human CD13	WM15	TTTCAACGCCCTTTC	ANPEP
A0367	anti-human CD2	TS1/8	TACGATTTGTCAGGG	CD2
A0368	anti-human CD226 (DNAM-1)	11A8	TCTCAGTGTTTGTGG	CD226
A0369	anti-human CD29	TS2/16	GTATTCCCTCAGTCA	ITGB1
A0370	anti-human CD303 (BDCA-2)	201A	GAGATGTCCGAATTT	CLEC4C
A0371	anti-human CD49b	P1E6-C5	GCTTTCTTCAGTATG	ITGA2
A0372	anti-human CD61	VI-PL2	AGGTTGGAGTAGACT	ITGB3
A0373	anti-human CD81 (TAPA-1)	5A6	GTATCCTTCCTTGGC	CD81
A0383	anti-human CD55	JS11	GCTCATTACCCATTA	CD55
A0384	anti-human IgD	IA6-2	CAGTCTCCGTAGAGT	IGHD
A0385	anti-human CD18	TS1/18	TATTGGGACACTTCT	ITGB2
A0386	anti-human CD28	CD28.2	TGAGAACGACCCTAA	CD28
A0389	anti-human CD38	HIT2	TGTACCCGCTTGTGA	CD38
A0390	anti-human CD127 (IL-7Rα)	A019D5	GTGTGTTGTCCTATG	IL7R
A0391	anti-human CD45	HI30	TGCAATTACCCGGAT	PTPRC
A0393	anti-human CD22	S-HCL-1	GGGTTGTTGTCTTTG	CD22
A0394	anti-human CD71	CY1G4	CCGTGTTCCTCATTA	TFRC
A0396	anti-human CD26	BA5b	GGTGGCTAGATAATG	DPP4
A0398	anti-human CD115 (CSF-1R)	9-4D2-1E4	AATCACGGTCCTTGT	CSF1R
A0404	anti-human CD63	H5C6	GAGATGTCTGCAACT	CD63
A0406	anti-human CD304 (Neuropilin-1)	12C2	GGACTAAGTTTCGTT	NRP1
A0407	anti-human CD36	5-271	TTCTTTGCCTTGCCA	CD36
A0408	anti-human CD172a (SIRPα)	15-414	CGTGTTTAACTTGAG	SIRPA
A0419	anti-human CD72	3F3	CAGTCGTGGTAGATA	CD72
A0420	anti-human CD158 (KIR2DL1/S1/S3/S5)	HP-MA4	TATCAACCAACGCTT	KIR2DL1
A0446	anti-human CD93	VIMD2	GCGCTACTTCCTTGA	CD93
A0447	anti-human CD200 (OX2)	OX-104	CACGTAGACCTTTGC	CD200
A0575	anti-human CD49a	TS2/7	ACTGATGGACTCAGA	ITGA1
A0576	anti-human CD49d	9F10	CCATTCAACTTCCGG	ITGA4
A0577	anti-human CD73 (Ecto-5'-nucleotidase)	AD2	CAGTTCCTCAGTTCG	NT5E
A0579	anti-human CD9	HI9a	GAGTCACCAATCTGC	CD9
A0581	anti-human TCR Vα7.2	3C10	TACGAGCAGTATTCA	
A0582	anti-human TCR Vδ2	B6	TCAGTCAGATGGTAT	

A0586	anti-human CD354 (TREM-1)	TREM-26	TAGCCGTTTCCTTTG	TREM1
A0590	anti-human CD305 (LAIR1)	NKTA255	ATTTCCATTCCCTGT	LAIR1
A0591	anti-human LOX-1	15C4	ACCCTTTACCGAATA	OLR1
A0599	anti-human CD158e1 (KIR3DL1, NKB1)	DX9	GGACGCTTTCCTTGA	KIR3DL1
A0817	anti-human CD109	W7C5	CACTTAACTCTGGGT	CD109
A0822	anti-human CD142	NY2	CACTGCCGTCGATTA	F3
A0830	anti-human CD319 (CRACC)	162.1	AGTATGCCATGTCTT	SLAMF7
A0845	anti-human CD99	3B2/TA8	ACCCGTCCCTAAGAA	CD99
A0853	anti-human CLEC12A	50C1	CATTAGAGTCTGCCA	CLEC12A
A0861	anti-human CD151 (PETA-3)	50-6	CTTACCTAGTCATTC	CD151
A0864	anti-human CD352 (NTB-A)	NT-7	AGTTTCCACTCAGGC	SLAMF6
A0866	anti-human CLEC1B (CLEC2)	AYP1	TGCCAGTATCACGTA	CLEC1B
A0867	anti-human CD94	DX22	CTTTCCGGTCCTACA	KLRD1
A0868	anti-human IgE	MHE-18	GGATGTACCGCGTAT	IGHE
A0870	anti-human CD150 (SLAM)	A12 (7D4)	GTCATTGTATGTCTG	SLAMF1
A0871	anti-human CD162	KPL-1	ATATGTCAGAGCACC	SELPLG
A0872	anti-human CD84	CD84.1.21	CTCCCTAGTTCCTTT	CD84
A0894	anti-human Ig light chain κ	MHK-49	AGCTCAGCCAGTATG	IGKC
A0896	anti-human CD85j (ILT2)	GHI/75	CCTTGTGAGGCTATG	LILRB1
A0897	anti-human CD23	EBVCS-5	TCTGTATAACCGTCT	FCER2
A0898	anti-human Ig light chain λ	MHL-38	CAGCCAGTAAGTCAC	
A0902	anti-human CD328 (Siglec-7)	6-434	CTTAGCATTTCACTG	SIGLEC7
A0912	anti-human GPR56	CG4	GCCTAGTTTCCGTTT	ADGRG1
A0920	anti-human CD82	ASL-24	TCCCACTTCCGCTTT	CD82
A0923	anti-human NKp80	5D12	TATAGTTCCTCTGTG	KLRF1
A0931	anti-human CD131	1C1	CTGCATGAGACCAAA	CSF2RB
A0935	anti-human CD74	LN2	CTGTAGCATTTCCCT	CD74
A0940	anti-human CD116	4H1	ATGGACAGTTCGTGT	CSF2RA
A0941	anti-human CD37	M-B371	ACAGTCACTGGGCAA	CD37
A0944	anti-human CD101 (BB27)	BB27	CTACTTCCCTGTCAA	CD101
A1018	anti-human HLA-DR, DP, DQ	Tü39	AGCTACGAGCAGTAG	HLA-DRA
A1046	anti-human CD88 (C5aR)	S5/1	GCCGCATGAGAAACA	C5AR1
	CD110	S16017E	TGTTGTAAGATGCCA	

GP130	2E1B02	CACGAGAATTTCAGT
CD337	P30-15	AAAGTCACTCTGCCG
CD271	ME20.4	AACCGCGCTTCAGAT
CD34	581	GCAGAAATCTCCCTT
CD117	104D2	AGACTAATAGCTGAC
CD1d	51.1	TCGAGTCGCTTATCA
CD126	UV4	TGATGGGAGCTTATC
CD307e	509f6	TCACGCAGTCCTCAA
CD307c/FcRL3	H5/FcRL3	GCCTAGTTTGAACGC

Total-seq C	Description	Clone	Barcode
C0006	anti-human CD86	IT2.2	GTCTTTGTCAGTGCA
C0007	anti-human CD274 (B7-H1, PD-L1)	29E.2A3	GTTGTCCGACAATAC
C0020	anti-human CD270 (HVEM, TR2)	122	TGATAGAAACAGACC
C0023	anti-human CD155 (PVR)	SKII.4	ATCACATCGTTGCCA
C0024	anti-human CD112 (Nectin-2)	TX31	AACCTTCCGTCTAAG
C0026	anti-human CD47	CC2C6	GCATTCTGTCACCTA
C0029	anti-human CD48	BJ40	CTACGACGTAGAAGA
C0031	anti-human CD40	5C3	CTCAGATGGAGTATG
C0032	anti-human CD154	24-31	GCTAGATAGATGCAA
C0033	anti-human CD52	HI186	CTTTGTACGAGCAAA
C0034	anti-human CD3	UCHT1	CTCATTGTAACTCCT
C0046	anti-human CD8	SK1	GCGCAACTTGATGAT
C0047	anti-human CD56	5.1H11	TCCTTTCCTGATAGG
C0050	anti-human CD19	HIB19	CTGGGCAATTACTCG
C0052	anti-human CD33	P67.6	TAACTCAGGGCCTAT
C0053	anti-human CD11c	S-HCL-3	TACGCCTATAACTTG
C0058	anti-human HLA-A,B,C	W6/32	TATGCGAGGCTTATC
C0063	anti-human CD45RA	HI100	TCAATCCTTCCGCTT
C0064	anti-human CD123	6H6	CTTCACTCTGTCAGG
C0066	anti-human CD7	CD7-6B7	TGGATTCCCGGACTT
C0068	anti-human CD105	43A3	ATCGTCGAGAGCTAG
C0070	anti-human/mouse CD49f	GoH3	TTCCGAGGATGATCT
C0071	anti-human CD194 (CCR4)	L291H4	AGCTTACCTGCACGA
C0072	anti-human CD4	RPA-T4	TGTTCCCGCTCAACT
C0073	anti-mouse/human CD44	IM7	TGGCTTCAGGTCCTA
C0081	anti-human CD14	M5E2	TCTCAGACCTCCGTA
C0083	anti-human CD16	3G8	AAGTTCACTCTTTGC
C0085	anti-human CD25	BC96	TTTGTCCTGTACGCC
C0087	anti-human CD45RO	UCHL1	CTCCGAATCATGTTG
C0088	anti-human CD279	EH12.2H7	ACAGCGCCGTATTTA
C0089	anti-human TIGIT (VSTM3)	A15153G	TTGCTTACCGCCAGA
C0090	Mouse IgG1, κ isotype Ctrl	MOPC-21	GCCGGACGACATTAA
C0091	Mouse IgG2a, κ isotype Ctrl	MOPC-173	CTCCTACCTAAACTG
C0092	Mouse IgG2b, κ isotype Ctrl	MPC-11	ATATGTATCACGCGA
C0095	Rat IgG2b, κ Isotype Ctrl	RTK4530	GATTCTTGACGACCT
C0100	anti-human CD20	2H7	TTCTGGGTCCCTAGA
C0101	anti-human CD335 (NKp46)	9E2	ACAATTTGAACAGCG
C0124	anti-human CD31	WM59	ACCTTTATGCCACGG
C0134	anti-human CD146	P1H12	CCTTGGATAACATCA
C0136	anti-human IgM	MHM-88	TAGCGAGCCCGTATA
C0138	anti-human CD5	UCHT2	CATTAACGGGATGCC
C0140	anti-human CD183 (CXCR3)	G025H7	GCGATGGTAGATTAT
C0141	anti-human CD195 (CCR5)	J418F1	CCAAAGTAAGAGCCA
C0142	anti-human CD32	FUN-2	GCTTCCGAATTACCG

C0143	anti-human CD196 (CCR6)	G034E3	GATCCCTTTGTCACT
C0144	anti-human CD185 (CXCR5)	J252D4	AATTCAACCGTCGCC
C0145	anti-human CD103 (Integrin αE)	Ber-ACT8	GACCTCATTGTGAAT
C0146	anti-human CD69	FN50	GTCTCTTGGCTTAAA
C0147	anti-human CD62L	DREG-56	GTCCCTGCAACTTGA
C0149	anti-human CD161	HP-3G10	GTACGCAGTCCTTCT
C0151	anti-human CD152 (CTLA-4)	BNI3	ATGGTTCACGTAATC
C0152	anti-human CD223 (LAG-3)	11C3C65	CATTTGTCTGCCGGT
C0153	anti-human KLRG1 (MAFA)	SA231A2	CTTATTTCCTGCCCT
C0154	anti-human CD27	O323	GCACTCCTGCATGTA
C0155	anti-human CD107a (LAMP-1)	H4A3	CAGCCCACTGCAATA
C0156	anti-human CD95 (Fas)	DX2	CCAGCTCATTAGAGC
C0158	anti-human CD134 (OX40)	-ACT35 (ACT	AACCCACCGTTGTTA
C0159	anti-human HLA-DR	L243	AATAGCGAGCAAGTA
C0160	anti-human CD1c	L161	GAGCTACTTCACTCG
C0161	anti-human CD11b	ICRF44	GACAAGTGATCTGCA
C0162	anti-human CD64	10.1	AAGTATGCCCTACGA
C0163	anti-human CD141 (Thrombomodulin)	M80	GGATAACCGCGCTTT
C0164	anti-human CD1d	51.1	TCGAGTCGCTTATCA
C0165	anti-human CD314 (NKG2D)	1D11	CGTGTTTGTTCCTCA
C0167	anti-human CD35	E11	ACTTCCGTCGATCTT
C0168	anti-human CD57 Recombinant	QA17A04	AACTCCCTATGGAGG
C0170	anti-human CD272 (BTLA)	MIH26	GTTATTGGACTAAGG
C0171	anti-human/mouse/rat CD278 (ICOS)	C398.4A	CGCGCACCCATTAAA
C0174	anti-human CD58 (LFA-3)	TS2/9	GTTCCTATGGACGAC
C0176	anti-human CD39	A1	TTACCTGGTATCCGT
C0179	anti-human CX3CR1	K0124E1	AGTATCGTCTCTGGG
C0180	anti-human CD24	ML5	AGATTCCTTCGTGTT
C0181	anti-human CD21	Bu32	AACCTAGTAGTTCGG
C0185	anti-human CD11a	TS2/4	TATATCCTTGTGAGC
C0187	anti-human CD79b (Igβ)	CB3-1	ATTCTTCAACCGAAG
C0189	anti-human CD244 (2B4)	C1.7	TCGCTTGGATGGTAG
C0206	nti-human CD169 (Sialoadhesin, Siglec-1	7-239	TACTCAGCGTGTTTG
C0214	anti-human/mouse integrin β7	FIB504	TCCTTGGATGTACCG
C0215	anti-human CD268 (BAFF-R)	11C1	CGAAGTCGATCCGTA
C0216	anti-human CD42b	HIP1	TCCTAGTACCGAAGT
C0217	anti-human CD54	HA58	CTGATAGACTTGAGT
C0218	anti-human CD62P (P-Selectin)	AK4	CCTTCCGTATCCCTT
C0219	anti-human CD119 (IFN-γ R α chain)	GIR-208	TGTGTATTCCCTTGT
C0224	anti-human TCR α/β	IP26	CGTAACGTAGAGCGA
C0236	Rat IgG1, κ isotype Ctrl	RTK2071	ATCAGATGCCCTCAT
C0238	Rat IgG2a, κ Isotype Ctrl	RTK2758	AAGTCAGGTTCGTTT
C0241	Armenian Hamster IgG Isotype Ctrl	HTK888	CCTGTCATTAAGACT
C0246	anti-human CD122 (IL-2Rβ)	TU27	TCATTTCCTCCGATT
C0247	anti-human CD267 (TACI)	1A1	AGTGATGGAGCGAAC

C0352	anti-human FcεRIα	ER-37 (CRA-1	CTCGTTTCCGTATCG
C0353	anti-human CD41	HIP8	ACGTTGTGGCCTTGT
C0355	anti-human CD137 (4-1BB)	4B4-1	CAGTAAGTTCGGGAC
C0358	anti-human CD163	GHI/61	GCTTCTCCTTCCTTA
C0359	anti-human CD83	HB15e	CCACTCATTTCCGGT
C0363	anti-human CD124 (IL-4Rα)	G077F6	CCGTCCTGATAGATG
C0364	anti-human CD13	WM15	TTTCAACGCCCTTTC
C0367	anti-human CD2	TS1/8	TACGATTTGTCAGGG
C0368	anti-human CD226 (DNAM-1)	11A8	TCTCAGTGTTTGTGG
C0369	anti-human CD29	TS2/16	GTATTCCCTCAGTCA
C0370	anti-human CD303 (BDCA-2)	201A	GAGATGTCCGAATTT
C0371	anti-human CD49b	P1E6-C5	GCTTTCTTCAGTATG
C0373	anti-human CD81 (TAPA-1)	5A6	GTATCCTTCCTTGGC
C0384	anti-human IgD	IA6-2	CAGTCTCCGTAGAGT
C0385	anti-human CD18	TS1/18	TATTGGGACACTTCT
C0386	anti-human CD28	CD28.2	TGAGAACGACCCTAA
C0389	anti-human CD38	HIT2	TGTACCCGCTTGTGA
C0390	anti-human CD127 (IL-7Rα)	A019D5	GTGTGTTGTCCTATG
C0391	anti-human CD45	HI30	TGCAATTACCCGGAT
C0393	anti-human CD22	S-HCL-1	GGGTTGTTGTCTTTG
C0394	anti-human CD71	CY1G4	CCGTGTTCCTCATTA
C0396	anti-human CD26	BA5b	GGTGGCTAGATAATG
C0407	anti-human CD36	5-271	TTCTTTGCCTTGCCA
C0420	anti-human CD158 (KIR2DL1/S1/S3/S5)	HP-MA4	TATCAACCAACGCTT
C0575	anti-human CD49a	TS2/7	ACTGATGGACTCAGA
C0576	anti-human CD49d	9F10	CCATTCAACTTCCGG
C0577	anti-human CD73 (Ecto-5'-nucleotidase)	AD2	CAGTTCCTCAGTTCG
C0581	anti-human TCR Vα7.2	3C10	TACGAGCAGTATTCA
C0582	anti-human TCR Vδ2	B6	TCAGTCAGATGGTAT
C0591	anti-human LOX-1	15C4	ACCCTTTACCGAATA
C0592	nti-human CD158b (KIR2DL2/L3, NKAT2	DX27	GACCCGTAGTTTGAT
C0599	anti-human CD158e1 (KIR3DL1, NKB1)	DX9	GGACGCTTTCCTTGA
C0830	anti-human CD319 (CRACC)	162.1	AGTATGCCATGTCTT
C0845	anti-human CD99	3B2/TA8	ACCCGTCCCTAAGAA
C0853	anti-human CLEC12A	50C1	CATTAGAGTCTGCCA
C0864	anti-human CD352 (NTB-A)	NT-7	AGTTTCCACTCAGGC
C0867	anti-human CD94	DX22	CTTTCCGGTCCTACA
C0894	anti-human Ig light chain κ	MHK-49	AGCTCAGCCAGTATG
C0896	anti-human CD85j (ILT2)	GHI/75	CCTTGTGAGGCTATG
C0897	anti-human CD23	EBVCS-5	TCTGTATAACCGTCT
C0898	anti-human Ig light chain λ	MHL-38	CAGCCAGTAAGTCAC
C0902	anti-human CD328 (Siglec-7)	6-434	CTTAGCATTTCACTG
C0912	anti-human GPR56	CG4	GCCTAGTTTCCGTTT
C0918	anti-human HLA-E	3D12	GAGTCGAGAAATCAT
C0920	anti-human CD82	ASL-24	TCCCACTTCCGCTTT

C0944	anti-human CD101 (BB27)	BB27	CTACTTCCCTGTCAA
C1046	anti-human CD88 (C5aR)	S5/1	GCCGCATGAGAAACA
C1052	anti-human CD224	KF29	CTGATGAGATGTCAG

Marker	Fluorescent Channel	Dilution	Antibody clone	Vendor
Fixable Blue	Ultraviolet	1:400		ThermoFisher L34962
CD8	APC	2:100	SK1	Biolegend 344721
Dump CD14	BV510 / AmCyan	2:100	M5E2	Biolegend 301841
Dump CD16	BV510 / AmCyan	2:100	3G8	Biolegend 302047
Dump CD20	BV510 / AmCyan	2:100	2H7	Biolegend 302339
CD2	FITC	2:100	TS1/8	Biolegend 309205
CD71	PE-Cy7	3:100	CY1G4	Biolegend 334111
CD38	BV421 / Pacific Blue	2:100	HIT2	Biolegend 303525
CD4	PE	2:100	RPA-T4	Biolegend 300507
CD45RO	BV605	2:100	UCHL1	Biolegend 304237
CD45RA	PE-Dazzle 594	2:100	HI100	Biolegend 304145
HLA-DR	BV785	2:100	L243	Biolegend 307641
ICOS	PerCP/Cyanine5.5	2:100	C398.4A	Biolegend 313517
KLRG1	APC/Fire 750	2:100	SA231A2	Biolegend 367717
Ki-67	BUV395	3:100	B56	BD Biosciences 564071

Supplementary table 3

VI-GEM
ACTG1
PCLAF
ACTB
GAPDH
CD38
GZMH
PFN1
CORO1A
PYCARD
PSMB8
TMSB10
ARPC3
CLIC1
CD52
TMSB4X
APOBEC3H
ADA
SEM1
MYL6
GZMA
DECR1
ARPC2
NOP10
APOBEC3C
LGALS1
GZMB
CFL1
CLTA
TWF2
ISG15
MXD4
SUB1
PSMA4
HLA-DRA
PPP1R18
GSTO1
APOBEC3G
ARPC5
UCP2
PSMA5
WDR1
ARHGDIB

YWHAE
CCL5
SLC25A5
PSMB4
ITGB1
DYNLL1
ARPC4
ATP5MC2
FGFBP2
GTF3C6
RGS10
ATP6V1D
S1PR4
EZH2
ATP5F1A
GMFG
OSTF1
PRF1
ABRACL
GLRX
ACTR3
SIT1
TXNDC17
H3F3A
CCDC28B
ATP5MG
DNAJC15
IDH2
SEC11A
FABP5
CAPZB
DBI
IFI16
DCTN2
COTL1
CNN2
CHMP2A
ATP5MC3
ARPC1B
NDUFS2
MT1E
MRPL28
OAS2

SERPINB1
CARHSP1
TIMD4
UBE2L6
MYL6B
DCTN3
IRF4
ANXA5
ATP5F1B
GIMAP4
PSMB2
NDUFB3
NCOA4
YARS
CARD16
COX5A
FKBP1A
TCEAL8
CLDND1
NDUFB5
ATP5F1C
MRPL42
RPA3
MT1F
NUDT5
POLR2G
LDHB
GBP1
ATP5MF
COX6B1
COX6C
SUCLG1
TAP1
CLIC3
TPM4
GBP2
NDUFC2
CHCHD1
PPP1CA
TKT
SH3BGRL3
EIF4E2
LAIR2

MRPL51
TRAPPC1
CSK
BCL2L11
LIMD2
JPT1
GTF2H5
NDUFA12
TROAP
RTRAF
ZYX
RAC2
CHCHD5
MPG
RPS6KA1
SRSF9
CEBPD
GIMAP1
COPZ1
TALDO1
CALM3
EIF2S2
HLA-DMA
POMP
PTRHD1
COMMD4
GGCT
HAVCR2
LAP3
FERMT3
COPS9
QARS
AP2S1
ZNHIT1
ICA1
HADHB
BLOC1S1
RABL3
IFI27L2
RACK1
CD27
FKBP3
MRPL10

TXNDC9
FIBP
PTTG1
PSMD8
CXCR3
PSME1
LCP1
ACAA2
CHI3L2
NCKAP1L
PPP4C
IGBP1
PSMB3
LAMTOR2
ARHGAP30
TMEM256
COPB2
AC010618.1
AP1S1
PFDN4
NT5C3A
HMGA1
MT-CO1
PSMA2
NSMCE1
PARK7

Supplementary table 4

DE peaks	Day2 VS Day0	Day10 VS Day0	Day28 VS Day0
CD14 Mono	106	53	30
CD16 Mono	3	9	2
CD4 Naive	3	10	17
B Naive	5	7	16
CD4 TCM	5	10	20
B Memory	2	2	7
CD8 Naive	5	9	13
Treg	5	2	1
CD4 TEM	0	1	1
CD8 TEM	8	11	13
Plasmablast	6	2	3
cDC2	0	4	2
B Intermediate	0	2	5
NK	14	33	147
gdT	2	6	1
pDC	8	4	2
HSPC	2	5	6
MAIT	3	5	6
dnT	6	8	7
CD4 CTL	0	3	1
group_A	3	3	2
NK_CD56bright	1	1	1
CD8 TCM	1	1	1
ILC	1	0	2
ASDC	0	0	1

Supplementary table 5

chr
chr16-12535877-12536369
chr9-114696813-114697631
chr2-203992974-203993844
chr12-68373863-68374762
chr8-128240344-128240775
chr3-98555731-98556776
chr20-43751141-43751827
chr6-14754522-14755395
chr10-110774080-110774756
chr6-215552-216076
chr6-105646073-105646994
chr7-141557586-141559197
chr3-98563233-98565001
chr16-12532825-12533524
chr8-128313295-128314529
chr4-6180756-6181471
chr20-51480321-51482029
chr5-54881393-54881834
chr13-99289415-99290164
chr7-105808894-105809727
chr6-90472742-90474035
chr7-25862146-25862939
chr7-102713204-102714176
chrX-119647790-119648748
chr16-80569495-80570407
chr1-228794148-228794958
chr10-50508226-50508908
chr15-78049717-78050830
chr13-50236773-50237432
chr4-6855736-6856274
chr6-15023013-15023812
chr3-112672375-112673288
chr1-28199290-28200273
chr7-105848903-105850036
chr10-83680499-83680954
chr10-114801347-114801887
chr5-157540227-157542013
chr18-13290874-13291504
chr17-35889146-35891318
chr4-36285871-36286777
chr4-38128876-38129632
chr1-53413083-53413952

chr1-174153226-174153960
chr16-57609464-57610344
chr10-33117519-33118430
chr10-11863261-11864192
chr13-42405754-42406775
chr14-102819178-102820238
chr5-88203915-88204906
chr2-173354214-173355952
chr6-150987362-150987976
chr6-111837076-111838361
chr22-37162313-37163194
chr17-80891387-80892538
chr4-77586728-77587485
chr19-38559385-38560084
chr2-241867958-241869125
chr3-46280002-46280631
chr6-89297665-89299339
chr17-80720649-80721616
chr20-51404522-51405254
chr12-22409247-22410635
chr12-68119366-68120395
chr4-15771356-15771908
chr11-115222256-115223506
chr17-35879809-35880772
chr1-235998819-235999954
chr1-235873057-235873553
chr15-60389189-60389862
chr2-203994743-203995066
chr8-29990261-29991348
chr1-206769177-206769789
chr10-117653202-117653457
chr9-114690965-114691829
chr20-47487129-47488096
chr9-136538594-136540221
chr11-48011545-48012508
chr21-14533614-14535000
chr20-43736680-43737282
chr1-206784347-206784998
chr18-74994541-74995453
chr12-12384604-12385665
chr1-116762992-116764942
chr9-75152186-75152876
chr6-142842495-142843686

chr5-55036947-55038114
chr2-9702924-9704041
chr3-177198518-177199544
chr16-80580048-80580900
chr14-50521302-50522369
chr6-90402091-90402860
chr7-105806469-105807005
chr7-158656079-158656507
chr12-12430971-12431338
chr22-39910066-39911211
chr2-241762612-241764237
chr10-33133989-33134627
chr10-50417571-50418785
chr17-43251453-43252425
chr7-69803886-69804352
chr2-174752460-174753612
chr2-203870982-203871910
chr7-44623754-44624123
chr11-121481443-121482985
chr4-6198244-6199894
chr11-61060933-61061969
chr1-91548215-91549750
chr14-80959445-80960042
chr6-130222758-130224105
chr11-128471219-128472561
chr9-91071222-91071912
chr2-239304087-239304652
chr7-139043877-139044856
chr1-160755722-160757125
chr1-89607961-89609275
chr6-399756-400322
chr14-93962744-93963541
chr16-89026034-89026577
chr13-29935257-29936528
chr14-65302879-65303336
chr13-42392834-42393714
chr6-391009-393578
chr16-84593941-84594871
chr2-86785428-86786719
chr13-30739978-30740586
chr22-39090265-39091449
chr2-8050254-8050445
chr15-98952249-98953660

chr9-131668413-131669374
chr3-46285037-46285669
chr1-25099293-25100697
chr4-77595362-77595805
chr2-203914649-203915266
chr13-27130997-27131632
chr6-16731171-16732149
chr2-111633779-111634649
chr13-33016394-33017916
chr6-46011194-46012021
chr6-225018-225931
chr6-16420060-16421624
chr1-8604046-8604798
chr13-50366847-50367132
chr3-46359784-46360433
chr20-49805303-49805630
chr12-24838415-24839762
chr7-105298346-105298957
chr14-61388107-61389462
chr5-76732480-76733567
chr6-17633078-17634064
chr3-59235630-59235929
chr11-123454159-123454697
chr12-12474459-12475619
chr16-79628532-79628904
chr11-61083004-61084108
chr16-89020234-89020732
chr18-48833858-48835279
chr20-5844306-5844654
chr2-181106842-181107279
chr2-105789763-105790968
chr12-120012026-120012679
chr1-200862002-200863430
chr15-85318090-85318845
chr16-70737057-70738492
chr19-35710847-35711563
chr20-51531153-51531707
chr16-75620405-75620701
chr7-150668151-150668988
chr2-62306832-62307135
chr6-389704-390062
chr14-80930638-80931684
chr8-125607721-125608579

chr17-27471758-27472215
chr12-124774078-124775214
chr20-49669484-49670325
chr7-8125525-8126534
chr15-78046922-78047365
chr5-145946437-145946988
chr5-55061772-55062830
chr4-101020838-101021392
chr1-91555596-91556756
chr4-38306457-38306930
chr1-117654186-117654915
chr6-157143216-157143978
chr17-57982428-57982810
chr4-39353125-39354381
chr4-1201032-1202569
chr22-21766744-21767684
chr1-76270317-76270894
chr8-125125414-125126020
chr15-60649521-60650521
chr12-118053181-118053589
chr6-27866128-27867669
chr2-229822099-229822590
chrX-135213449-135213839
chr17-47829617-47830558
chr2-168194846-168195544
chr8-130244608-130245414
chr3-66472214-66473398
chr1-206737029-206737844
chr14-99252920-99253893
chr2-203948941-203949552
chr13-18211717-18212548
chr7-65798471-65798734
chr1-236415918-236416841
chr10-50527255-50527904
chr12-50532476-50533306
chr11-19240480-19241759
chr20-49668407-49669257
chr16-78742301-78743316
chr22-38985033-38985520
chr17-77783873-77784864
chr2-111705743-111706315
chr15-50260383-50260813
chr6-16699130-16699786

chr7-55569433-55570663
chr8-125514676-125515216
chr1-159076530-159077709
chr1-172674947-172675797
chr2-231425698-231427372
chr14-99243357-99243958
chr1-195195341-195195951
chr2-181163178-181163572
chr17-27466299-27467042
chr2-221517478-221518868
chr14-91228231-91229217
chr12-121671639-121673137
chr9-131406643-131408195
chr4-38132091-38132673
chr2-62332860-62333452
chr6-41531617-41532568
chr17-49341895-49343592
chr12-68159367-68160144
chr1-203649518-203650808
chr3-46947263-46948836
chr2-84938242-84940261
chr13-99367107-99368319
chr6-446581-447349
chr16-84548742-84549784
chr17-35900348-35901224
chr8-102106549-102107071
chr17-80761295-80761768
chr2-86825397-86826854
chr6-209554-210289
chr3-98546084-98546339
chr11-128304778-128305877
chr8-6417224-6418272
chr3-71086983-71088163
chr8-29408267-29408924
chr6-135087283-135089514
chr2-38665669-38667719
chr20-48760183-48761833
chr14-50000512-50002153
chr2-230981512-230982197
chr12-103505403-103505795
chr1-234314932-234315821
chr6-151373154-151374558
chr10-119704032-119705723

chr22-30706117-30706478
chr13-99380862-99381393
chr14-92768203-92768874
chr3-98557455-98557638
chr11-35247295-35248318
chr10-22681806-22684114
chr10-50431860-50432122
chr3-46284014-46284435
chr1-26378135-26378926
chr12-674073-675067
chr14-91372703-91374193
chr4-6898972-6900130
chr1-91948139-91949368
chr8-128273904-128274403
chrX-39854769-39856096
chr4-89283366-89284457
chr8-47513508-47514258
chr6-108865843-108866305
chr14-102805403-102806146
chr6-16761050-16763385
chr12-666763-667938
chr4-4268794-4270226
chr5-55023922-55024365
chr6-13375326-13375750
chr10-8416063-8417103
chr17-3796281-3797065
chr10-3809551-3810687
chr4-94214716-94215247
chr8-29349697-29351592
chr6-155223800-155224665
chr10-8061187-8062197
chr10-124688679-124689389
chr3-187802851-187803302
chr19-46954792-46956126
chr11-67267333-67268690
chrX-39860109-39860944
chr6-90397855-90398644
chr17-39769794-39770763
chr1-40241324-40242363
chr16-87979850-87980595
chr7-8131792-8132669
chrX-71622303-71623152
chrX-9676985-9677882

chr5-59098968-59099948
chr8-125930245-125931142
chr8-115427530-115428107
chr2-135954169-135954579
chr16-57041182-57042656
chr7-121530219-121531150
chr2-12500992-12502168
chr4-101019718-101020519
chr20-51400234-51400902
chr6-130259729-130261751
chr3-59425851-59426332
chr19-29623752-29624242
chr10-14659304-14660255
chr16-47544346-47544619
chr1-203652822-203653573
chr1-160437612-160438935
chr5-66840498-66841261
chr2-38696996-38698016
chr22-39116969-39118349
chr5-171957443-171958372
chr5-157025080-157025856
chr11-123358049-123358714
chr2-230973973-230974845
chr2-203859118-203859715
chr2-213015586-213016452
chr1-66247404-66248072
chr15-70300130-70301059
chr10-124611616-124613391
chr10-6491944-6492576
chr1-235876452-235876953
chr18-657185-659070
chr1-91876041-91876602
chr6-142855910-142858437
chr2-230659214-230660029
chr14-106506733-106507705
chr8-102528415-102529135
chr3-46293195-46293723
chr15-60540083-60540760
chr6-152170458-152171715
chr6-106163303-106164933
chr5-39163976-39165255
chr8-125151754-125151959
chr17-59828974-59829895

chr3-32432474-32433588
chr15-48645667-48645917
chr2-38653478-38654777
chr7-50350432-50351088
chr2-9655487-9655899
chr6-16657631-16658082
chr6-27890464-27891090
chr1-116799405-116800759
chr6-11785993-11786489
chr2-148813655-148814220
chr17-35218333-35219133
chr4-38131181-38131825
chr6-108874441-108875458
chr1-92537984-92539016
chr6-2861098-2861947
chr12-9364284-9365216
chr16-89315693-89316678
chr1-116808348-116809442
chr6-26031755-26034157
chr18-12839078-12840672
chr2-111440374-111440880
chr5-74578874-74579676
chr4-3201850-3203145
chr14-24673099-24674803
chr22-36405098-36406355
chr7-101126191-101127320
chr1-67332807-67333673
chr4-152485621-152486258
chr3-196274381-196275269
chr6-155171100-155171906
chr22-37186029-37187010
chr7-100545533-100546155
chr10-3892380-3893209
chr4-8409064-8410096
chr4-142416603-142417419
chr5-54610053-54611351
chr2-204109824-204110728
chr14-31127750-31128213
chr9-4530336-4531129
chr2-234488691-234489476
chr2-234032226-234033217
chr6-106141350-106142582
chr10-26721425-26722062

chr7-8134139-8134656
chr14-35416062-35417379
chr6-45710336-45710898
chr12-121033745-121034406
chr6-45421611-45423795
chr12-68163523-68163938
chr6-143397092-143397946
chr2-9656197-9657093
chr9-121353341-121354332
chr6-16669100-16669663
chr12-130796849-130797669
chr12-94941453-94942113
chr4-1187076-1187516
chr2-100060788-100061212
chr1-32801172-32801666
chr1-101178488-101179608
chr6-130131744-130132832
chr3-112676567-112677339
chr2-161166541-161167334
chr10-88825035-88825723
chr2-181123607-181124766
chr6-90284302-90286492
chr16-68752622-68753010
chr1-172894548-172895494
chr6-157761205-157761679
chr4-15755932-15756958
chr11-63535028-63535465
chr10-70610316-70611394
chr1-111598436-111599038
chr13-99351716-99352114
chr6-16488028-16488519
chr3-4821763-4822582
chr7-24907414-24907913
chr18-3060484-3062312
chr8-22443487-22444377
chr5-76738629-76739593
chr1-243595927-243597047
chr14-55102191-55103512
chr3-122609921-122610421
chr1-235887758-235888648
chr14-68779766-68782473
chr8-72000301-72001022
chr10-33010101-33010571

chr16-4414158-4414896
chr10-50636437-50637607
chr7-100546464-100547172
chr6-13747443-13747961
chr22-37229318-37231107
chr1-172658866-172659451
chr22-39900428-39901698
chr6-90411342-90411794
chr22-39920223-39921515
chr6-144353711-144354581
chr10-14587645-14588844
chr7-55308777-55310152
chr17-50511168-50511393
chr20-49484164-49484718
chr2-96163572-96165139
chr10-70578236-70578823
chr14-106177156-106177716
chr6-45586108-45587327
chr15-60548112-60548588
chr10-8402475-8403146
chr6-39229089-39229916
chr6-154246853-154247966
chr6-16654635-16655213
chr2-239321429-239322941
chr2-110748181-110748922
chr9-126982250-126982709
chr11-64858628-64860102
chr20-49595851-49596445
chr17-80708513-80709349
chr4-150828049-150828615
chr5-110752082-110753243
chr1-225460361-225461302
chr1-24910842-24911869
chr13-50365426-50366348
chr3-30620050-30620861
chr9-131731686-131731913
chr6-34484923-34485181
chr8-58991118-58992318
chr3-39267498-39268493
chr2-101272501-101273227
chr11-86805829-86806577
chr10-22646107-22647016
chr1-158996233-158996743

chr5-54902721-54903263
chr15-56537725-56538307
chr20-51529875-51530231
chr15-60551206-60552129
chr2-241108668-241109430
chr8-102097182-102098290
chr11-59106461-59108353
chr8-8227560-8229159
chr2-233230830-233231433
chr6-37502048-37503649
chr1-160453438-160453775
chr7-936488-936976
chr17-21098961-21100060
chr21-45227994-45228591
chr8-128184654-128184856
chr9-4653749-4654551
chrX-124342433-124343521
chr1-38029921-38030509
chr4-38177011-38178112
chr6-16700278-16701299
chr3-183349353-183350857
chr16-89097941-89098758
chr3-47037719-47038568
chr6-33426620-33427310
chr6-107712748-107713478
chr16-79278388-79279127
chr22-36356868-36358152
chr5-52743154-52744467
chr8-100409709-100410886
chr13-99307224-99308131
chr19-246868-247480
chr17-76997851-76998854
chr16-68756345-68757153
chr7-44638962-44639599
chr14-65301961-65302590
chr17-27473391-27474429
chr17-65179116-65180403
chr4-105306116-105306829
chr7-21383140-21384303
chr3-45976525-45977157
chr9-137095630-137096476
chr2-68766092-68768174
chr18-48842315-48843203

chr16-29701648-29702842
chr2-86807282-86809036
chr8-28077746-28079223
chr1-32798924-32799834
chr8-125606270-125606568
chr15-90407866-90408538
chr6-144149994-144151883
chr1-210373803-210374907
chr18-69895341-69896872
chr1-117664091-117664950
chr10-3804002-3804872
chr9-86106750-86107364
chr20-32285256-32286357
chr1-160744192-160744888
chr10-22629923-22631122
chr2-230793757-230794888
chr1-12161509-12162362
chr6-2857574-2858290
chr8-115218547-115219355
chr12-6443481-6443778
chr17-15958715-15959689
chr2-204015179-204015828
chr8-73346311-73347038
chr7-139035015-139036347
chr7-131188383-131189261
chr6-112027657-112028645
chr10-6270214-6270788
chr2-43426307-43427043
chr2-43387141-43387733
chr6-130575655-130576595
chr3-66303406-66304027
chr8-53657105-53658377
chr3-28251840-28252810
chr11-123430063-123431891
chr1-172698996-172699755
chr5-119328869-119329470
chr6-498754-499958
chr12-42299517-42300221
chr20-3906869-3907865
chr1-174204649-174205404
chr11-121650526-121650808
chr13-75489463-75490266
chr1-235855802-235856392

chr3-4850267-4851481
chr15-40743598-40744469
chr2-10620554-10620937
chr2-178193945-178194867
chr12-46214787-46215566
chr11-18246249-18247223
chr1-24954934-24955612
chr12-89468578-89469727
chr6-105458544-105459681
chr9-120891375-120893262
chr6-158289869-158291139
chr8-71843453-71844600
chr11-47934507-47935082
chr2-62208593-62209853
chr2-108612137-108612970
chr22-44709729-44711208
chr5-163461416-163462108
chr16-89360909-89361546
chr4-152524241-152524886
chr4-83235391-83236082
chr14-22536844-22537617
chr2-64325817-64326722
chr11-48019324-48019527
chr5-143008807-143009577
chr8-133568596-133571290
chr8-29229299-29229824
chr10-70579727-70580048
chr12-64668631-64670273
chr1-115641636-115643141
chr2-63977887-63978374
chr2-15176941-15177613
chr22-39076730-39077599
chr5-74631293-74632425
chr20-62922561-62922758
chr17-2070263-2071009
chr2-70068896-70069537
chr16-78794609-78795274
chr5-132019969-132020839
chr9-131678496-131679051
chr2-169362056-169365364
chr1-89957050-89957622
chr6-149147965-149148827
chr13-42377513-42378321

chr19-11095028-11095455
chr16-84514532-84515489
chr17-50903899-50904753
chr11-123068987-123070939
chr10-8360290-8360995
chr10-124718034-124718818
chr1-232457532-232458007
chr16-23482718-23483845
chr1-91523391-91523919
chr18-49168994-49169430
chr1-223716317-223717062
chr3-14142236-14143164
chr3-59451037-59452111
chr7-922597-923867
chr5-113022359-113023329
chr11-118001653-118003624
chr11-48055294-48056426
chr11-116953617-116954163
chr4-89298848-89299657
chr2-136362767-136363031
chr7-7944366-7945293
chr13-42396494-42396788
chr22-21762958-21763765
chr17-36463843-36464420
chr7-30321610-30323336
chr12-110577639-110578552
chr2-111534903-111535246
chr15-22264978-22265719
chr14-100067942-100069253
chr3-66302149-66303009
chr8-100494259-100495680
chr2-111632498-111633105
chr22-37222414-37222991
chr1-184890223-184891161
chr10-33109544-33110653
chr12-29148415-29150096
chr10-6494781-6495554
chr9-128033195-128033403
chr3-15310688-15311008
chr19-10797116-10798226
chr5-160486296-160487066
chr1-24966741-24966933
chr13-25902992-25903249

chr21-28836474-28837129
chr10-33114448-33114900
chr7-44637855-44638767
chr2-30336806-30337926
chr4-40309168-40309926
chr13-40518417-40518825
chr13-114268333-114269437
chr16-18767083-18767725
chr2-232838346-232838951
chr6-152184712-152185716
chr17-1375602-1376297
chr6-15305118-15306242
chr1-19687779-19689025
chr22-18091630-18092479
chr2-203869342-203870184
chr7-149600669-149601225
chr2-144324555-144325728
chr15-95839115-95840000
chr1-198934861-198935829
chr1-160519630-160520454
chr11-48019992-48020701
chr6-45709712-45710132
chr2-230956817-230957724
chr16-57014469-57015219
chr6-90424973-90425865
chr3-14598204-14598610
chr2-9618344-9618931
chr5-14593533-14593979
chr16-81616125-81617151
chr19-38556924-38557673
chr12-6774704-6775145
chr1-161409676-161410411
chr14-24665415-24666156
chr10-80487651-80488605
chr8-23217954-23220048
chr15-78061922-78062385
chr6-111794693-111795422
chr19-11096151-11096606
chr1-40232479-40232906
chr6-16436513-16437406
chr1-206790530-206791041
chr10-101858741-101859214
chr1-192544956-192545411

chr10-26430578-26431191
chr11-35330727-35331526
chr5-14594706-14596133
chr11-12174323-12175003
chr12-54412913-54413907
chr1-16727207-16727760
chr3-5002384-5002964
chr13-99273239-99273565
chr15-64634064-64635129
chr14-91350687-91351573
chr6-16413046-16413875
chr22-20327705-20328838
chr8-144456337-144457312
chr21-33947605-33949200
chr5-157027756-157027998
chr10-3811298-3811930
chr17-48511024-48511660
chrX-2696639-2697456
chr12-104494210-104495189
chr3-122074618-122075223
chr2-176931798-176932623
chr4-152458693-152459623
chr11-48017236-48017751
chr3-14988955-14989556
chr14-106004974-106005745
chr19-16338300-16339389
chr10-70602388-70604420
chr4-6200302-6201133
chr17-78774573-78775527
chr17-64081897-64082670
chr16-82655255-82655938
chr1-38027972-38028583
chr1-221839593-221840309
chr5-107476713-107477221
chr3-56915963-56917072
chr5-53041152-53041645
chr20-53842233-53843136
chr17-47220389-47221357
chr13-50991605-50992811
chr20-51402233-51402579
chr14-91872097-91874064
chr2-203886613-203887080
chr15-69713975-69714692

chr11-128731081-128731352
chr3-46370849-46371094
chr6-16659338-16660340
chr14-89616782-89618228
chr10-119029058-119030963
chr1-168408946-168409309
chr15-65512829-65513351
chr17-14205951-14206216
chr2-224523276-224524851
chr6-111750880-111751763
chr7-150663921-150664160
chr9-131627520-131628920
chr17-62064069-62064355
chr20-34620733-34621102
chr1-35505876-35507473
chr10-13340134-13340950
chr6-15300535-15300969
chr10-94544973-94546575
chr15-64246783-64247050
chr7-930034-930398
chr20-38874873-38876031
chr5-39209754-39210527
chr5-157214811-157216105
chr11-11620936-11622427
chr3-122030371-122030998
chr6-111823715-111825505
chr14-91349304-91350046
chr1-20185067-20186768
chr3-144128179-144128727
chr4-38160400-38161457
chr8-25319391-25320045
chr10-119120210-119120830
chr20-48769115-48769922
chr1-199941812-199942320
chr11-122850031-122850589
chr2-201123836-201124696
chr9-134301647-134302154
chr14-54435497-54435975
chr22-30710116-30710431
chr1-228765539-228766014
chrX-71606656-71607413
chr9-92963741-92965527
chr6-13427519-13428777

chr16-79271897-79272755
chr6-5302319-5303603
chr6-142907065-142907977
chr20-49534213-49535366
chr6-139146453-139147027
chr20-50338545-50339354
chr15-94325036-94325563
chr3-11306867-11307475
chr1-172708307-172709023
chr1-160640449-160640760
chr15-69468190-69469224
chr1-237411553-237412139
chr16-57601775-57602574
chr1-26799602-26800845
chr5-157198359-157198958
chr10-110085738-110087015
chr2-37599489-37600410
chr6-15326126-15326476
chr17-74282594-74283424
chr17-67434004-67436049
chr6-144305807-144306492
chr20-58982799-58983184
chrX-124063794-124064630
chr5-119353795-119354333
chr15-62390011-62392151
chr17-48505727-48506428
chr16-48611675-48612276
chr6-26232791-26235196
chr22-26642790-26643839
chr20-10568001-10568404
chr12-130764086-130765505
chr8-134324560-134325153
chr6-45420674-45420885
chr14-93103454-93104206
chr6-188739-189358
chr2-230660541-230662037
chr7-25967998-25969008
chr19-38864403-38865871
chr16-88619630-88620748
chr1-19476535-19478643
chr1-150567255-150569354
chr15-90096802-90098110
chr1-23110540-23111276

chr2-179095922-179096760
chr2-203950928-203951962
chr6-11366536-11367804
chr1-40185715-40186877
chr1-32255407-32255946
chr6-158567625-158568489
chr2-239602167-239602845
chr4-3192196-3193102
chr2-234490105-234490897
chr8-29680896-29681339
chr7-139085134-139085888
chr10-110356039-110357189
chr11-62555363-62557101
chr16-3577951-3579460
chr7-43669130-43670028
chr6-111767633-111768251
chr15-55277081-55277575
chr5-160467435-160468486
chr12-122166523-122167327
chr9-120905380-120906169
chr17-59845769-59846662
chr21-45224484-45225071
chr6-142819400-142821112
chr2-197293297-197294646
chr9-79630794-79631331
chr3-36912125-36913082
chr15-91491236-91491736
chr22-44652349-44653288
chrX-71618295-71618790
chr12-120013581-120014350
chr16-11557043-11557729
chr5-107474615-107475682
chr3-106338313-106339264
chr2-48362376-48362802
chr2-98438625-98439258
chr20-43840448-43840969
chr17-40560195-40561297
chr14-106184518-106185421
chr3-123492077-123492537
chr8-42177259-42178109
chr3-59424436-59424959
chr17-47703166-47703728
chr8-29995091-29995463

chr4-6177680-6178180
chr1-184745778-184746323
chr9-134065818-134066326
chrX-1534372-1537849
chr11-122843184-122844130
chr2-86860715-86862913
chr17-40515319-40516169
chr8-127977694-127978225
chr8-128554849-128555809
chr11-2387349-2388016
chr3-132034564-132035396
chr17-32523164-32523712
chr22-22787821-22788718
chr20-51541005-51543028
chr4-121199713-121201215
chr1-236394908-236396071
chr1-9800153-9800684
chr6-158717393-158718210
chr5-157544519-157545106
chr21-45226646-45227147
chr2-144510386-144511022
chr9-131709760-131710616
chr13-24618093-24619141
chr4-101039173-101039711
chr2-68379220-68380001
chr16-48608498-48608828
chr3-66480657-66482109
chr4-89294342-89295212
chr8-664870-665238
chr2-190695672-190696322
chr22-39173881-39175045
chr8-47602520-47602733
chr8-133068220-133069047
chr20-50110059-50110625
chr1-198101660-198103103
chr12-75943947-75945011
chr1-67274655-67275091
chr3-27900233-27900914
chr5-159412088-159412919
chr2-241863433-241863929
chr3-106068537-106069249
chr14-98173201-98174378
chr19-4214532-4215116

chr11-48013030-48013939
chr3-27905603-27906012
chr15-90099118-90099370
chr3-152323549-152324258
chr1-24971185-24971969
chr7-130960056-130961748
chr14-91355073-91356745
chr11-86047109-86048053
chr5-157607616-157608183
chr15-83446870-83447642
chr18-58615186-58616238
chr2-203867492-203868010
chr13-99275049-99275335
chr13-84136506-84137759
chr16-87762144-87762566
chr6-154229627-154230311
chrX-41556951-41557297
chr13-40653318-40654194
chr3-112514555-112514903
chr8-141244809-141245867
chr17-77459030-77460455
chr19-10566657-10567108
chr1-172638710-172639793
chr16-87764570-87766468
chr11-128324229-128325308
chr18-24000463-24001969
chr8-100430559-100431412
chr3-46348933-46350486
chr1-53765771-53766091
chr13-102798612-102800841
chr15-52464367-52464825
chr3-106184965-106185481
chr10-102189062-102189884
chr20-51666924-51667598
chr20-51494211-51494891
chr6-111764343-111765034
chr14-105986475-105987217
chr22-28810569-28812506
chr7-142796506-142799412
chr1-184866767-184867799
chr10-3960709-3961715
chr4-40305335-40305725
chr3-187367795-187369364

chr3-111238913-111239376
chr17-14201006-14202289
chr3-106176493-106177350
chr2-197062474-197063223
chr22-31524147-31524566
chr5-66828344-66829396
chr1-203655741-203656168
chr10-22649356-22649804
chr3-42638155-42639553
chr13-30147570-30149037
chr8-47600564-47601338
chr2-161952676-161953276
chrX-1462649-1463475
chr5-119287353-119288153
chrX-10082730-10083611
chr15-77013673-77014894
chr17-67244761-67247464
chr1-76294489-76295411
chr16-87865979-87866443
chr9-132451110-132451760
chr10-69228775-69229846
chr9-124868508-124870706
chr17-2259460-2260483
chr9-128026006-128027498
chr5-55022329-55022825
chr1-10510615-10511640
chr22-37807506-37808310
chr4-101327958-101328901
chr6-105644147-105644533
chr21-33949647-33950157
chr7-8176006-8177464
chr2-234290436-234291269
chr21-38766844-38767765
chr1-7930563-7931708
chr2-135948473-135949067
chr2-196291656-196292782
chr10-70162314-70163285
chr6-110944517-110945189
chr7-150665907-150666311
chr1-84971999-84972829
chr22-39097162-39097450
chr14-91397220-91398079
chr4-89298154-89298561

chr10-71726130-71726503
chr6-45663488-45664158
chr19-15467044-15467348
chr2-230877450-230878427
chr1-230208578-230209343
chr17-80876616-80877704
chr19-49567785-49569408
chr3-122091480-122092927
chr17-1823283-1824103
chr20-62739599-62741053
chr3-11624131-11625201
chr10-113960562-113962090
chr2-136382006-136382807
chr6-501875-502506
chr16-27449580-27450430
chr15-60582425-60582723
chr5-112923189-112924165
chr2-173149625-173150284
chr14-65637719-65638269
chr10-14584712-14585006
chr5-156920359-156920960
chr14-24692833-24693595
chr22-25067784-25069024
chr2-233233773-233234635
chr1-117655959-117656786
chr6-16651631-16652390
chr6-90080629-90081582
chr19-8575478-8576123
chr6-13355301-13356601
chr15-69755515-69755996
chr5-14809473-14810372
chrX-124346093-124347550
chr2-96156283-96156803
chr6-142845024-142846193
chr16-79283602-79284019
chr8-93904100-93904673
chr8-142844668-142845236
chr7-35652085-35652491
chr1-184840399-184841091
chr17-3710723-3711537
chr2-230781328-230782084
chr19-6592538-6592963
chr3-37430757-37431492

chr16-29600099-29601087
chr2-239548597-239548854
chr14-94056552-94057310
chr19-47541529-47542239
chr7-38232339-38233519
chr4-101418033-101418849
chr2-234291778-234293035
chr10-5975854-5978268
chr5-126449626-126451172
chr15-39623350-39624157
chr12-6532346-6533007
chr20-44646130-44647122
chr7-44635380-44635600
chr10-73758916-73760029
chr10-6927427-6928971
chr5-134111857-134112103
chr2-98835712-98836646
chr1-198519591-198521111
chr19-13838926-13840720
chr16-50267439-50267943
chr10-333377-334286
chr3-59515041-59515484
chr13-20419562-20420106
chr3-59443733-59444915
chr1-68496113-68497603
chr14-21263798-21264576
chr5-68711239-68712155
chr7-36787868-36788524
chr17-35341334-35342055
chr17-40607631-40608669
chr2-204026937-204027584
chr19-45016176-45017335
chr5-179836443-179837347
chr16-31421659-31422108
chr7-70679445-70680142
chr21-28837495-28837909
chr15-50181989-50183072
chr14-72476578-72477321
chr12-10394558-10396015
chr14-99286761-99287878
chr6-155050354-155050725
chr14-24694763-24695753
chr12-47215401-47217057

chr17-45312088-45312380
chr15-99104784-99105990
chr10-62047906-62048386
chr1-8829321-8830025
chr6-106125848-106126693
chr11-48001271-48002235
chr5-119340384-119342413
chr13-114339786-114340630
chr2-36240205-36241261
chrX-3707745-3708299
chr22-39094756-39095007
chr15-67102048-67102703
chr5-157252674-157253813
chr21-46559102-46560137
chr16-82653651-82654406
chr6-128258996-128260553
chr2-10493363-10494128
chr1-203764568-203765891
chr17-32519199-32520627
chr11-12195793-12196654
chr6-16956350-16956922
chr22-39080362-39080803
chr9-130038020-130038628
chrX-1657413-1657939
chr6-127934137-127935019
chr4-2511041-2512280
chr16-31364910-31365591
chrX-65723611-65724227
chr22-24409817-24410667
chr4-39031834-39032989
chr2-86790795-86791803
chr6-16729317-16729694
chr15-78047778-78048161
chr2-239150367-239151205
chr5-156922149-156922779
chr2-203718098-203718530
chr3-42555806-42556839
chr16-89925830-89926750
chr1-89285625-89287538
chr14-91229535-91230503
chr19-29622071-29622806
chr2-179031627-179032701
chr1-91731348-91732875

chr9-89426344-89427018
chr1-111219748-111220076
chr4-121696107-121697937
chr12-22334522-22336046
chr1-205479968-205482071
chr1-160682949-160683815
chr1-12201437-12202121
chr7-140223688-140224283
chr7-131231205-131231915
chr8-93700001-93701181
chr17-40540357-40541999
chr16-28140830-28142538
chr10-61961325-61962365
chr19-6590310-6591329
chr4-153521354-153523014
chr2-111483050-111483602
chr18-61948889-61949549
chr19-19451366-19452650
chrX-30712954-30713567
chr22-42316929-42317557
chr17-59837177-59838132
chr13-99355903-99357528
chr15-48962929-48964481
chr9-132908478-132909111
chr20-1664786-1665637
chr10-3067104-3069214
chr16-70520816-70521004
chr16-57592466-57593303
chr12-669049-669425
chr5-88251047-88251457
chr2-239288888-239289897
chr1-235104057-235105570
chr20-37043222-37044547
chr19-10104645-10105292
chr15-67240206-67241649
chr15-52220866-52222344
chr14-63477967-63478870
chr2-136057006-136057660
chr16-8958374-8958685
chr7-106346450-106347331
chr22-37163891-37164601
chr12-107368632-107369274
chr10-6542961-6544020

chr2-135819811-135820644
chr15-31434600-31436377
chr17-66548065-66548701
chr14-91260958-91261669
chr4-153487546-153489371
chr5-131336710-131337965
chrX-134718596-134719224
chr4-38310381-38311063
chr10-124722993-124723721
chr2-8346011-8347061
chr1-30743485-30744606
chr7-43650658-43651565
chr11-60915258-60915933
chr20-63728421-63728810
chr19-39351316-39351987
chr21-42238469-42238880
chr6-111758899-111760536
chr1-15963913-15964287
chr6-105648231-105648540
chr3-96813063-96814046
chr5-126758230-126759090
chr5-139458624-139459396
chr5-172178788-172179936
chr1-192571811-192572159
chr17-40480137-40481180
chr11-6319493-6321149
chr14-90060240-90061216
chr20-20603088-20603956
chr10-8365985-8366508
chr2-70108546-70109662
chr10-17026779-17027305
chr13-45398905-45399364
chr17-80780198-80781532
chr1-51520682-51521145
chr10-49762149-49762520
chr11-35257098-35257797
chr12-22396124-22397195
chr2-223760713-223762051
chr2-202266659-202267114
chr5-157575080-157576267
chr3-28326243-28326948
chr13-26557254-26557918
chr9-97038809-97039624

chr8-134278126-134278726
chr12-105330044-105332116
chr12-4142736-4143503
chr15-64911316-64912111
chr6-34656101-34658166
chr3-46097784-46098915
chr19-51395289-51395742
chr1-168520117-168521106
chr15-50262161-50263106
chr6-150865373-150866889
chr22-41939947-41941607
chr11-128290001-128291107
chr22-37306802-37308206
chr14-97702925-97703531
chr20-51525174-51526245
chr2-223941617-223942167
chr1-226723782-226724712
chr17-36082120-36083195
chr6-24974078-24975084
chr12-110727794-110728391
chr9-73153902-73155332
chr9-133133451-133134588
chr3-52892833-52893216
chr3-156483458-156484907
chr8-38359291-38360139
chr5-50593571-50594324
chr15-90142628-90143611
chr1-202162091-202162437
chr8-38465416-38467182
chr2-12412849-12413306
chr14-105858874-105860292
chr19-46786102-46786960
chr11-85827602-85828148
chr16-53534901-53535289
chr18-77101269-77102875
chr17-47217211-47217615
chr7-92809476-92810482
chr3-196179406-196180354
chr6-45619986-45620510
chr21-25572891-25574199
chr10-79285416-79285623
chr4-26075585-26076626
chr3-36876123-36877128

chr12-21456226-21456984
chr18-79245528-79246829
chr16-81841650-81844097
chr1-161541946-161542383
chr3-194326870-194328486
chr18-13266117-13267493
chr10-17443892-17444687
chr20-24948731-24949998
chr15-63497101-63498490
chrX-1468269-1468803
chr8-143549347-143549709
chrX-124212636-124213008
chr2-62304536-62305376
chr9-91796655-91799044
chr5-145941162-145941969
chr2-110858216-110859348
chr2-230411215-230412625
chr6-152636056-152637172
chr9-121071649-121072436
chr11-128551588-128552344
chr15-44545814-44546696
chr3-171248325-171249278
chr10-124696491-124697224
chr20-10350690-10351925
chr8-129971493-129972464
chr8-134332743-134333603
chr5-139389694-139390340
chr13-112768869-112769304
chr2-102366759-102368135
chr5-72194739-72195242
chr20-10446902-10447358
chr11-118007149-118008688
chr7-8198078-8198787
chr11-3946799-3947729
chr22-37896155-37896838
chr19-18088548-18089567
chr12-66303548-66303912
chr16-89409074-89409727
chr11-88353871-88354474
chr22-44455146-44456116
chr5-142783324-142784652
chr7-139100309-139101275
chr1-101081092-101082029

chr14-106013761-106014271
chr3-27880948-27882110
chr10-23362940-23364087
chr2-168211004-168212499
chr6-53312335-53312896
chr9-91950022-91950662
chr19-54210694-54211387
chr13-46679322-46680304
chr12-104462845-104463873
chr2-86079835-86081071
chr6-20365413-20367377
chr15-85691492-85692042
chr15-60397109-60399295
chr1-6600977-6601603
chr3-12003784-12004584
chr4-101334818-101335493
chr7-603643-604987
chr22-35648047-35649424
chr14-91249910-91250493
chr10-17511182-17511876
chr9-89437075-89437954
chr1-198918983-198920463
chr16-80590964-80591818
chr8-67505912-67506518
chr2-102368502-102368943
chr6-15296721-15296997
chr9-131662054-131662438
chr11-12087210-12088420
chrX-124239382-124240441
chr1-116769129-116770976
chr1-46549501-46550266
chr21-25563378-25563933
chr2-234026660-234027153
chr10-133388775-133390371
chr8-206536-207266
chr1-25642181-25643128
chr15-77012787-77013374
chr10-50512911-50513466
chr6-16503880-16504890
chr2-24919135-24921108
chr12-9670847-9671728
chr1-248924635-248925512
chr1-206470176-206471557

chr4-145125921-145126658
chr5-73693534-73694850
chr6-90103277-90103814
chr22-26592409-26592839
chr5-126747865-126748454
chr2-136327108-136327927
chr15-85625490-85626352
chr15-60840619-60841511
chr2-203763436-203764348
chr5-55072557-55073431
chr11-117969466-117969881
chr1-224497703-224498833
chr4-40672552-40673579
chr3-71421646-71422861
chr6-135624149-135625174
chr7-111012808-111013730
chr19-51372623-51373397
chr9-36470694-36471368
chr6-26054738-26056945
chr2-239274697-239275695
chr6-111803439-111805309
chr6-16704380-16704964
chr22-40240770-40241777
chr8-20251515-20252408
chr4-121706406-121707317
chr8-115429187-115429596
chr5-76714682-76716913
chr15-60541861-60542509
chr5-60843946-60844513
chr7-44631064-44631762
chr14-93959298-93960068
chr10-69045308-69046132
chr4-2936138-2936324
chr13-24625442-24626925
chr17-35895878-35896672
chr17-78816953-78817716
chr3-46959009-46959770
chr1-200129199-200130087
chr6-235862-236524
chr14-71555177-71556438
chr4-37890475-37892077
chr1-24964682-24965662
chrX-19747420-19748426

chr20-49700664-49701694
chr8-95099876-95101115
chr2-156480118-156480894
chr12-12736645-12737567
chr11-66327851-66328891
chr1-221829263-221830152
chr6-111793567-111794369
chr7-70647789-70648829
chr22-39040329-39041216
chr9-114681470-114682350
chr3-10551901-10552845
chr17-82882555-82883255
chr2-230441294-230442108
chr9-76577404-76578138
chr1-161626522-161627082
chr1-31932609-31932842
chr16-87963248-87963878
chr10-8056977-8057315
chr5-119308434-119309767
chr8-127976575-127976953
chr16-56999231-57000903
chr2-61670456-61671496
chr10-80472116-80472581
chr1-156920292-156920844
chr12-68080553-68080933
chr11-78464222-78465918
chr2-9641656-9642177
chr3-157066935-157067756
chr12-56683671-56684430
chr1-169692454-169693537
chr13-114142350-114144001
chr11-85750714-85751025
chr5-102754323-102756423
chr5-40437455-40438099
chr1-32627426-32628237
chr10-102241427-102241937
chr2-105797115-105797813
chr5-132456270-132457977
chr10-43228663-43229921
chr6-16319707-16320534
chr9-21919453-21920344
chr19-10963436-10963833
chr20-58408040-58409163

chr3-187798275-187799467
chr11-14414691-14415663
chr9-87276224-87277297
chr19-10112013-10113458
chr2-48343723-48344667
chr16-88456115-88456455
chr10-124650318-124651399
chr2-8425099-8426124
chr14-65927678-65928525
chr14-74617915-74618938
chr20-43914401-43916290
chr1-24915480-24916034
chr12-68673672-68674162
chr3-134794829-134795747
chr5-55096056-55097402
chr20-433300-433978
chr13-40388557-40389736
chr7-5143024-5143500
chr16-56938367-56940186
chr7-25856627-25857473
chr6-111487791-111488001
chr10-3874733-3876218
chr2-108609027-108609465
chr14-71142353-71143780
chrX-104110436-104111223
chr15-49235459-49236059
chr3-71050359-71051159
chr8-25322595-25323291
chr4-2570579-2571374
chr1-6358705-6360191
chr1-204452850-204453624
chr2-85704804-85705392
chr8-58360958-58361486
chr2-161384792-161385297
chr7-140358444-140359694
chr2-105855785-105857665
chr17-27569185-27569806
chr5-55101515-55102915
chr9-92440533-92441063
chr3-30655018-30655916
chr6-152634708-152635311
chr14-98198151-98198798
chr9-89422219-89422465

chr10-79347005-79348492
chr1-117661023-117661455
chr4-39812345-39815058
chr14-81485264-81485717
chr8-140590904-140591891
chr6-27814251-27815488
chr12-124992869-124994958
chr18-13279116-13279879
chr3-11784462-11785469
chr12-117968811-117969496
chr12-64679537-64680177
chr2-174750817-174751435
chr3-32428172-32428969
chr1-15949347-15950197
chr6-37507995-37508601
chr9-120925923-120926867
chr20-36644201-36644990
chr12-50221365-50222975
chr8-133066763-133067353
chr1-24997355-24998065
chr11-133082780-133083380
chr20-49676504-49677468
chr1-160428759-160430243
chr19-38683536-38684718
chr11-72519365-72520519
chr10-124734237-124735167
chr8-122909934-122911447
chr8-133054813-133055086
chr8-127992706-127993771
chr17-74279997-74280579
chr11-12673802-12674830
chr5-111229952-111230754
chr16-89357419-89358919
chr17-40516539-40517349
chr11-32829264-32830618
chr1-24922337-24922589
chr19-2081821-2082528
chr9-14322158-14323114
chr12-12467294-12468172
chr3-46955794-46956298
chr5-40441634-40442634
chr1-224623471-224624524
chr20-63902721-63903303

chr12-77065053-77066185
chr3-69012855-69014202
chrX-124395180-124396080
chr16-4142242-4142783
chr3-183555302-183556201
chr10-124719028-124719743
chr15-31346408-31346852
chr2-70132346-70133152
chr10-69089777-69090592
chr5-132426417-132428179
chr4-40200028-40201255
chr1-12047106-12047983
chr12-39331610-39332319
chr2-127829617-127830395
chr8-96282796-96284130
chr15-31387199-31388078
chr15-63939574-63940106
chr9-133141874-133142584
chr3-69197865-69198660
chr3-4834338-4834828
chr8-81280065-81281678
chr12-96500863-96502065
chr19-8568880-8569770
chr4-15962892-15963915
chr4-109690498-109691770
chr10-124732796-124733479
chr20-38872956-38873781
chr3-45950445-45951534
chr17-40531233-40531957
chr4-38523661-38524463
chr3-128112411-128113255
chrX-1471768-1472379
chr20-57618905-57621225
chr1-64922365-64923779
chr16-89723565-89724161
chr2-201130590-201131303
chr3-27840746-27841214
chr22-26657102-26657908
chr7-50331702-50332236
chr4-154743442-154744406
chr16-28309762-28310543
chr11-118240672-118241984
chr5-156913443-156914636

chr19-7186147-7186685
chr1-25064582-25066587
chr16-80803775-80804927
chr5-157195989-157196349
chr10-62853031-62854220
chr13-99322099-99323298
chr11-67408570-67409879
chr20-5911149-5912018
chr15-64009696-64010207
chr17-3794997-3795763
chr19-39297911-39298643
chr7-925696-925950
chr10-119090402-119091571
chr17-63598267-63598624
chr15-64247998-64248462
chr1-24923608-24923854
chr22-37149640-37150513
chr21-35045171-35046280
chr16-57128671-57129444
chr14-72594202-72594954
chr19-41871529-41872125
chr19-38636737-38637457
chr8-27837257-27838316
chr1-229253164-229253907
chr4-26197228-26197933
chr5-80755577-80756439
chr6-148355-148969
chr8-128076759-128077283
chr3-101518198-101518936
chr15-60549723-60550173
chr21-42959009-42959377
chr12-3728020-3729190
chr3-30643899-30645092
chr8-74350107-74350991
chr1-24985011-24985554
chr2-121699203-121700156
chrX-39869922-39870856
chr22-49825880-49828155
chr7-30347672-30348713
chr1-8473387-8473909
chr11-36375974-36378415
chr14-75514217-75515998
chr12-132818399-132818947

chr3-157128247-157130836
chr5-169650973-169651429
chr7-924772-924943
chr17-47204075-47206041
chr14-22549835-22550631
chr10-71742139-71743209
chr8-70141201-70142636
chr2-32946200-32947110
chr8-142841695-142842358
chr9-127970101-127971773
chr16-2968771-2970397
chr10-62383575-62385002
chr11-60978610-60979499
chr3-152100512-152100978
chr16-26597974-26598630
chr21-33975394-33976990
chr6-45677405-45678075
chr4-40238047-40239402
chr4-1176830-1177900
chr12-47229394-47230119
chr15-67085302-67086330
chr4-82798225-82799776
chr2-167292439-167293722
chr2-7725179-7726125
chr2-234025234-234026322
chr1-42931089-42932662
chr3-160801408-160802335
chrX-72094771-72095462
chr19-1883952-1884668
chr17-30363512-30364304
chr12-96445500-96446574
chr19-1062827-1063412
chr9-132486840-132488065
chr6-75496189-75496772
chr2-86793702-86794161
chr12-6533575-6535652
chr15-77021663-77022513
chr11-65421219-65421687
chr7-29255939-29256352
chr6-127950600-127951958
chr12-122898404-122899054
chr16-30472197-30472973
chr4-1330019-1331287

chr11-118230046-118230370
chr2-180590972-180591614
chr2-134288701-134289685
chr10-84356879-84357277
chr3-189049473-189050200
chr7-7815920-7816695
chr10-3757505-3758585
chr8-115222647-115223344
chr20-48818373-48819070
chr14-95523188-95524431
chr8-100386053-100386788
chr10-8053298-8055189
chr22-39908771-39909033
chrX-45758325-45759527
chr17-47735562-47735898
chr14-22531183-22532107
chr1-233294970-233295914
chrX-134549185-134551264
chr6-13383290-13384136
chr20-63134519-63135658
chr1-235641943-235643026
chr2-7437238-7438492
chr1-91753280-91754737
chr16-15389757-15390349
chr6-27823748-27825144
chr8-101902425-101903318
chr5-157186587-157187678
chr1-159008677-159010523
chr10-100370576-100371598
chr12-673014-673575
chr6-45155947-45156467
chr4-26873064-26874044
chr16-67967274-67969508
chr14-39229019-39229815
chr5-132102187-132102686
chr3-189132126-189133039
chr6-2869044-2869897
chr2-8515054-8516267
chr20-51528635-51528982
chr1-121365295-121366251
chr10-131904572-131905022
chr16-69443761-69445244
chr7-21355231-21355995

chr16-88637095-88637685
chr11-796015-796738
chr6-46174767-46175161
chr12-44847595-44848940
chr1-172662524-172663313
chr6-16712346-16713706
chr2-144454631-144455126
chr17-47740766-47742109
chr2-171319031-171319698
chr6-31203357-31204515
chr2-62313697-62314520
chr19-38681030-38681649
chr12-104682578-104683350
chr12-68364834-68365738
chr6-33620217-33622011
chr3-18740434-18741755
chr1-89125744-89126414
chr18-107878-110780
chr1-111592542-111593477
chr19-46936450-46937122
chr12-79927790-79928670
chr15-31347167-31347940
chr1-184386490-184388686
chr1-156813772-156814430
chr12-9669552-9669883
chr11-86182929-86183968
chr2-96131419-96131924
chr1-116738562-116739377
chr1-24924952-24925648
chr10-11178340-11179277
chr7-139091209-139091441
chr22-36331238-36333308
chr6-14452521-14453538
chr8-133693968-133694732
chr19-4914870-4915459
chr14-61385102-61385824
chr16-56657196-56658393
chr8-97980040-97980523
chr7-130941430-130941729
chr6-137707616-137708883
chr15-39624638-39625361
chr12-14396108-14396402
chr17-4374967-4375861

chr3-30630800-30632021
chr20-44019424-44020334
chr12-107178644-107179376
chr17-68184677-68185299
chr20-33369274-33369915
chr1-24992335-24993195
chr10-13116073-13116947
chr14-61335855-61336290
chr2-197240148-197241092
chr17-82863766-82864730
chr22-37638472-37640051
chr4-56048525-56050002
chr3-123884081-123884788
chr22-38981908-38982616
chr12-6337138-6337681
chr8-133046357-133046882
chr1-10498246-10498528
chr2-241870983-241871739
chr6-140809489-140810153
chr1-174964270-174965856
chr20-51424281-51425077
chr10-28668580-28669200
chr22-49867680-49868392
chr15-60004958-60006825
chr5-170278524-170279578
chr11-122743660-122744532
chr2-237694467-237695596
chr14-73711991-73713056
chr1-198668906-198669419
chr5-35810437-35811849
chr22-36383258-36383494
chr9-114718704-114719301
chr21-33936492-33938332
chr2-15316296-15317013
chr14-91113635-91115653
chr11-67271356-67272100
chr16-56625079-56626211
chr12-76020149-76021472
chr1-23118835-23119662
chr18-59899378-59901148
chr1-67307139-67308069
chr14-24605513-24605859
chr1-210332261-210333236

chr14-100066020-100066529
chr12-93436142-93436746
chr2-105744392-105746937
chr3-106205864-106207067
chr17-49715113-49716383
chr1-54800818-54801865
chr6-111843587-111844141
chr1-89610297-89611100
chr6-149031865-149032867
chr14-73741456-73742623
chr9-120897077-120897519
chr4-40205185-40205940
chr22-36329137-36329609
chr2-157439492-157440096
chr15-22796624-22797355
chr16-48612725-48613644
chr5-129903964-129904974
chr2-196200389-196201458
chr3-186930111-186932275
chr13-32530710-32531982
chr21-14522943-14523781
chr17-32523978-32524535
chr9-127423804-127424996
chr19-10101991-10102547
chr12-8644197-8645382
chr20-44643569-44643988
chr14-100078591-100079543
chr6-106194941-106196118
chr9-109320830-109321447
chr6-36120281-36121599
chr6-13453847-13455308
chr6-24935430-24937144
chr3-46372449-46372740
chr6-26249595-26250959
chr15-55281970-55282866
chr8-47597952-47598490
chr1-159012548-159012954
chr16-29094324-29095566
chr15-78033975-78035716
chr14-99240716-99241942
chr15-55279666-55280326
chr17-77843360-77844293
chr17-44655910-44656655

chr7-50266014-50267708
chr6-135094174-135095102
chr1-29230543-29231373
chr17-43211403-43213008
chr12-24902272-24904171
chr17-80265515-80265893
chr9-33130455-33131143
chr15-89243442-89244638
chr18-69885613-69886595
chr20-59159246-59160049
chr12-11650859-11651358
chr11-118342462-118342995
chr16-88780935-88782174
chr1-243421329-243422302
chr20-59177545-59178382
chr6-36912035-36913212
chr3-30640036-30640295
chr6-111805533-111806106
chr2-8310581-8311278
chr19-19661905-19662174
chr7-50329704-50330960
chr5-60699489-60700667
chr14-91315716-91316456
chr12-10285881-10286690
chr9-110047792-110049911
chr3-46108980-46109769
chr17-35244282-35244857
chr1-230426171-230426949
chr11-123087575-123088173
chr20-51532993-51533266
chr9-133130822-133131316
chr14-101823173-101824058
chr13-50255691-50256946
chr1-67336704-67337186
chr8-38184517-38184986
chr2-12708827-12709753
chr1-31943770-31945027
chr2-241858706-241859274
chr3-39234508-39235225
chr10-12365831-12366616
chr15-40765089-40765836
chr7-26166645-26167769
chr22-43086158-43086413

chr9-76655043-76655588
chr16-81715172-81716138
chr12-51970955-51971466
chr15-85692615-85693497
chr1-160558747-160560759
chr7-128320176-128321333
chr2-69648180-69648789
chr17-68180382-68181723
chr16-47142979-47144577
chr16-16025154-16026171
chr19-290081-290653
chr11-61023568-61023987
chr1-10494609-10495696
chr6-106525121-106525522
chr5-146765209-146765840
chr4-39932796-39933433
chr3-138780812-138781622
chr15-94305025-94305499
chr6-127971626-127972662
chr20-43959091-43959898
chr2-144505422-144505902
chr1-206828982-206830258
chr7-43625230-43626403
chr14-50362607-50364276
chr12-110688779-110690130
chr15-99014712-99015694
chr1-3889976-3891345
chr6-5173880-5175195
chr7-157302114-157302921
chr1-160742492-160742802
chr6-15299216-15300030
chr2-171521998-171524184
chr6-21438265-21439322
chr6-193809-194342
chr13-23979494-23980645
chr1-2554990-2557123
chr2-222423640-222425584
chr17-63522818-63523577
chr16-29626678-29628146
chr5-170266802-170268198
chr7-37342420-37343536
chr3-27721033-27722642
chr1-147084170-147085383

chr5-94793123-94794146
chr22-45129716-45130272
chr1-20932058-20933064
chr18-3511720-3512543
chr18-48868347-48869506
chr21-46424001-46426001
chrX-136935421-136936093
chr4-83217435-83218405
chr8-102585109-102586309
chrX-12984625-12985620
chr11-116853365-116854245
chr22-48097848-48099105
chr2-8543294-8544272
chr6-45830457-45831730
chr1-234601424-234601910
chr16-84738154-84738931
chr2-53786212-53787831
chr2-98460766-98461428
chr9-131667654-131668005
chr22-36334199-36335229
chr17-8161990-8162440
chr1-230126966-230127328
chr7-100219782-100220326
chr15-93141363-93141913
chr6-42357275-42358117
chr2-37625966-37626695
chr6-107716004-107716243
chr4-142405017-142407488
chr1-35769253-35770301
chr14-99260945-99261461
chr10-27224831-27226477
chr16-4474344-4474827
chr2-161956468-161957097
chr15-84761438-84762167
chr5-132663125-132664515
chr12-7989852-7990553
chr6-13485975-13488543
chr5-39176849-39179363
chr20-5039100-5039866
chr5-71600944-71601971
chr8-128540427-128541376
chr16-56643105-56643938
chr3-27714725-27715503

chr12-51318681-51319412
chr2-98472870-98474446
chr7-100351805-100352888
chr17-40326217-40326422
chr2-168192897-168193735
chr5-151078819-151079033
chr4-152696756-152697524
chrX-65750456-65751305
chr11-68340402-68341313
chr1-84996736-84999313
chr14-100058713-100059146
chr4-6690263-6690855
chr10-17669391-17670077
chr1-89719544-89719975
chr17-3763358-3763719
chr19-49560564-49560889
chr3-161310523-161311589
chr6-106114611-106114899
chr17-17179376-17179793
chr5-119367516-119368940
chr7-156640125-156641419
chr4-184417080-184418256
chr6-129748248-129748934
chr7-74093015-74095112
chr7-50333241-50333687
chr9-89418276-89419534
chr2-87476292-87477452
chr6-157460800-157462047
chr11-117230229-117230994
chr16-87853580-87854286
chr20-62977531-62978433
chr6-2940012-2940860
chr8-25385009-25385650
chr5-171985007-171985576
chr12-31721810-31722718
chr10-68471354-68472325
chr1-10524974-10525873
chr5-40386208-40386677
chr16-80656373-80657131
chr6-137588996-137589946
chr17-47734587-47735033
chr20-63730472-63731376
chr2-38722190-38723027

chr3-177538483-177539797
chr3-183427910-183429178
chr16-30350262-30350853
chr4-184420430-184421417
chr14-22561636-22562556
chr12-9760771-9761754
chr11-82955747-82956554
chr12-104671271-104672238
chr17-14069030-14070040
chr13-99382660-99384910
chr6-16711645-16712145
chr6-107726036-107727016
chr9-130128122-130129576
chr2-68203476-68204170
chr7-102415465-102416350
chr15-94272176-94273225
chr12-89494413-89495143
chr6-26271139-26271790
chr7-121442884-121443143
chr6-137269298-137269977
chr9-5851044-5851854
chr2-239363814-239364615
chr16-70164992-70165650
chr14-55332613-55333050
chr16-11560411-11561572
chr10-100510752-100511505
chr9-129956681-129957201
chr8-122918716-122919607
chr2-27391121-27392299
chr3-11291178-11291803
chr5-172185175-172185366
chr2-126659143-126659512
chr20-5078011-5078962
chr18-61892731-61894897
chr8-125637178-125637987
chr1-151056270-151056594
chr14-61342346-61343112
chr13-99403413-99404027
chr16-89100045-89100284
chr2-96324793-96326336
chr16-16033106-16033913
chr1-11934151-11935087
chr1-19643749-19645214

chr1-198167060-198167969
chr1-58812940-58813370
chr20-49661627-49662312
chr1-226722468-226723427
chr6-142915419-142915620
chr11-120210515-120211460
chr1-92365534-92366527
chr16-15907963-15908523
chr7-150481265-150482204
chr15-101979709-101981190
chr4-148216581-148217581
chr11-60971288-60972141
chr17-47720901-47721581
chr1-159826202-159827906
chr6-490239-490861
chr20-10673762-10675019
chr10-114540875-114541919
chr1-203625583-203627150
chr7-150450010-150452045
chr8-19475033-19475998
chr3-177201739-177202181
chr2-201126820-201127117
chr2-241803288-241805231
chr7-70595667-70596263
chr19-4104573-4105412
chr8-102782803-102783564
chr8-19209232-19209856
chr8-42355336-42356359
chr15-69668141-69669528
chr1-31939765-31940308
chr7-152144440-152145386
chr10-110414261-110415262
chr17-30291042-30292495
chr2-38875327-38876371
chr18-12419760-12420611
chr12-11981065-11981847
chr2-169732870-169734581
chr17-4696231-4697127
chr14-91405138-91405845
chr2-110675128-110675576
chr12-10293279-10294105
chr2-110846408-110847655
chr13-113584216-113585005

chr9-126396770-126397646
chr2-33275643-33276198
chr1-212950208-212951308
chrX-1467122-1467552
chr8-29060562-29061854
chr10-88831714-88832003
chr7-75727718-75728527
chr6-5518901-5519503
chr2-233079453-233079825
chr6-96836721-96838012
chr7-3112598-3113173
chr17-58328063-58330047
chr7-24910798-24911571
chr4-89290823-89291064
chr3-16309628-16310054
chr1-167446133-167446452
chr10-96670565-96671789
chr1-92484468-92486445
chr3-49813174-49814302
chr6-26240052-26241306
chr1-116806116-116806853
chr18-77124442-77125032
chr5-157603233-157603915
chr6-151690169-151691687
chr12-75934244-75935068
chr6-33578449-33579054
chr16-27401751-27402707
chr1-41806892-41807110
chr10-11149306-11151154
chr7-35729093-35729867
chr14-99262149-99263032
chr1-158930295-158931811
chr12-121038622-121039529
chr17-28726004-28727442
chr10-62637000-62638381
chr14-61379577-61380123
chr8-125272971-125274164
chr16-2065291-2065685
chr7-75237466-75238278
chr8-103445376-103446008
chr6-158564941-158565223
chr2-106095607-106097035
chr5-39187984-39188668

chr10-72369957-72370862
chr5-171418585-171420425
chr8-127793934-127795972
chrX-154712586-154713341
chr5-177412580-177413080
chr5-73095787-73096536
chr11-36558363-36559345
chr1-25031939-25032742
chr1-160664543-160666292
chr2-69650397-69650696
chr14-49999224-49999701
chr4-122458415-122459113
chr3-5026191-5027254
chr8-127980340-127981889
chr6-117547572-117549948
chr11-60994334-60995199
chr16-10876439-10877613
chr6-155174667-155175110
chr1-204521075-204521690
chr1-220056265-220056903
chr1-168512990-168514782
chr1-198649358-198650468
chr4-26312228-26312837
chr18-80172050-80172431
chr6-34673137-34674227
chr8-100493163-100493687
chr11-35172954-35174367
chr2-237475027-237476058
chr19-16328199-16329442
chr5-1518471-1519233
chr20-10672570-10673004
chr1-100028187-100029357
chr7-116962090-116962843
chr5-39211632-39212193
chr7-2405863-2406324
chr13-41811600-41812351
chr5-40361475-40361750
chr2-233990256-233991488
chr2-136423333-136424509
chr6-111846508-111847205
chr20-51022815-51023532
chr2-174675501-174676108
chr8-74320497-74321973

chr10-69222027-69223177
chr4-7863943-7865850
chr16-30473475-30473997
chr6-87742019-87742731
chr15-51981676-51982644
chr16-79599688-79601632
chr12-107369519-107370071
chr15-84736302-84736834
chr2-196182219-196184326
chr9-133370638-133371757
chr11-107858064-107859991
chr20-43949868-43950738
chr19-18879411-18880267
chr19-29587361-29588120
chr8-102122681-102124565
chr17-49387642-49388666
chr7-141590523-141591159
chr2-8301877-8302529
chr2-111493489-111495432
chr16-84518802-84519282
chr17-78381608-78382190
chr8-79692891-79693762
chr10-3093154-3093772
chr1-172778684-172779450
chr13-40613060-40614028
chr16-89331483-89331967
chr17-68182601-68183517
chr8-143950940-143951205
chr7-30330317-30331205
chr1-89721386-89721719
chr9-126804667-126806197
chr10-61942656-61944064
chr10-24887309-24888277
chr8-21918282-21918890
chr2-237432528-237433252
chr2-29314723-29315532
chr8-19473352-19473729
chr4-47010510-47011628
chr1-100396578-100397433
chr12-53205718-53206451
chr19-35462366-35463298
chr3-177426911-177427818
chr9-74496604-74498799

chr2-233076762-233076989
chr8-69832489-69835302
chr8-27361967-27362522
chr13-25017985-25018664
chr9-121074253-121075741
chr5-50489336-50490003
chr15-85329466-85330552
chr15-90574370-90574855
chr19-6669351-6670486
chr6-53302704-53303965
chr1-113783322-113784633
chr12-64657596-64658898
chr16-66522162-66522487
chr13-30374358-30374853
chr8-133066128-133066451
chr11-88301170-88302105
chr1-199173009-199173897
chr21-43864939-43867147
chr2-69946378-69947233
chr17-80764661-80765882
chr6-27830686-27831860
chr2-197304575-197305034
chr10-17428232-17429431
chr6-26196756-26197818
chr3-122077047-122078135
chr11-61293898-61295623
chr17-35537457-35537923
chr3-10460052-10460653
chr10-84330241-84330596
chr4-83231434-83232263
chr17-42287089-42289678
chr16-87777668-87778133
chr14-101819410-101820030
chr8-119855303-119856542
chr5-149729952-149732324
chr1-53600349-53601018
chr7-35806893-35807493
chr15-75836733-75837526
chr12-68450794-68452118
chr1-145690376-145691346
chr7-29194380-29195700
chr10-14184843-14186059
chr6-139530689-139531453

chr1-205440518-205441626
chr11-115214563-115215289
chr1-203628870-203630101
chr2-9752492-9753773
chr17-75875975-75876875
chr3-111543912-111544257
chr7-151408593-151409516
chr3-16311700-16312586
chr15-91857460-91858195
chr16-27446634-27447213
chr17-77462100-77462440
chr1-183956320-183957162
chr14-55098037-55098683
chr1-203606913-203607727
chr7-2120020-2120776
chr13-40115928-40116523
chr9-277516-279075
chr4-121189649-121190474
chr3-136919663-136920181
chr6-137715429-137716310
chr2-241771926-241772479
chr3-73028889-73029672
chr17-5468021-5469446
chr6-42565336-42565706
chr11-12235785-12236933
chr1-54474658-54476461
chr12-122244050-122245516
chr19-10412332-10412630
chr16-10878609-10879201
chr12-12379651-12380582
chr6-85669564-85670296
chr1-3787347-3788069
chr17-65173271-65174090
chr12-47390859-47392161
chr20-52031445-52032408
chr1-20152087-20152754
chr22-39013762-39014753
chr6-158535978-158537631
chr20-3157202-3157846
chr7-92717749-92718848
chr22-37238351-37238976
chr11-115504001-115505069
chr2-26068523-26069044

chr9-113520271-113520629
chr7-2698740-2699566
chr12-42325716-42327199
chr8-98944108-98945637
chr9-41234432-41235016
chr2-9738522-9739860
chr8-81096973-81097874
chr11-68038811-68039471
chr7-2893548-2895088
chr11-119262776-119263976
chr20-24960428-24961587
chr9-36572386-36573761
chr7-35723045-35723338
chr20-51497510-51498367
chr1-58941442-58943163
chr17-50508034-50508881
chr1-223726429-223727275
chr12-6446376-6447040
chr6-137816159-137816523
chr7-33040613-33041398
chr1-172818387-172819706
chr2-8457067-8458107
chr5-143693697-143694728
chr7-126701083-126701524
chr2-44941237-44942677
chr13-45367223-45367984
chr10-50671847-50672764
chr1-89278647-89279691
chr16-87895521-87896493
chr10-26447278-26447793
chr18-56709475-56709851
chr4-4541081-4542573
chr12-47207114-47208436
chr15-52545916-52546855
chr17-36107529-36107988
chr5-139635827-139638732
chr16-3625930-3626817
chr16-78770327-78771036
chr14-54881248-54882410
chr1-24416782-24417193
chr6-130138194-130139123
chr16-57764366-57765193
chr12-104687638-104688450

chr17-76383068-76385314
chrX-147917731-147918827
chr22-46613730-46615041
chr12-122161640-122162186
chr6-35302594-35303525
chr8-38030061-38031661
chr21-38244617-38245440
chr6-106103583-106104261
chr1-211326417-211327968
chr2-102417748-102418955
chr7-100221049-100221915
chr10-100266917-100268122
chr17-47732840-47734090
chr20-51500894-51501438
chr1-111221502-111222441
chr5-912249-913001
chr1-198936639-198937746
chr13-25901630-25902151
chr22-39316402-39316818
chr1-12178968-12179523
chr14-65791342-65792171
chr14-61350462-61351201
chr6-157535629-157536480
chr2-229714172-229715592
chr14-55337477-55338368
chr9-132350885-132351270
chr11-128628185-128629052
chr3-11641766-11644108
chr17-48429116-48430639
chr20-43666482-43667467
chr7-131235535-131236133
chr13-33786694-33787721
chr15-60584443-60585094
chr1-116377577-116378403
chr14-99235015-99235996
chr2-144508136-144508781
chr2-9843044-9844202
chr5-157180356-157181248
chr13-50911850-50912322
chr12-122227470-122227772
chr15-31367612-31367989
chr3-187918800-187920014
chr3-121431871-121432497

chr2-84782035-84783532
chr12-108635260-108636043
chr19-45087543-45087897
chr22-44023761-44024856
chr7-4744458-4746497
chr8-100443001-100444223
chr11-63531278-63532113
chr12-14410979-14411510
chr3-15054998-15055541
chr11-122981537-122982210
chr14-61403334-61403994
chr22-20556402-20558233
chr2-8477354-8478577
chr9-130007378-130007989
chr2-12727840-12728957
chr11-128464965-128465923
chr20-51416446-51417364
chr5-143229430-143230583
chr2-61016795-61018432
chr19-3132993-3133904
chr21-33238044-33240068
chr14-99246596-99247618
chr6-150068380-150069591
chr1-228646615-228648301
chr22-22752632-22753994
chr5-76542805-76543690
chr17-49208959-49211038
chr19-6521839-6522792
chr7-104956922-104957752
chr2-234483202-234484440
chr9-5841439-5842424
chr1-211675184-211676146
chr3-114221397-114222118
chrX-71619144-71619544
chr9-126516934-126517793
chr18-3605771-3606482
chr3-111616337-111617018
chr10-26486980-26487842
chr2-130824152-130825055
chr6-25137487-25138996
chr2-168489998-168491297
chr3-172523017-172524028
chr15-31345326-31345799

chr9-33164466-33164683
chr10-110859778-110861231
chr9-128030991-128031392
chr15-22220933-22221823
chr3-142269843-142271005
chr16-11258770-11259000
chr3-48663545-48664401
chr13-78602347-78603976
chr11-85823627-85824495
chr18-51191174-51191685
chr16-23504186-23505383
chrX-153985380-153985957
chr19-610063-611838
chr1-172646843-172647550
chr6-42774873-42775573
chr1-55127714-55128933
chr13-114130849-114131706
chr15-85646357-85647263
chr1-169667085-169668127
chr9-5885910-5886702
chr5-151138871-151139841
chr17-77331349-77333169
chr17-64968771-64969391
chr16-30407001-30407904
chr10-73729659-73730868
chr17-80471846-80472272
chr10-17509044-17509507
chr5-87340386-87341141
chr14-61444126-61444701
chr1-38046391-38047612
chr6-118796429-118797111
chr6-166418899-166419748
chr2-106094076-106094879
chr17-77441168-77442093
chr6-73309433-73310700
chr1-151945498-151946678
chr19-543484-544442
chr4-1203654-1203925
chr2-216015856-216016400
chr14-61471698-61472504
chr14-58151479-58152744
chr17-68346315-68347058
chr1-172670326-172671182

chr16-57577746-57578485
chr22-46612848-46613543
chr9-123637716-123638680
chr2-224927604-224928418
chr1-53738741-53739572
chr12-120959657-120960438
chr19-2620630-2621344
chr22-30425808-30426916
chr3-71066457-71066671
chr10-6578168-6578842
chr5-107570935-107572484
chr7-77351320-77352173
chr16-85612796-85613521
chr9-137034647-137035638
chr4-10095706-10096856
chr5-132103383-132104314
chr6-105994069-105995267
chr11-63866146-63866799
chr20-35283766-35285233
chrX-24693424-24694322
chr8-29049977-29050582
chr4-88007205-88008781
chr18-77073604-77074279
chr20-53608655-53610301
chrX-154372686-154372916
chr13-97226848-97227489
chr6-33579570-33580641
chr7-5531835-5532228
chr8-9011734-9012460
chr9-120928442-120929517
chr4-48129435-48130023
chr6-138161074-138162620
chr11-64206385-64208298
chr5-88140925-88142053
chr13-98476217-98477015
chr11-128492681-128493685
chr4-26884055-26885685
chr12-6631482-6632724
chr11-35077650-35078594
chr22-36376931-36378005
chr1-169694375-169694890
chr20-48826825-48828609
chr1-160797870-160798280

chr14-105857649-105858587
chr4-77852902-77853557
chr1-40059328-40060688
chr12-120007044-120007811
chr12-64458883-64460049
chr12-111429947-111431245
chr8-116437178-116438557
chr1-116389363-116390620
chr17-80254684-80255227
chr12-6866975-6868335
chr4-1267283-1268225
chr5-142015085-142016450
chr2-96343893-96344531
chr2-39128951-39129383
chr10-33008644-33009253
chr22-23216121-23217042
chr1-160432349-160432971
chr12-92539077-92541223
chr17-35930445-35931133
chr17-50943773-50945140
chr2-196177734-196178377
chr1-204564759-204566283
chr17-64708601-64709452
chr14-35533204-35535765
chr3-14424508-14425239
chrX-71533796-71534571
chr6-111844456-111845227
chr1-167627677-167628750
chr1-67194122-67195288
chr15-40753973-40754378
chr1-160767079-160768789
chr6-16483250-16483940
chr1-230123037-230124195
chr20-59163494-59164215
chr2-73293264-73294001
chr1-16420554-16421453
chr2-54559826-54560844
chr6-125918616-125919590
chr6-25034420-25036517
chr18-32040423-32041546
chr18-80175145-80176077
chr7-50610391-50611145
chr1-212595840-212596923

chr13-48403159-48404198
chr19-13167372-13168254
chr16-11584084-11587626
chr7-130938339-130938995
chr11-61567110-61567789
chr19-50325624-50325853
chr18-48948516-48950153
chr1-89670062-89670513
chr14-106174272-106174814
chr14-80941208-80942571
chr1-92482944-92484024
chr6-137717358-137717890
chr10-22633932-22634508
chr8-100258389-100259420
chr12-53208854-53209408
chr1-160707395-160707848
chr22-36364819-36365430
chr3-71226221-71227605
chr9-89448413-89448849
chr18-13376108-13377773
chr14-106510863-106511634
chr17-2222861-2223684
chr9-19183921-19184872
chr20-53751019-53751899
chr2-195532349-195533261
chr12-25241006-25241922
chr1-6259772-6261369
chr4-89316316-89317490
chr17-68356210-68357251
chr17-75242175-75243013
chr11-65575464-65576116
chr19-41887046-41888287
chr16-30470965-30471393
chr2-233083932-233085005
chr2-96485746-96486649
chr3-45664324-45665662
chr12-113135422-113136505
chr12-122901355-122901991
chr2-25314276-25315033
chr20-35251825-35252798
chr10-6052479-6053080
chr2-37623976-37624798
chrX-119955810-119956901

chr18-80246769-80247930
chr9-121132863-121133716
chr12-110562038-110563673
chr14-91867008-91867876
chr11-126355122-126356890
chr9-89463792-89464956
chr8-127975549-127976205
chr19-16594093-16595413
chr10-79317367-79318319
chr14-23988267-23989356
chr9-136996325-136996870
chr15-76994706-76995705
chr22-36379420-36379964
chr2-131153735-131154569
chr1-184973396-184974763
chr13-79194185-79194968
chr8-33471801-33473607
chr8-129885435-129887130
chr9-89443991-89444664
chr1-89854303-89856339
chr11-414449-415532
chr1-23612948-23614382
chr1-42936707-42937506
chr2-8544866-8545508
chr19-8268566-8269689
chr2-105795749-105796558
chr13-27361872-27362614
chr11-65431407-65431733
chr6-11224273-11225482
chr14-91869825-91870419
chr17-19063237-19064542
chr20-17530551-17531720
chr11-313857-314966
chr8-27325174-27327254
chr5-139392281-139393447
chr4-83125647-83126219
chr8-66534998-66536133
chr15-50247580-50248604
chr9-130039556-130040011
chr19-38735958-38736421
chr8-125644260-125644968
chr1-160738943-160739520
chr19-1408496-1409043

chr2-144517127-144517992
chr10-3095377-3096490
chr15-78067880-78069338
chr12-89880488-89881320
chr6-73262542-73263698
chr16-22357405-22358462
chr6-35919633-35921414
chr2-10122402-10123002
chr14-92585600-92587586
chr17-48439664-48440500
chr2-97745508-97746058
chr22-49961958-49962799
chr1-44813585-44814106
chr16-30458591-30459343
chr8-143558044-143559062
chr20-32249078-32249833
chr3-14425768-14426473
chr16-8891271-8892174
chr7-2521870-2522593
chr6-166244088-166245174
chr7-50340018-50340538
chr3-154120942-154122706
chr7-50352128-50352665
chr16-22436518-22438042
chr17-78186473-78187565
chr2-102355500-102356566
chr10-91796339-91797075
chr2-96321034-96322363
chr13-40984723-40985264
chr5-131271963-131273064
chr16-88633633-88635135
chr19-11639434-11640319
chr19-18119145-18120076
chr15-84734788-84736017
chr3-191328577-191330270
chr18-9116823-9117660
chr4-15778020-15779790
chr14-105473712-105474904
chr14-91388311-91389034
chr15-40047450-40048722
chr19-3178360-3179809
chr15-75130724-75131650
chr19-2327583-2329020

chr7-139104191-139105249
chr20-41047711-41049315
chr12-121658446-121659576
chr6-33633359-33634253
chr17-77453521-77454203
chr11-63813014-63814222
chr7-6105692-6106721
chr17-39867140-39868202
chr19-10408583-10409290
chr6-108895590-108896707
chr1-234925943-234927397
chr12-27332584-27333855
chr22-36386504-36387320

Cluster	Genes
CD4 CTL	PDGFD
CD4 CTL	CX3CR1
CD4 CTL	ADGRG1
CD4 CTL	FCRL6
CD4 CTL	C1orf21
CD4 CTL	PRSS23
CD4 CTL	TMCC3
CD4 CTL	S1PR5
CD4 CTL	ASCL2
CD4 CTL	CCL4
CD4 CTL	XCL2
CD4 CTL	FGFBP2
CD4 CTL	SLAMF7
CD4 CTL	EOMES
CD4 CTL	GZMH
CD4 CTL	CES1
CD4 CTL	TGFBR3
CD4 CTL	NKG7
CD4 CTL	GZMM
CD4 CTL	CCL5
CD4 CTL	B2M
CD4 CTL	CST7
CD4 CTL	ENC1
CD4 CTL	HCST
CD4 CTL	FGR
CD4 CTL	HBA1
CD4 CTL	MAP3K8
CD4 CTL	EFHD2
CD4 CTL	SPON2
CD4 CTL	PROK2
CD4 Naive	NOG
CD4 Naive	LRRN3
CD4 Naive	SCML1
CD4 Naive	CCR7
CD4 Naive	AIF1
CD4 Naive	TRABD2A
CD4 Naive	BCL11B
CD4 Naive	ABLIM1
CD4 Naive	TMIGD2
CD4 Naive	AK5
CD4 Naive	IL6ST
CD4 Naive	RPL11

CD4 Naive	OXNAD1
CD4 Naive	SOX4
CD4 Naive	SATB1
CD4 Naive	BACH2
CD4 Naive	LEF1
CD4 Naive	RETREG1
CD4 Proliferating	DTL
CD4 Proliferating	E2F8
CD4 Proliferating	CDC45
CD4 Proliferating	CDT1
CD4 Proliferating	DLGAP5
CD4 Proliferating	TK1
CD4 Proliferating	HJURP
CD4 Proliferating	CDCA2
CD4 Proliferating	MCM10
CD4 Proliferating	TROAP
CD4 Proliferating	RRM2
CD4 Proliferating	ANLN
CD4 Proliferating	KIF4A
CD4 Proliferating	MYBL2
CD4 Proliferating	PKMYT1
CD4 Proliferating	CDCA5
CD4 Proliferating	TYMS
CD4 Proliferating	E2F7
CD4 Proliferating	CCNB2
CD4 Proliferating	NUSAP1
CD4 Proliferating	MKI67
CD4 Proliferating	BIRC5
CD4 Proliferating	DSCC1
CD4 Proliferating	PCLAF
CD4 Proliferating	GTSE1
CD4 Proliferating	CDK1
CD4 Proliferating	UHRF1
CD4 Proliferating	KIF15
CD4 Proliferating	PCNA
CD4 Proliferating	STMN1
CD4 TCM_0	TSHZ2
CD4 TCM_0	RPS27
CD4 TCM_0	CCR7
CD4 TCM_0	PASK
CD4 TCM_0	TCF7
CD4 TCM_0	RPL32
CD4 TCM_0	IL6ST

CD4 TCM_0	ADTRP
CD4 TCM_0	FYB1
CD4 TCM_0	SELL
CD4 TCM_0	SESN3
CD4 TCM_0	MAL
CD4 TCM_0	SEC11C
CD4 TCM_0	MYH9
CD4 TCM_0	DHRS7
CD4 TCM_0	CCND2
CD4 TCM_0	PFDN2
CD4 TCM_0	SSBP4
CD4 TCM_0	PBXIP1
CD4 TCM_0	JPT1
CD4 TCM_0	ATP2B1
CD4 TCM_0	TMEM50A
CD4 TCM_0	HLA-B
CD4 TCM_0	ELOVL5
CD4 TCM_1	NEFL
CD4 TCM_1	LMNA
CD4 TCM_1	FXYD7
CD4 TCM_1	RCBTB2
CD4 TCM_1	GATA3
CD4 TCM_1	FXYD1
CD4 TCM_1	LPAR6
CD4 TCM_1	LMO4
CD4 TCM_1	VIM
CD4 TCM_1	C1orf162
CD4 TCM_1	CAPG
CD4 TCM_1	NR3C1
CD4 TCM_1	FXYD5
CD4 TCM_1	PLP2
CD4 TCM_1	ANKRD28
CD4 TCM_1	CD82
CD4 TCM_1	CRIP2
CD4 TCM_1	PTGER4
CD4 TCM_1	GLIPR1
CD4 TCM_1	INPP4B
CD4 TCM_1	ANXA1
CD4 TCM_1	ARHGAP15
CD4 TCM_1	KRT1
CD4 TCM_1	MDFIC
CD4 TCM_1	TIMP1
CD4 TCM_1	TOB1

CD4 TCM_1	AHNAK
CD4 TCM_1	ZFP36L2
CD4 TCM_1	PAG1
CD4 TCM_2	FYB1
CD4 TCM_2	GYPC
CD4 TCM_2	CORO1B
CD4 TCM_2	CNN2
CD4 TCM_2	OPTN
CD4 TCM_2	LAPTM5
CD4 TCM_2	ACTG1
CD4 TCM_2	GMFG
CD4 TCM_2	ITGB1
CD4 TCM_2	UCP2
CD4 TCM_2	C16orf87
CD4 TCM_2	TMSB10
CD4 TCM_2	LAT
CD4 TCM_2	EML4
CD4 TCM_2	CD28
CD4 TCM_2	LGALS9
CD4 TCM_2	EPSTI1
CD4 TCM_2	MAF
CD4 TCM_2	SAT1
CD4 TCM_2	PPP1CC
CD4 TCM_2	MXD4
CD4 TCM_2	EVI2B
CD4 TCM_2	COTL1
CD4 TCM_2	JAK3
CD4 TCM_2	CAPZB
CD4 TCM_2	SLC4A7
CD4 TCM_2	PBXIP1
CD4 TCM_2	OCIAD2
CD4 TCM_2	EIF3A
CD4 TCM_3	KLRB1
CD4 TCM_3	PDE4D
CD4 TCM_3	RPS19
CD4 TCM_3	USP10
CD4 TCM_3	LTB
CD4 TCM_3	DPP4
CD4 TCM_3	CMTM6
CD4 TCM_3	AQP3
CD4 TCM_3	TNFRSF4
CD4 TEM_0	ITGA4
CD4 TEM_0	KLRB1

CD4 TEM_0	TMEM156
CD4 TEM_0	DYNLT3
CD4 TEM_0	SERINC5
CD4 TEM_0	RPS6
CD4 TEM_0	TCF7
CD4 TEM_0	MGAT4A
CD4 TEM_0	LIMS1
CD4 TEM_0	INPP4B
CD4 TEM_0	EEF1B2
CD4 TEM_0	IL7R
CD4 TEM_0	GPR183
CD4 TEM_0	LTB
CD4 TEM_0	ANKRD12
CD4 TEM_0	ITGA4
CD4 TEM_0	TCF7
CD4 TEM_0	STK17A
CD4 TEM_0	SERINC5
CD4 TEM_0	GCC2
CD4 TEM_0	OXNAD1
CD4 TEM_0	CCL5
CD4 TEM_0	AP3M2
CD4 TEM_0	ATM
CD4 TEM_0	FYN
CD4 TEM_0	FYB1
CD4 TEM_0	LEF1
CD4 TEM_0	FOXP1
CD4 TEM_0	ATXN7L3B
CD4 TEM_0	NUCB2
CD4 TEM_0	CCR7
CD4 TEM_0	PIK3R1
CD4 TEM_0	ETS1
CD4 TEM_0	STK17B
CD4 TEM_0	TRA2A
CD4 TEM_0	DNMT3A
CD4 TEM_0	MCUB
CD4 TEM_0	H1FX
CD4 TEM_0	PPP2R5C
CD4 TEM_0	DHX36
CD4 TEM_0	N4BP2L2
CD4 TEM_0	PTP4A2
CD4 TEM_0	ARID5B
CD4 TEM_0	YPEL5
CD4 TEM_0	CLEC2D

CD4 TEM_0	EMB
CD4 TEM_0	PIK3IP1
CD4 TEM_0	CBLB
CD4 TEM_1	GZMK
CD4 TEM_1	LYAR
CD4 TEM_1	DUSP2
CD4 TEM_1	GZMA
CD4 TEM_1	ZFP36L2
CD4 TEM_1	MT-ND3
CD4 TEM_1	ID2
CD4 TEM_1	TNFAIP3
CD4 TEM_1	XCL1
CD4 TEM_1	BTG1
CD4 TEM_1	HCST
CD4 TEM_1	PIK3R1
CD4 TEM_1	CCL5
CD4 TEM_1	GZMM
CD4 TEM_1	HOPX
CD4 TEM_1	CXCR4
CD4 TEM_1	CEBPB
CD4 TEM_1	KLRB1
CD4 TEM_1	PDCD4
CD4 TEM_1	SYNE2
CD4 TEM_1	HNRNPLL
CD4 TEM_1	IL7R
CD4 TEM_1	PARP8
CD4 TEM_2	NPTX1
CD4 TEM_2	COL24A1
CD4 TEM_2	ZNF462
CD4 TEM_2	CD79A
CD4 TEM_2	LTB
CD4 TEM_2	JUNB
CD4 TEM_2	PLXNA4
CD4 TEM_2	CD55
CD4 TEM_2	ARL4C
CD4 TEM_2	SOCS3
CD4 TEM_2	TPT1
CD4 TEM_2	CR1
CD4 TEM_2	JUND
CD4 TEM_2	ARHGAP15
CD4 TEM_2	RPL19
CD4 TEM_2	UBA52
CD4 TEM_2	PRKD3

CD4 TEM_2	AC004086.1
CD4 TEM_2	IRF2BP2
CD4 TEM_2	BTG2
CD4 TEM_2	RPL8
CD4 TEM_2	TIMP1
CD4 TEM_2	NFKBIA
CD4 TEM_2	HNRNPA1
CD4 TEM_2	BCL2
CD4 TEM_2	RPS14
CD4 TEM_2	NACA
Treg	FANK1
Treg	FOXP3
Treg	HACD1
Treg	FCRL3
Treg	CCDC141
Treg	C15orf53
Treg	IL2RA
Treg	IKZF2
Treg	CPNE2
Treg	DUSP4
Treg	RTKN2
Treg	TTN
Treg	STAM
Treg	LGALS3
Treg	TIGIT
Treg	HPGD
Treg	TBC1D4
Treg	SETD7
Treg	CTLA4
Treg	CCNG2
Treg	UBL3
Treg	RGS1
Treg	HLA-DQB1
Treg	TTC39C
Treg	PMAIP1
Treg	TCEAL4
Treg	USP15
Treg	SAMHD1
Treg	CASK
Treg	TULP4
CD8 Naive	LRRN3
CD8 Naive	AK5
CD8 Naive	SCML1

CD8 Naive	TMIGD2
CD8 Naive	CLEC11A
CD8 Naive	EIF3E
CD8 Naive	CCR7
CD8 Naive	RPL23
CD8 Naive	ACTN1
CD8 Naive	TRABD2A
CD8 Naive	MAL
CD8 Naive	S100B
CD8 Naive	ARMH1
CD8 Naive	FHIT
CD8 Naive	IL6ST
CD8 Naive	SATB1
CD8 Naive	AIF1
CD8 Naive	NDFIP1
CD8 Naive	KLF7
CD8 Naive	OXNAD1
CD8 Naive	ABLIM1
CD8 Naive	PDK1
CD8 Naive	LEF1
CD8 Naive	MYC
CD8 Naive	TTN
CD8 Naive	RUNX2
CD8 Naive	RETREG1
CD8 Naive	SNHG32
CD8 Naive	RCAN3
CD8 Naive	ITGA6
CD8 TCM	NT5E
CD8 TCM	SPINK2
CD8 TCM	IL7R
CD8 TCM	BIRC3
CD8 TCM	ITGA1
CD8 TCM	TMIGD2
CD8 TCM	TNFRSF25
CD8 TCM	CDC14A
CD8 TCM	SPRY1
CD8 TCM	TTC39C
CD8 TCM	TMEM123
CD8 TCM	ITM2C
CD8 TCM	PAG1
CD8 TCM	JUN
CD8 TCM	ARHGAP15
CD8 TCM	RCAN3

CD8 TCM	ODF2L
CD8 TCM	MDFIC
CD8 TCM	FOSB
CD8 TCM	KLRC1
CD8 TCM	SERINC5
CD8 TCM	LTB
CD8 TCM	KLF6
CD8 TCM	KLRB1
CD8 TCM	SH3YL1
CD8 TCM	RORA
CD8 TCM	MAL
CD8 TCM	TCF7
CD8 TCM	IRS2
CD8 TCM	FOS
CD8 TEM_0	AKR1C3
CD8 TEM_0	FGFBP2
CD8 TEM_0	PROK2
CD8 TEM_0	SPON2
CD8 TEM_0	GZMH
CD8 TEM_0	B2M
CD8 TEM_0	CD8A
CD8 TEM_0	ADGRG1
CD8 TEM_0	ITGAL
CD8 TEM_0	PRSS23
CD8 TEM_0	CES1
CD8 TEM_0	LAIR2
CD8 TEM_0	MAF
CD8 TEM_0	PSTPIP2
CD8 TEM_0	SH3BGRL3
CD8 TEM_0	CD3G
CD8 TEM_0	ADRB2
CD8 TEM_0	VCL
CD8 TEM_0	RAP2A
CD8 TEM_0	S100A4
CD8 TEM_0	PLEKHF1
CD8 TEM_0	HLA-B
CD8 TEM_0	RAP1B
CD8 TEM_0	ID2
CD8 TEM_0	CST7
CD8 TEM_0	FNDC3B
CD8 TEM_0	ZFYVE28
CD8 TEM_0	PTPRC
CD8 TEM_0	PTCH1

CD8 TEM_0	HBA1
CD8 TEM_1	CD160
CD8 TEM_1	CMC1
CD8 TEM_1	GZMM
CD8 TEM_1	KLRF1
CD8 TEM_1	KLRB1
CD8 TEM_1	AOAH
CD8 TEM_1	APMAP
CD8 TEM_1	PTP4A2
CD8 TEM_1	STAT4
CD8 TEM_1	CCL3
CD8 TEM_1	CD81
CD8 TEM_1	TSEN54
CD8 TEM_1	EOMES
CD8 TEM_1	XCL2
CD8 TEM_1	PYHIN1
CD8 TEM_1	CCL4
CD8 TEM_1	HBA1
CD8 TEM_1	DTHD1
CD8 TEM_1	FYN
CD8 TEM_1	C1orf21
CD8 TEM_1	S1PR5
CD8 TEM_1	NCOA1
CD8 TEM_1	KDELR2
CD8 TEM_1	JAZF1
CD8 TEM_1	ZEB2
CD8 TEM_1	PARP15
CD8 TEM_1	PTPN12
CD8 TEM_1	GNG2
CD8 TEM_1	ZBTB38
CD8 TEM_1	CDKN2D
CD8 TEM_2	NCAM1
CD8 TEM_2	GNLY
CD8 TEM_2	TYROBP
CD8 TEM_2	HOPX
CD8 TEM_2	CD63
CD8 TEM_2	COL6A2
CD8 TEM_2	AC005747.1
CD8 TEM_2	KLRC2
CD8 TEM_2	MXRA7
CD8 TEM_2	AHNAK
CD8 TEM_2	RRBP1
CD8 TEM_2	TBX21

CD8 TEM_2	IFITM2
CD8 TEM_2	GSAP
CD8 TEM_2	MT-ND5
CD8 TEM_2	AGPAT4
CD8 TEM_2	ITGB1
CD8 TEM_2	ITGAM
CD8 TEM_2	TNFRSF18
CD8 TEM_2	CTSW
CD8 TEM_2	FAM129A
CD8 TEM_2	DSTN
CD8 TEM_2	SYTL3
CD8 TEM_2	KIR2DL1
CD8 TEM_2	MATK
CD8 TEM_2	ZBTB7A
CD8 TEM_2	KLRF1
CD8 TEM_2	S100A10
CD8 TEM_2	KIR3DL2
CD8 TEM_2	CD247
CD8 TEM_3	GZMK
CD8 TEM_3	DUSP2
CD8 TEM_3	RGS1
CD8 TEM_3	CMC1
CD8 TEM_3	CXCR4
CD8 TEM_3	YBX3
CD8 TEM_3	PHACTR2
CD8 TEM_3	SELENOM
CD8 TEM_3	BTG1
CD8 TEM_3	CRTAM
CD8 TEM_3	DNAJB1
CD8 TEM_3	FCRL3
CD8 TEM_3	TENT5C
CD8 TEM_3	ZFP36L2
CD8 TEM_3	PDCD4
CD8 TEM_3	RPS27
CD8 TEM_3	PIK3R1
CD8 TEM_3	MAP3K8
CD8 TEM_3	CD160
CD8 TEM_3	PDE4B
CD8 TEM_3	IFNGR1
CD8 TEM_3	CD69
CD8 TEM_3	FYN
CD8 TEM_3	CD74
CD8 TEM_3	TSPYL2

CD8 TEM_3	JUNB
CD8 TEM_3	LYST
CD8 TEM_3	GPR183
CD8 TEM_3	CD84
CD8 TEM_3	SH2D1A
CD8 TEM_4	SOX4
CD8 TEM_4	SPINT2
CD8 TEM_4	IGHD
CD8 TEM_4	RTKN2
CD8 TEM_4	IFITM3
CD8 TEM_4	IRF2BP2
CD8 TEM_4	ZFP36L2
CD8 TEM_4	NCR3
CD8 TEM_4	CD7
CD8 TEM_4	IER2
CD8 TEM_4	PABPC1
CD8 TEM_4	BEX4
CD8 TEM_4	RALGPS2
CD8 TEM_4	FXYD2
CD8 TEM_4	AREG
CD8 TEM_4	MAP3K1
CD8 TEM_4	JUNB
CD8 TEM_4	NCF1
CD8 TEM_4	BTG2
CD8 TEM_4	LYAR
CD8 TEM_4	NUCB2
CD8 TEM_4	ZNF683
CD8 TEM_4	DUSP2
CD8 TEM_4	LEF1
CD8 TEM_4	BEX2
CD8 TEM_4	LTB
CD8 TEM_4	DUSP1
CD8 TEM_4	SOCS3
CD8 TEM_4	XCL1
CD8 TEM_4	GADD45A
CD8 TEM_5	KIR3DL1
CD8 TEM_5	GNLY
CD8 TEM_5	EFHD2
CD8 TEM_5	KIR2DL1
CD8 TEM_5	NKG7
CD8 TEM_5	B2M
CD8 TEM_5	GZMB
CD8 TEM_5	PRF1

CD8 TEM_5	IFITM2
CD8 TEM_5	GZMH
CD8 TEM_5	TBX21
CD8 TEM_5	ZEB2
CD8 TEM_5	CYBA
CD8 TEM_5	CD63
CD8 TEM_5	SYNE1
CD8 TEM_5	KLRD1
CD8 TEM_5	S1PR5
CD8 TEM_5	CTSW
CD8 TEM_5	FCGR3A
CD8 TEM_5	FGFBP2
CD8 TEM_5	IKZF2
CD8 TEM_5	KLRC2
CD8 TEM_5	CCL5
CD8 TEM_5	MYO1F
CD8 TEM_5	CAST
CD8 TEM_5	KANSL1
CD8 TEM_5	PRSS23
CD8 TEM_5	ABHD17A
CD8 TEM_5	C1orf21
CD8 TEM_5	ASCL2
CD8 TEM_5	RAB14
CD8 TEM_5	SUPT16H
CD8 TEM_5	UBLCP1
CD8 TEM_5	NKG7
CD8 TEM_5	ARPC2
CD8 TEM_5	CYCS
CD8 TEM_5	IL2RG
CD8 TEM_5	TRBC1
CD8 TEM_5	PPP1CA
CD8 TEM_5	APOL6
CD8 TEM_5	COX6B1
CD8 TEM_5	PET100
CD8 TEM_5	CSGALNACT2
CD8 TEM_6	NELL2
CD8 TEM_6	IL7R
CD8 TEM_6	GPR183
CD8 TEM_6	PASK
CD8 TEM_6	FOXP1
CD8 TEM_6	CCR7
CD8 TEM_6	JUNB
CD8 TEM_6	TCF7

CD8 TEM_6	RPS23
CD8 TEM_6	RCAN3
VI-A	GMFG
VI-A	CD27
VI-A	ACTG1
VI-A	LIMD2
VI-A	CD74
VI-A	ARHGDIB
VI-A	GZMA
VI-A	COTL1
VI-A	ATP5F1A
VI-A	ARPC3
VI-A	HLA-DRA
VI-A	SUB1
VI-A	CORO1A
VI-A	RTRAF
VI-A	WDR1
VI-A	ATP5MC2
VI-A	ACTB
VI-A	PSMB8
VI-A	CNN2
VI-A	ATP5MG
VI-A	HIGD2A
VI-A	PSMA5
VI-A	TMSB4X
VI-A	PYCARD
VI-A	MYL6
VI-A	ARPC1B
VI-A	HINT1
VI-A	CCL5
VI-A	ACTR3
VI-A	PSMA4
VI-B	PKMYT1
VI-B	E2F7
VI-B	SPC25
VI-B	CCNB2
VI-B	CDC45
VI-B	FAM111B
VI-B	MYBL2
VI-B	DTL
VI-B	CLSPN
VI-B	MND1
VI-B	TYMS

VI-B	CDK1
VI-B	DLGAP5
VI-B	CDCA5
VI-B	CDC6
VI-B	HIST1H3G
VI-B	TK1
VI-B	CKAP2L
VI-B	ASF1B
VI-B	CDT1
VI-B	MKI67
VI-B	CENPF
VI-B	ZWINT
VI-B	PCLAF
VI-B	KNL1
VI-B	RRM2
VI-B	CENPU
VI-B	PBK
VI-B	MCM10
VI-B	CEP55
NK Proliferating	TK1
NK Proliferating	CDK1
NK Proliferating	ESCO2
NK Proliferating	SPC25
NK Proliferating	E2F7
NK Proliferating	DTL
NK Proliferating	TYMS
NK Proliferating	E2F1
NK Proliferating	FAM111B
NK Proliferating	UHRF1
NK Proliferating	CDT1
NK Proliferating	PCLAF
NK Proliferating	HJURP
NK Proliferating	CHEK1
NK Proliferating	DIAPH3
NK Proliferating	POLQ
NK Proliferating	CLSPN
NK Proliferating	ZWINT
NK Proliferating	RRM2
NK Proliferating	KIF11
NK Proliferating	CKAP2L
NK Proliferating	RAD51AP1
NK Proliferating	MCM4
NK Proliferating	HELLS

NK Proliferating	MCM10
NK Proliferating	PCNA
NK Proliferating	CKS1B
NK Proliferating	STMN1
NK Proliferating	DUT
NK Proliferating	HMGB2
NK_0	CCL5
NK_0	PPP2R5C
NK_0	ZEB2
NK_0	F2R
NK_0	AHNAK
NK_0	LAG3
NK_0	GZMH
NK_0	PATL2
NK_0	KLF12
NK_0	CDC42SE2
NK_0	TGFBR3
NK_0	SYNE2
NK_0	DDX24
NK_0	EPS15
NK_0	PTPRC
NK_0	MYH9
NK_0	DDX6
NK_0	SUN2
NK_0	RPS27
NK_0	PTMS
NK_0	ARID5B
NK_0	IL32
NK_0	CD3G
NK_0	FLNA
NK_0	ITGB1
NK_0	HERC1
NK_0	PRDM1
NK_0	S100A6
NK_0	TGFBR1
NK_0	CCDC88C
NK_1	KLRB1
NK_1	ITGA6
NK_1	CLIC3
NK_1	CD160
NK_1	CEBPD
NK_1	HELZ
NK_1	GSAP

NK_1	EIF3G
NK_1	IFNGR1
NK_1	ARHGAP9
NK_1	JAK1
NK_1	HSH2D
NK_1	PRKCH
NK_1	KLRF1
NK_1	MYBL1
NK_1	GZMM
NK_1	TIGIT
NK_1	ZFP36
NK_1	JUNB
NK_1	CMC1
NK_1	TLE1
NK_1	SH2D2A
NK_1	SIRT2
NK_1	TENT5C
NK_1	IL2RB
NK_1	BTG2
NK_1	IRS2
NK_1	TRIM22
NK_1	PTPN12
NK_1	B3GNT7
NK_2	FGFBP2
NK_2	PTPRA
NK_2	S1PR5
NK_2	GNPTAB
NK_2	FCRL6
NK_2	KIR2DL3
NK_2	NKG7
NK_2	PTGDS
NK_2	MAF
NK_2	ITGB7
NK_2	CEBPB
NK_2	MTSS1
NK_2	RAPGEF1
NK_2	CST7
NK_2	KIR3DL1
NK_2	S100A4
NK_2	S100A6
NK_2	GZMH
NK_2	ZEB2
NK_2	PRSS23

NK_2	KIR2DL3
NK_2	DSTN
NK_2	RAPGEF1
NK_2	KIR2DL1
NK_2	RAP2A
NK_2	LGALS1
NK_2	SCP2
NK_2	IL32
NK_2	CCL5
NK_2	FCRL6
NK_3	KIR2DL1
NK_3	FGFBP2
NK_3	PRSS23
NK_3	HIPK2
NK_3	FCGR3A
NK_3	PSAP
NK_3	NKG7
NK_3	SPON2
NK_3	ADGRG1
NK_3	CST7
NK_3	KIR3DL2
NK_3	HAVCR2
NK_3	CHST2
NK_3	PRF1
NK_3	RASSF4
NK_3	RABAC1
NK_3	GZMB
NK_3	ARPC2
NK_3	TMEM71
NK_3	TYROBP
NK_3	PREX1
NK_3	MTSS1
NK_3	ITK
NK_3	FCRL6
NK_3	SH2D2A
NK_3	GZMM
NK_3	PXN
NK_3	PTGDS
NK_3	IGF2R
NK_3	PTPN12
NK_CD56bright	KIT
NK_CD56bright	PACSIN1
NK_CD56bright	IGFBP4

NK_CD56bright	SPINK2
NK_CD56bright	SPTSSB
NK_CD56bright	AFF3
NK_CD56bright	TNFRSF11A
NK_CD56bright	MAML3
NK_CD56bright	PPP1R9A
NK_CD56bright	GAB1
NK_CD56bright	RUNX2
NK_CD56bright	XCL1
NK_CD56bright	HAPLN3
NK_CD56bright	GPR183
NK_CD56bright	SELL
NK_CD56bright	TPT1
NK_CD56bright	CMC1
NK_CD56bright	CAPG
NK_CD56bright	IL7R
NK_CD56bright	IL18
NK_CD56bright	GZMK
HSPC	SMIM24
HSPC	DDAH2
HSPC	ARMH1
HSPC	BCL11A
HSPC	PRSS57
HSPC	CYTL1
HSPC	DPPA4
HSPC	SPINK2
HSPC	SOX4
HSPC	LAPTM4B
HSPC	HACD1
HSPC	CD74
HSPC	STMN1
HSPC	HLA-DRB1
HSPC	CD34
HSPC	NPR3
HSPC	GATA2
HSPC	MYB
HSPC	BAALC
HSPC	RAB34
HSPC	LMO2
HSPC	IL18
HSPC	RPS24
HSPC	MYL6B
HSPC	AIF1

HSPC	EREG
HSPC	HLA-DRA
HSPC	TXN
HSPC	ALDH1A1
HSPC	MGST2
ILC	IL32
ILC	KLRB1
ILC	TXNIP
ILC	CD48
ILC	GATA3
ILC	S100A4
ILC	NCR3
ILC	LTB
ILC	NFKBIA
ILC	BTG1
ILC	TRDC
ILC	IL7R
ILC	FXYD7
ILC	CD69
ILC	CD52
ILC	IFITM2
ILC	CD7
ILC	JUNB
ILC	S100A6
ILC	RPLP2
ILC	FAM107B
ILC	TNFRSF25
ILC	IL2RA
ILC	IER2
ILC	ETS1
ILC	XCL1
ILC	FXYD1
ILC	RORA
ILC	MAF
ILC	EMP3
Platelet	PF4
Platelet	TUBB1
Platelet	TREML1
Platelet	TMEM40
Platelet	PF4V1
Platelet	CTTN
Platelet	CLEC1B
Platelet	PPBP

Platelet	GP9
Platelet	F13A1
Platelet	CMTM5
Platelet	GNAZ
Platelet	NRGN
Platelet	PTCRA
Platelet	MYL9
Platelet	CLDN5
Platelet	SELP
Platelet	MPIG6B
Platelet	CD9
Platelet	CA2
Platelet	ITGB3
Platelet	BEND2
Platelet	ABCC3
Platelet	MFAP3L
Platelet	OSBP2
Platelet	CAVIN2
Platelet	SMOX
Platelet	GNG11
Platelet	SH3BGRL2
Platelet	ACRBP
dnT	COTL1
dnT	ACTG1
dnT	F5
dnT	SMCO4
dnT	LYST
dnT	ACTB
dnT	GPR183
dnT	SESTD1
dnT	LAYN
dnT	FXYD2
dnT	MYB
dnT	CD68
dnT	TIMD4
dnT	PGM2L1
dnT	HLA-DRA
dnT	CAPG
dnT	EVI2B
dnT	PSMB8
dnT	CD27
dnT	POU2F2
dnT	SAT1

dnT	AIF1
dnT	DENND2D
dnT	RGS10
dnT	PCBP1
dnT	DUSP6
dnT	CD74
dnT	CALM3
dnT	FYB1
dnT	CTLA4
gdT_0	TRDV2
gdT_0	KLRC1
gdT_0	TRGC1
gdT_0	DUSP2
gdT_0	XCL2
gdT_0	CD160
gdT_0	TRGV9
gdT_0	MAP3K8
gdT_0	IL18RAP
gdT_0	KLRG1
gdT_0	PIK3R1
gdT_0	TC2N
gdT_0	CD300A
gdT_0	CD69
gdT_0	TNFAIP3
gdT_0	KLRB1
gdT_0	FKBP11
gdT_0	PDE4B
gdT_0	TLE1
gdT_0	TENT5C
gdT_0	TGFBR3
gdT_0	GABARAPL1
gdT_0	TBX21
gdT_0	TRDC
gdT_0	ALOX5AP
gdT_0	CCL4
gdT_0	RORA
gdT_0	IFNGR1
gdT_0	SYTL2
gdT_0	CEBPD
gdT_1	ADGRG1
gdT_1	GZMH
gdT_1	CLIC3
gdT_1	PLAAT3

gdT_1	SERPINB6
gdT_1	ZEB2
gdT_1	NKG7
gdT_1	CYBA
gdT_1	SSBP3
gdT_1	GZMM
gdT_1	LILRB1
gdT_1	IFITM2
gdT_1	PRSS23
gdT_1	GZMB
gdT_1	PDGFD
gdT_1	FCRL6
gdT_1	EFHD2
gdT_1	PRF1
gdT_1	AHNAK
gdT_1	F2R
gdT_1	PDIA3
gdT_1	ITGB1
gdT_1	GNLY
gdT_1	CD320
gdT_1	CST7
gdT_1	DHRS7
gdT_1	ITGAL
gdT_1	FGFBP2
gdT_1	ABHD17A
gdT_1	CTSW
gdT_2	MME
gdT_2	RTKN2
gdT_2	SOX4
gdT_2	MAP3K1
gdT_2	MAL
gdT_2	LEF1
gdT_2	CDC14A
gdT_2	EPHA4
gdT_2	BEX3
gdT_2	ITGA6
gdT_2	CAMK4
gdT_2	TRABD2A
gdT_2	ITPKB
gdT_2	JUN
gdT_2	CR1
gdT_2	HSPB1
gdT_2	RCAN3

gdT_2	NUCB2
gdT_2	TCF7
gdT_2	IRF2BP2
gdT_2	CD79A
gdT_2	ATXN7L3B
gdT_2	SCML4
gdT_2	SPINT2
gdT_2	LEPROTL1
gdT_2	NELL2
gdT_2	SINHCAF
gdT_2	BCL11B
gdT_2	LTB
gdT_2	AC092338.1
gdT_3	FGFBP2
gdT_3	GNLY
gdT_3	EFHD2
gdT_3	NKG7
gdT_3	CST7
gdT_3	CCL5
gdT_3	GZMB
gdT_3	KIR3DL1
gdT_3	CD3G
gdT_3	PRSS23
gdT_3	IFITM2
gdT_3	TRDC
gdT_3	ZEB2
gdT_3	GZMH
gdT_3	CD247
gdT_3	CD99
gdT_3	ITGB2
gdT_3	KLRD1
gdT_3	FCGR3A
gdT_3	CALR
gdT_3	SPON2
gdT_3	CD63
gdT_3	HOPX
gdT_3	PRF1
gdT_3	ABHD17A
gdT_3	PYHIN1
gdT_3	LGALS1
gdT_3	C1orf21
gdT_3	DSTN
gdT_3	RASSF1

MAIT	TSPAN15
MAIT	ADAM12
MAIT	LTK
MAIT	SCART1
MAIT	CCR6
MAIT	SLC4A10
MAIT	DPP4
MAIT	SPRY1
MAIT	COLQ
MAIT	KIF5C
MAIT	KLRB1
MAIT	CD8A
MAIT	NCR3
MAIT	TMIGD2
MAIT	IL7R
MAIT	NFKBIA
MAIT	PHACTR2
MAIT	MAF
MAIT	ZBTB16
MAIT	SLAMF1
MAIT	PLXND1
MAIT	APOL3
MAIT	GPR171
MAIT	AQP3
MAIT	TLE1
MAIT	RORA
MAIT	TNFRSF25
MAIT	HSP90AB1
MAIT	IL18RAP
MAIT	NR1D1
B Intermediate	CIB1
B Intermediate	HSPB1
B Intermediate	LRRK2
B Intermediate	SOX5
B Intermediate	TFEC
B Intermediate	CD1C
B Intermediate	GPR183
B Intermediate	SYK
B Intermediate	PARP15
B Intermediate	FCRL2
B Intermediate	CCDC50
B Intermediate	MPP6
B Intermediate	ZEB2

B Intermediate	MPEG1
B Intermediate	RALGPS2
B Intermediate	PTPRJ
B Intermediate	ARHGAP24
B Intermediate	IL10RA
B Intermediate	CNFN
B Intermediate	EMP3
B Intermediate	HCK
B Intermediate	RIN3
B Intermediate	ARF6
B Intermediate	ENC1
B Intermediate	ZDHHC21
B Intermediate	FGR
B Intermediate	TNFRSF1B
B Intermediate	ITGAX
B Intermediate	ZFP36
B Intermediate	AKAP13
B Memory	SHISA8
B Memory	LYPLAL1
B Memory	DAAM1
B Memory	NAP1L1
B Memory	CRIP2
B Memory	COCH
B Memory	TEX9
B Memory	SCIMP
B Memory	SINHCAF
B Memory	GSTK1
B Memory	TCF7
B Memory	BLK
B Memory	S100A10
B Memory	MARCKS
B Memory	PLP2
B Memory	LTB
B Memory	ARID5B
B Memory	NFKBIA
B Memory	CR1
B Memory	AIM2
B Memory	SSPN
B Memory	HOPX
B Memory	RILPL2
B Memory	NCOA7
B Memory	CD82
B Memory	CAPG

B Memory	PIM3
B Memory	ANXA4
B Memory	CPNE5
B Memory	AHNAK
B Naive	TCL1A
B Naive	CD200
B Naive	IGHD
B Naive	IL4R
B Naive	BACH2
B Naive	COL19A1
B Naive	TSPAN13
B Naive	CD69
B Naive	BIRC3
B Naive	ITPR1
B Naive	NCK2
B Naive	CXCR4
B Naive	YBX3
B Naive	TCTN1
B Naive	SESTD1
B Naive	FCRL1
B Naive	TAPT1
B Naive	STK17A
B Naive	PLEKHA1
B Naive	FCER2
B Naive	BCL7A
B Naive	BTG1
B Naive	ATF7IP
B Naive	PLEKHA2
B Naive	AFF3
B Naive	CD37
B Naive	PNRC1
B Naive	APLP2
B Naive	MEF2C
B Naive	CCR7
Plasmablast	MZB1
Plasmablast	JCHAIN
Plasmablast	SEC11C
Plasmablast	SDF2L1
Plasmablast	PPIB
Plasmablast	PRDX4
Plasmablast	OSTC
Plasmablast	SEC61B
Plasmablast	SSR4

Plasmablast	TMED9
Plasmablast	ERLEC1
Plasmablast	SPCS2
Plasmablast	SPCS1
Plasmablast	SUB1
Plasmablast	CLPTM1L
Plasmablast	TMBIM6
Plasmablast	SSR1
Plasmablast	SELENOS
Plasmablast	TRAM1
Plasmablast	PRDX5
Plasmablast	MYDGF
Plasmablast	P4HB
Plasmablast	LMAN2
Plasmablast	SRGN
Plasmablast	UBE2J1
Plasmablast	PDIA6
Plasmablast	RABAC1
Plasmablast	TMEM208
Plasmablast	PRDX1
Plasmablast	TMEM258
ASDC	SIGLEC6
ASDC	AXL
ASDC	LTK
ASDC	PPP1R14A
ASDC	OTULINL
ASDC	APEX1
ASDC	S100A10
ASDC	CALM1
ASDC	UPK3A
ASDC	MYL12A
ASDC	FCGRT
ASDC	SCT
ASDC	HINT1
ASDC	PTPRE
ASDC	DAB2
ASDC	SOX4
ASDC	ALDH2
ASDC	ANXA2
ASDC	CCND3
ASDC	TNNI2
ASDC	VASH1
ASDC	KIF2A

ASDC	C20orf27
ASDC	PHB
ASDC	LGMN
ASDC	SNRNP25
ASDC	SULF2
ASDC	RAB11FIP1
ASDC	DPYSL2
CD14 Mono	STEAP4
CD14 Mono	S1PR3
CD14 Mono	CLEC4D
CD14 Mono	MCEMP1
CD14 Mono	CRISPLD2
CD14 Mono	F5
CD14 Mono	CYP27A1
CD14 Mono	PROK2
CD14 Mono	DYSF
CD14 Mono	AQP9
CD14 Mono	ALDH1A1
CD14 Mono	CYP1B1
CD14 Mono	PLA2G7
CD14 Mono	DSC2
CD14 Mono	NRG1
CD14 Mono	CATSPER1
CD14 Mono	HOMER3
CD14 Mono	CD163
CD14 Mono	RBP7
CD14 Mono	HPSE
CD14 Mono	FCAR
CD14 Mono	THBS1
CD14 Mono	NLRP12
CD14 Mono	CLEC4E
CD14 Mono	CDA
CD14 Mono	CR1
CD14 Mono	NRGN
CD14 Mono	VNN2
CD14 Mono	STAB1
CD14 Mono	PLPPR2
CD16 Mono	LYPD2
CD16 Mono	PPP1R17
CD16 Mono	CASP5
CD16 Mono	IGFBP6
CD16 Mono	L1TD1
CD16 Mono	CEACAM3

CD16 Mono	TNFRSF8
CD16 Mono	TMTC1
CD16 Mono	ABCC3
CD16 Mono	CTSL
CD16 Mono	C1QB
CD16 Mono	C5AR2
CD16 Mono	C1QA
CD16 Mono	CDKN1C
CD16 Mono	HES4
CD16 Mono	CHST7
CD16 Mono	HEG1
CD16 Mono	ADGRE1
CD16 Mono	FCGR3A
CD16 Mono	VMO1
CD16 Mono	MRAS
CD16 Mono	PPM1N
CD16 Mono	KCNMA1
CD16 Mono	SPRED1
CD16 Mono	NEURL1
CD16 Mono	MSR1
CD16 Mono	TCF7L2
CD16 Mono	MTSS1
CD16 Mono	TESC
CD16 Mono	CKB
cDC1	CADM1
cDC1	CLEC9A
cDC1	CPNE3
cDC1	SNX3
cDC1	CLNK
cDC1	GSTP1
cDC1	ENPP1
cDC1	MZT2A
cDC1	CPVL
cDC1	RGS10
cDC1	CD74
cDC1	HLA-DPA1
cDC1	GYPC
cDC1	HLA-DPB1
cDC1	HLA-DRA
cDC1	BATF3
cDC1	HLA-DQB1
cDC1	C1orf54
cDC1	BASP1

cDC1	SUB1
cDC1	CST3
cDC1	WDFY4
cDC1	MCUR1
cDC1	HLA-DOB
cDC1	NDUFV2
cDC1	RAB32
cDC1	CYB5R3
cDC1	FNBP1
cDC1	HDAC9
cDC1	HLA-DQA1
cDC2	CD1E
cDC2	IL1R2
cDC2	CD1C
cDC2	FCGR2B
cDC2	ID3
cDC2	FCER1A
cDC2	RTN1
cDC2	CACNA2D3
cDC2	CLEC10A
cDC2	PKIB
cDC2	ENHO
cDC2	TMEM273
cDC2	LMNA
cDC2	GPAT3
cDC2	PID1
cDC2	CCND2
cDC2	CST7
cDC2	ID1
cDC2	IL13RA1
cDC2	CD33
cDC2	IL18
cDC2	ATP1B1
cDC2	RCC2
cDC2	FAM102B
cDC2	TOB1
cDC2	F13A1
cDC2	LY86
cDC2	MAT2A
cDC2	EMP1
cDC2	VEGFA
pDC	COL26A1
pDC	SHD

pDC	CRYM
pDC	ASIP
pDC	CYP46A1
pDC	AJAP1
pDC	LRRC26
pDC	SLC12A3
pDC	KRT5
pDC	PACSIN1
pDC	CUX2
pDC	TTC24
pDC	MAP1A
pDC	SLITRK5
pDC	TTC39A
pDC	PTCRA
pDC	RBMS3
pDC	CHPF
pDC	WNT10A
pDC	TCL1A
pDC	AEBP1
pDC	DPPA4
pDC	LCNL1
pDC	MYBL2
pDC	AR
pDC	PTPRS
pDC	CYYR1
pDC	PALD1
pDC	EPHA2
pDC	PHEX
