# Supplementary material for: Multimodal single-cell datasets characterize antigen-specific CD8+ T cells across SARS-CoV-2 vaccination and infection
Source: Nat Immunol. 2023 Sep 21;24(10):1725–34. doi: 10.1038/s41590-023-01608-9 (PMC10522491; doi:10.1038/s41590-023-01608-9)
Supplement: Supplementary file 2 — Reporting Summary [file 41590_2023_1608_MOESM2_ESM.pdf]

nature portfolio

Corresponding author(s): Rahul Satija

Last updated by author(s): Jul 24, 2023

Reporting Summary

Nature Portfolio wishes to improve the reproducibility of the work that we publish. This form provides structure for consistency and transparency in reporting. For further information on Nature Portfolio policies, see our Editorial Policies and the Editorial Policy Checklist.

Statistics

For all statistical analyses, confirm that the following items are present in the figure legend, table legend, main text, or Methods section.

n/a	Confirmed	
☐	☒	The exact sample size (*n*) for each experimental group/condition, given as a discrete number and unit of measurement
☐	☒	A statement on whether measurements were taken from distinct samples or whether the same sample was measured repeatedly
☐	☒	The statistical test(s) used AND whether they are one- or two-sided<br>*Only common tests should be described solely by name; describe more complex techniques in the Methods section.*
☐	☒	A description of all covariates tested
☐	☒	A description of any assumptions or corrections, such as tests of normality and adjustment for multiple comparisons
☐	☒	A full description of the statistical parameters including central tendency (e.g. means) or other basic estimates (e.g. regression coefficient) AND variation (e.g. standard deviation) or associated estimates of uncertainty (e.g. confidence intervals)
☐	☒	For null hypothesis testing, the test statistic (e.g. *F*, *t*, *r*) with confidence intervals, effect sizes, degrees of freedom and *P* value noted<br>*Give P values as exact values whenever suitable.*
☐	☒	For Bayesian analysis, information on the choice of priors and Markov chain Monte Carlo settings
☐	☒	For hierarchical and complex designs, identification of the appropriate level for tests and full reporting of outcomes
☐	☒	Estimates of effect sizes (e.g. Cohen's *d*, Pearson's *r*), indicating how they were calculated

Our web collection on statistics for biologists contains articles on many of the points above.

Software and code

Policy information about availability of computer code

Data collection	BD FACSDiva (v 8.0.1)
Data analysis	Softwares used in the study:<br>FlowJo 10.8.1 (Treestar)<br>R packages:<br>Seurat v4<br>Signac v1.8.0<br>Alevin v1.8.0<br>Cellranger v6.0<br>Cellranger-atac v2.0<br>Milo v1.5.0<br>CoNGA v0.1.1<br>scRepertoire v1.7.2

For manuscripts utilizing custom algorithms or software that are central to the research but not yet described in published literature, software must be made available to editors and reviewers. We strongly encourage code deposition in a community repository (e.g. GitHub). See the Nature Portfolio guidelines for submitting code & software for further information.

Data

Policy information about availability of data

All manuscripts must include a data availability statement. This statement should provide the following information, where applicable:

- Accession codes, unique identifiers, or web links for publicly available datasets
- A description of any restrictions on data availability
- For clinical datasets or third party data, please ensure that the statement adheres to our policy

The datasets generated in this study are available as open-access downloads at: https://zenodo.org/record/7555405

The scATAC-seq data of tivalent inactivated seasonal influenza vaccine:
https://www.ncbi.nlm.nih.gov/geo/query/acc.cgi?acc=GSE165906

The public vaccine CITE-seq dataset used in Extended Data Fig. 3:
https://www.ncbi.nlm.nih.gov/geo/query/acc.cgi?acc=GSE171964

The scRNA-seq dataset of acute SARS-CoV-2 infection used in Figure 4:
https://zenodo.org/record/5770747

The datasets from COvid-19 Multi-omics Blood ATlas (COMBAT):
https://zenodo.org/record/6120249

Human research participants

Policy information about studies involving human research participants and Sex and Gender in Research.

Reporting on sex and gender	Gender and other demographics are reported in Supplementary Table 1.
Population characteristics	PBMC were collected from observational studies of adults who were receiving BNT162b2 vaccination and willing to participate, excluding individuals with severe anemia or inability to comply with procedures. The specific subset of donors included 12 females and 4 males of variable racial and ethnic background, ages ranging 17-58. All groups were provided with written consent for enrollment with approval from the New York University (NYU) Institutional Review Board (across protocols 18-02035, 18-02037, and 12-01137). Detailed characteristics of the population in this study are provided in Supplementary Table 1.
Recruitment	Inclusion Criteria:<br>1. Adults and children ages 0 - 110<br>a. Including breastfeeding and pregnant women<br>2. Must be able to understand and sign the Informed Consent Form (ICF) or Assent Form (for individuals < 18 years of age)<br>3. Must be able to understand and sign the HIPAA authorization form<br>Exclusion Criteria:<br>1. Known clinically significant anemia (i.e., Hb < 10 g/dL)<br>2. Contraindication to phlebotomy based on investigator judgement; i.e., anticoagulation therapy or clinically significant thrombocytopenia<br>3. Any condition that, in the opinion of the Investigator, would make study participation unsafe or would interfere with the objectives of the study
Ethics oversight	New York University (NYU) Institutional Review Board (across protocols 18-02035, 18-02037, and 12-01137).

Note that full information on the approval of the study protocol must also be provided in the manuscript.

Field-specific reporting

Please select the one below that is the best fit for your research. If you are not sure, read the appropriate sections before making your selection.

☒ Life sciences ☐ Behavioural & social sciences ☐ Ecological, evolutionary & environmental sciences

For a reference copy of the document with all sections, see nature.com/documents/nr-reporting-summary-flat.pdf

Life sciences study design

All studies must disclose on these points even when the disclosure is negative.

Sample size	Our study recruited a total of 12 donors. No statistical method was used to determine the sample size.

Data exclusions	Cells are of low data quality were excluded from the analysis (Supplementary Methods)
Replication	Experiments were conducted with multiple replicates, all of which yielded comparable results. Specifically, the CITE-seq experiment was performed with four replicates, while both the ASAP-seq and ECCITE-seq experiments were conducted with two replicates each.
Randomization	Randomization was not applicable in this case as the study did not involve any interventions.
Blinding	Blinding was not applicable in this case as the study did not involve any interventions.

Reporting for specific materials, systems and methods

We require information from authors about some types of materials, experimental systems and methods used in many studies. Here, indicate whether each material, system or method listed is relevant to your study. If you are not sure if a list item applies to your research, read the appropriate section before selecting a response.

Materials & experimental systems

n/a	Involved in the study	
☐	☒	Antibodies
☒	☐	Eukaryotic cell lines
☒	☐	Palaeontology and archaeology
☒	☐	Animals and other organisms
☒	☐	Clinical data
☒	☐	Dual use research of concern

Methods

n/a	Involved in the study	
☒	☐	ChIP-seq
☐	☒	Flow cytometry
☒	☐	MRI-based neuroimaging

Antibodies

Antibodies used	Please see Supplementary Table 2 for a full list of antibodies, clones. TotalSeq™-A Human Universal Cocktail, V1.0 (Catalog: 399907). TotalSeq™-C Human Universal Cocktail, V1.0 (Catalog: 399905)
Validation	All antibodies are commercially available and validated by the vendor: https://www.biolegend.com/en-us/products/totalseq-c-human-universal-cocktail-v1-0-19736?pdf=true&displayInline=true&leftRightMargin=15&topBottomMargin=15&filename=TotalSeq%E2%84%A2-C%20Human%20Universal%20Cocktail,%20V1.0.pdf&v=20230721091804

Flow Cytometry

Plots

Confirm that:

☒ The axis labels state the marker and fluorochrome used (e.g. CD4-FITC).

☒ The axis scales are clearly visible. Include numbers along axes only for bottom left plot of group (a 'group' is an analysis of identical markers).

☒ All plots are contour plots with outliers or pseudocolor plots.

☒ A numerical value for number of cells or percentage (with statistics) is provided.

Methodology

Sample preparation	For initial CITE-seq and ASAP-seq experiments, PBMCs from all timepoints (Day 0, 2, 10, and 28) across 3 donors (12 specimens in total) were simultaneously thawed and promptly transferred to a 96-well V-bottom plate. This enabled further processing in parallel with multichannel pipettes. The same workflow was repeated with 3 additional donors, to generate the aggregate data in Fig.s 1-2. Each aliquot of 1-3 million frozen PBMC was thawed into 10 mL complete media, centrifuged at 300 RCF for 10 minutes at 4 °C, and resuspended in 200 µL conventional cytometry buffer (PBS with 4% fetal bovine serum), DAPI, and 2mM EDTA. Samples were passed through a 70-micron filter, and single cells were sorted on a FACSAriaII (BD Biosciences) using a 100-micron nozzle. The instrument operated via FACSDiva software, with post-sort analysis performed on FlowJo 10.8.1 (Tree Star). Gating excluded cellular debris and doublets based on FSC and SSC profiles and excluded dead cells based on DAPI. Cells were collected into 5mL of complete media separately maintained on ice until all sorting concluded, at which point all tubes were simultaneously centrifuged. Individual pellets were resuspended with 100 µL of staining buffer (PBS with 2% BSA and 0.01% Tween) along with unique hashing antibodies, followed by incubation on ice for 15 minutes. Hashed samples were washed 3 times with 500 µL of staining buffer and then pooled together. Viability (greater than 92%) and final cell counts were assessed with trypan blue and Countess II FL automated counter (ThermoFisher).
Instrument	FACSARIA II running FACSDiva software; Cytek Aurora cytometer running SpectroFlow software.
Software	FlowJo 10.8.1 (Treestar).

Cell population abundance	Purity of sorted populations (dextramer-positive and CD38-positive CD8+ T cells) was confirmed by post-sort re-analysis on the Cytek Aurora. Abundance of the sorted populations, and the ratios in which they were mixed before input into downstream sequencing is described under Supplementary Methods subheadings "Dextramer validation with spectral flow cytometry" and "Enrichment of spike-specific CD8+ T cells, prior to ECCITE-seq."
Gating strategy	Voltages were adjusted to center the majority of cells in the SSC-A vs FSC-A plot, with a gate drawn in the middle to avoid cellular debris in the bottom left corner. "Singlets1" was taken by the major linear population on FSC-W vs FSC-A, and "Singlets2" was similarly taken from SSC-W vs FSC-A. Live cells were gated from DAPI vs FSC-A. Non-T cell dump gate included CD14, CD16, and CD20, and these cells were excluded, while CD2-positive cells were gated in. CD8 vs CD4 was plotted with CD8 cell population gated in. Finally, gates were drawn for Dextramer+, or CD38+HLA-DR+, as shown in Supplementary Figure 5.

☒ Tick this box to confirm that a figure exemplifying the gating strategy is provided in the Supplementary Information.
